# Microlearning versus traditional instruction for improving nursing students’ knowledge and beliefs about autism spectrum disorder: a quasi-experimental study

**DOI:** 10.1186/s12912-026-05443-9

**Published:** 2026-10-31

**Authors:** Fatma Ismael Mohammed, Muna Abualhoul, Rasha Mohamed Abohadida

**Affiliations:** 1 Pediatric Nursing, Faculty of Nursing, Benha University, Benha city, Egypt; 2 Nursing Department, Irbid National University, Irbid city, Jordan; 3 Pediatric Nursing, Faculty of Nursing, Alexandria University, Alexandria city, Egypt

**Keywords:** Autism spectrum disorder, Beliefs, Microlearning, Nursing students’ knowledge, Traditional instruction

## Abstract

**Background:**

Autism spectrum disorder (ASD) is a neurodevelopmental condition characterized by persistent difficulties in social communication and interaction, along with restricted or repetitive behaviors and interests. Microlearning presents educational content in short, focused units and may facilitate knowledge acquisition and retention. Adequate knowledge and positive beliefs about ASD are important for nurses to provide individualized and evidence-based care.

**Methods:**

A quasi-experimental, non-equivalent control group pretest–posttest design was conducted to evaluate the effectiveness of microlearning compared with traditional instruction in improving nursing students’ knowledge and beliefs regarding ASD. The study included 140 undergraduate nursing students at Irbid National University, Jordan. A convenience sampling technique was used. Students were assigned according to their existing class sections, academic and clinical schedules to either the microlearning group (*n* = 70) or the traditional instruction group (*n* = 70). Data were collected using three online tools: Tool I, a structured questionnaire for students’ characteristics and previous ASD-related educational exposure; Tool II, an ASD knowledge questionnaire; and Tool III, an ASD beliefs questionnaire. A post-test was administered one month after completion of the intervention.

**Results:**

Mixed-design repeated-measures ANOVA showed significant improvements in knowledge and beliefs over time in both groups (*p* < 0.001). Significant time × group interactions were observed for knowledge (F(1,138) = 24.23, *p* < 0.001, partial η² = 0.149) and beliefs (F(1,138) = 14.73, *p* < 0.001, partial η² = 0.096), indicating greater improvements in the microlearning group than in the traditional instruction group.

**Conclusion:**

Microlearning was associated with greater improvements in nursing students’ knowledge and beliefs regarding ASD compared with traditional instruction. These findings suggested that microlearning may be a useful educational approach for enhancing nursing students’ preparation to provide evidence-based care for children with ASD.

**Clinical trial number:**

Not applicable.

**Supplementary Information:**

The online version contains supplementary material available at https://doi.org/10.1186/s12912-026-05443-9.

## Introduction

Autism Spectrum Disorder (ASD) is a lifelong neurodevelopmental condition characterized by persistent difficulties in social communication and interaction, along with restricted and repetitive patterns of behavior [1, 2]. The global burden of ASD has increased in recent years, making it an important public health and educational concern. Current epidemiological data estimate that approximately 1 in 36 children are diagnosed with ASD, highlighting a growing need for early recognition and appropriate health and educational responses [3, 4].

Early manifestations of ASD typically emerge during the first years of life. These include delayed speech development, reduced social responsiveness, limited eye contact, and difficulties with joint attention and social engagement [5, 6]. Detecting these disorders at an early stage is important since early intervention is associated with improved developmental outcomes, including improvements in communication skills, cognitive performance, and adaptive behavior [7–9]. Early support also benefits families by reducing uncertainty and improving coping and caregiving capacity [10, 11].

In spite of established clinical guidelines recommending routine developmental surveillance, delays in ASD detection remain common in many healthcare settings [12]. These delays are often related to variations in screening practices, limited training about developmental disorders, and insufficient clinical exposure among healthcare providers [13]. This highlights the need to strengthen training and awareness about ASD within nursing education and curriculum development.

Nurses have a significant role in pediatric and community health services and are often among the first professionals to detect developmental problems during routine child care visits. Their continuous contact with children and families positions them to support early recognition, provide parental guidance, and assist with appropriate and early referral to specialized health care services [14, 15]. However, previous research shows that nursing students have gaps in knowledge and hold misconceptions about ASD, which can reduce their confidence in clinical decision-making and early detection practices [16, 17].

Traditional lecture-based nursing teaching remains widely used. Although it allows structured delivery of content, it is often teacher-centered and may not sufficiently engage students in active learning processes [18]. This approach may limit long-term knowledge retention and reduce student motivation. Therefore, there is increasing interest in learner-centered and technology-supported educational strategies that promote active engagement and improve learning outcomes.

Microlearning is an emerging educational strategy that delivers content in short, focused learning units designed for quick understanding and repeated reinforcement [19]. It is based on cognitive learning principles suggesting that smaller, well-structured learning segments reduce cognitive overload and support better memory retention over time [20, 21]. This approach also supports flexible learning by allowing students to retrieve learning materials at their own pace and time.

### Significance of the study

Autism Spectrum Disorder (ASD) is an important area of pediatric nursing care, and preparing nursing students with adequate knowledge and appropriate attitudes is essential for providing responsive and individualized care to children with ASD and their families. Recent evidence has shown that undergraduate nursing students have gaps in their knowledge and attitudes toward ASD, highlighting the need to strengthen autism-related education within nursing curricula [22]. In addition [23, 24], have reported that microlearning can improve student engagement, academic performance, and knowledge retention in nursing education. However, there is still limited evidence regarding its specific effectiveness in improving nursing students’ knowledge and beliefs about ASD.

### Theoretical framework

The present study is guided by **Cognitive Load Theory (CLT)**, which proposes that learning is more effective when information is presented in a way that matches the limited capacity of working memory [25]. In nursing education, breaking complex information into smaller and manageable units can reduce unnecessary cognitive load and facilitate knowledge acquisition and retention [26]. Based on this principle, **microlearning** provides short, focused learning units that allow students to process information gradually and reinforce their learning over time [21]. The framework, therefore assumes that presenting ASD-related content in concise and manageable learning units may facilitate cognitive processing and lead to better knowledge and more positive beliefs among nursing students.

### Aim of the study

This study aimed to evaluate the effectiveness of microlearning compared with traditional instruction on nursing students’ knowledge and beliefs regarding children with autism spectrum disorder.

### Objectives of the study

1. Assess nursing students’ knowledge and beliefs regarding ASD.
2. Design and implement a microlearning educational session according to their actual needs assessment.
3. Compare the effectiveness of microlearning and traditional instruction on nursing students’ knowledge and beliefs about children with ASD.

### Research hypothesis

Nursing students who learn through microlearning exhibit higher scores of knowledge and beliefs than those who learn through traditional instruction about children with autism spectrum disorder.

## Subject and methods

**The subject and methods of the current study were presented under the following four designs**:

### 1-Technical design

The technical design of the current study included: research design, setting, subjects, and tools of data collection.

#### A-Research design

A quasi-experimental, nonequivalent control group pretest–posttest design was used to evaluate the effectiveness of microlearning compared with traditional instruction on nursing students’ knowledge and beliefs regarding ASD. The study was reported in accordance with the Transparent Reporting of Evaluations with Nonrandomized Designs (TREND) statement.

#### B-Research setting

The study was conducted at the Faculty of Nursing, Irbid National University, Jordan.

#### C). Participants and sampling procedure

A convenience sampling technique was used to recruit undergraduate nursing students who met the following eligibility criteria: Undergraduate nursing students enrolled in the Pediatric Nursing course during the second semester of the 2025–2026 academic year who were not receiving concurrent academic instruction on autism spectrum disorder (ASD) during the study period, to minimize the potential influence of other educational exposure on the study outcomes, and who were available and willing to participate in the study. Initially, 154 students were assessed for eligibility. Fourteen students were excluded before the main study because they had participated in the pilot study, to avoid potential contamination of the study results. Therefore, 140 students were included in the main study.

Because the students had different academic and clinical schedules, group assignment was based on their existing class sections rather than on individual participants. The class sections were assigned to either the microlearning or traditional instruction group according to students’ attendance and academic and clinical schedules, while maintaining the regular course timetable. The group assignment was implemented by the research team according to the predetermined class schedules.

**Fig. 1:**
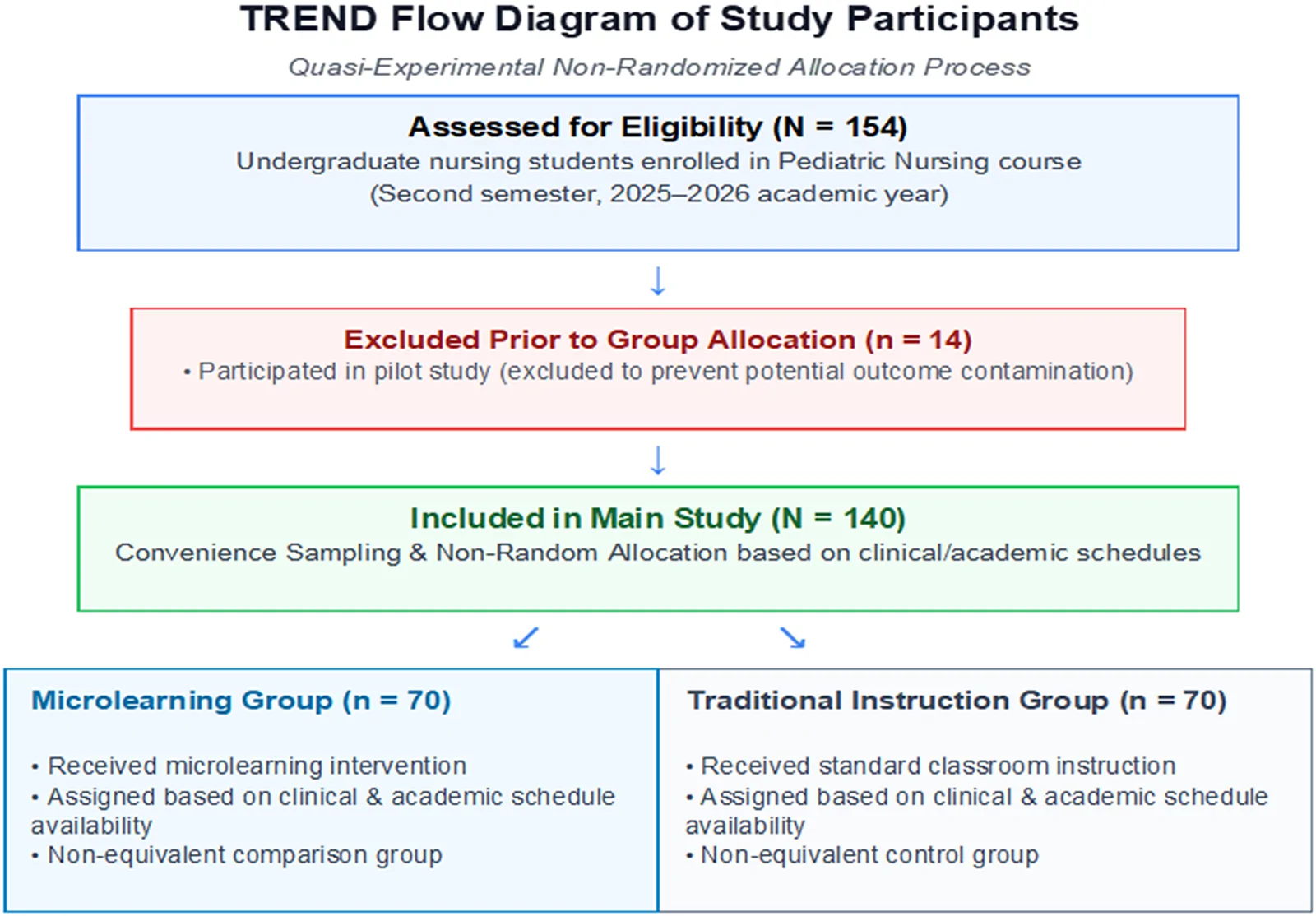
Participant flow diagram based on the TREND statement for Non-Randomized evaluations. note allocation was non-randomized to avoid timetable disruption and accommodate student clinical/academic availability

Therefore, no individual-level randomization sequence was generated, and no allocation concealment procedure was used. Accordingly, the participants were assigned to two non-equivalent groups: the Microlearning group (*n* = 70) and the traditional instruction group (*n* = 70) (See Fig. 1).

#### Sample size

Epi Info was used to estimate the required sample size, based on a total eligible population of approximately 154 undergraduate students, a 95% confidence level, a 5% acceptable margin of error, and an expected frequency of 50%. The minimum estimated sample size was 105 students. All 154 eligible students were approached to maximize the representativeness of the sample.

#### Tools of data collection

Data were gathered using the following three study tools:

##### Tool (I) An online structured interview questionnaire

This tool was developed by the researchers after reviewing relevant literature, previous studies, and research [16, 18]. It was written in the English language and concerned the characteristics of the studied nursing students.

##### Tool (II): An online nursing student ASD knowledge questionnaire

This tool was adapted from Abualhoul et al., 2024 [14] to assess pediatric nursing students’ knowledge of ASD diagnostic criteria and evidence-based clinical interventions. To support construct validity and diminish acquiescence bias, one false statement was added: “Delayed toilet training is a core diagnostic red flag of ASD.”

##### Scoring system

A scoring system was established in which each correct response was given a score of (1), and incorrect /don’t know was given a score of (0). The total score ranged from 0 to 14 points. Higher scores indicated more improvement in students’ knowledge about children with ASD.

##### Tool (III): An online nursing student beliefs questionnaire

Adapted from Abualhoul et al., 2024 [14], this online instrument measures pediatric nursing students’ beliefs regarding ASD. To prevent item redundancy with the knowledge questionnaire (Tool II), two original statements were excluded: “ASD is best diagnosed by healthcare providers using a multidisciplinary approach” and “I have a role as a healthcare provider in the early detection of ASD.” The resulting finalized scale consists of eight items.

**Scoring system**: The total beliefs score was rated using a 3-point Likert scale, ranging from 1 (Disagree), 2 (Neutral), to 3 (Agree) total score range from 8–24 point. Negatively worded statements, including:” Most children with ASD have intellectual disability, ASD occurs more commonly among children from higher socioeconomic background”, and” ASD is a preventable condition, were reverse-coded for analysis. Higher scores indicated more positive beliefs toward children with ASD.

### II- Operational design

Operational design included: preparatory phase, content validity, pilot study, reliability, and fieldwork.

#### Preparatory phase

This phase included reviewing the related literature and theoretical knowledge of various aspects of the study using books, articles, the internet, periodicals, and journals at the local and international levels to develop tools and to become acquainted with the various aspects of the research problems.

#### Content validity

The tools of data collection were examined for their content validity by two experts in pediatric nursing from the Faculty of Nursing, Benha University, and one expert in psychiatric nursing from the Faculty of Nursing, Alexandria University. They were selected to assess the content validity of the instruments and to judge their clarity, comprehensiveness, relevance, simplicity, and accuracy. All their remarks were taken into consideration; some items were rephrased to arrive at the final version of the tools. The tools were regarded as valid from the experts’ point of view.

#### Pilot study

Prior to initiating the main study, a pilot study was conducted to evaluate the clarity, comprehensibility, and applicability of the study tools and to determine the approximate time required for their completion. The pilot was conducted before administration of the pretest to participants in the main study. Fourteen students participated in the pilot study and were subsequently excluded from the main study to avoid potential contamination of the study findings. Following completion of the pilot study, the necessary modifications were made to the study tools, and the final version was administered during the pretest phase of the main study. This phase took one week.

#### Reliability

Following completion of the pilot study, the reliability of the study tools was assessed using data obtained from the 14 pilot participants. Internal consistency was evaluated using Cronbach’s alpha coefficient before administering the final versions of the tools to participants in the main study. Cronbach’s alpha coefficients were 0.98 for the 14-item knowledge questionnaire and 0.74 for the 8-item beliefs questionnaire, indicating acceptable internal consistency for both instruments.

#### Field of work

Microlearning and traditional instruction for improving nursing students’ knowledge and beliefs about autism spectrum disorder were implemented in these phases: assessment, planning, implementation, and evaluation.

##### Assessment phase

The assessment phase involved interviews with students in the micro-learning and traditional instruction groups to collect baseline data. The researcher was available three days a week. At the beginning of the interview, the researcher welcomed each student, explained the purpose, duration, and activities of the study, and obtained written consent. An online questionnaire **Link** was sent to the participating nursing students according to their group to collect data on their characteristics, knowledge, and beliefs using the previously mentioned study tools. This phase took two weeks.

#### Planning phase

Based on extensive analysis of relevant scholarly literature from journals, textbooks, bulletins, and electronic media, the researchers developed instructional microlearning content based on current evidence and reviewed by experts in pediatric and psychiatric nursing to ensure validity and improve students’ knowledge and beliefs about children with spectrum disorders. With the aid of motion graphics and video editing experts, videos were designed using animation techniques to make them more attractive to the students watching them. Microlearning videos were made using Canva Pro, Adobe Premiere Pro 2019, and Adobe After Effects 2015. In addition, PowerPoint presentations were prepared for the traditional instruction group and covered the same learning content as the microlearning group. This phase took two weeks.

#### Implementation phase

The educational intervention was implemented over a 9-week period, with 1 video/lecture every week, and consisted of nine sequential instructional sessions addressing key clinical domains of autism spectrum disorder (ASD). Both groups received equivalent educational content; however, the mode of delivery differed. The microlearning group (*n* = 70) received asynchronous instructional videos uploaded to the Moodle learning platform, with an average video duration of approximately five minutes. Students accessed the modules sequentially at their own pace and completed embedded formative questions after each module, receiving immediate automated feedback and answer rationales.

The traditional instruction group (*n* = 70) received the equivalent educational content through synchronous, 30-minute PowerPoint-based lectures. To accommodate students’ academic and clinical schedules, each weekly lecture was delivered separately to three student subgroups comprising 25, 25, and 20 students, respectively, according to their scheduled attendance days. These subgroups represented different delivery sessions within the same traditional instruction and were not considered independent study groups. Students’ understanding was assessed through instructor-led oral questioning and discussion during or at the end of each lecture. These formative activities were intended to reinforce learning and were not included as outcome measures in the statistical analysis. Supplementary Table S1**. **Session-by-Session Description of the Educational Intervention.

Following completion of the intervention, a one-month interval was allowed before the post-test was administered to both groups.

#### Contamination control

Managed via unique user logins, restricted downloading of materials, student pledges not to share content, and delaying the post-test by one month after implementation of the program to prevent recall bias.

#### Evaluation phase

Following completion of the nine-week educational intervention, a one-month interval was allowed before administering the post-test to both the microlearning and traditional instruction groups. The same validated study instruments used for the baseline assessment were administered to evaluate changes in students’ knowledge and beliefs regarding autism spectrum disorder (ASD). The one-month interval was intended to reduce the likelihood of participants recalling their previous responses and to minimize the potential testing effect.

### III - Administrative design

Official approval was obtained from the Dean of the Faculty of Nursing, Irbid National University, Jordan. A clear explanation was given about the nature, importance, and expected outcomes of the study to carry out the study with minimal resistance.

### IV -Statistical design

Data were analyzed using SPSS version 26. The statistical analyst was blinded to group allocation during the data analysis, with the intervention groups coded without revealing their identities to minimize potential analytical bias. Descriptive statistics were used to summarize the study variables. Chi-square and Fisher’s exact tests were used for categorical data, while independent- and paired-samples t-tests were used between and within-group comparisons, respectively. The McNemar test was used to assess changes in individual dichotomous knowledge items. Two-way repeated-measures ANOVA was performed to assess changes over time, between-group differences, and the time × group interaction. Effect sizes were reported using partial eta squared (ηp²), as appropriate. Statistical significance was set at *p* < 0.05.

## Results

Table 1. Baseline demographic and academic characteristics were comparable between groups, with no statistically significant differences observed in age, gender, academic enrollment, prior ASD coursework, ASD-related training, family history of ASD, or direct experience with children with ASD (all *p* > 0.05). These findings indicate baseline equivalence between the two groups.

Table 2. Presents the changes in the proportion of correct responses to students’ knowledge regarding autism spectrum disorder from pretest to posttest in both study groups. At pretest, the proportions of correct responses were low and relatively comparable between the microlearning and traditional instruction groups, ranging from 8.6% to 20.0% and from 7.1% to 17.1%, respectively. Following the intervention, significant improvements were observed across all knowledge items in both groups (McNemar test, *p* < 0.001). The microlearning group showed a greater improvement, with posttest correct responses ranging from 82.9% to 94.3%, compared with 55.7% to 68.6% in the traditional instruction group. Thus, the posttest percentages were approximately 24.0 to 30.0% points higher in the microlearning group across the students’ knowledge items.

Table 3. Presents the changes in the proportion of correct responses to knowledge items regarding ASD interventions from pretest to posttest in both study groups. At pretest, the proportions of correct responses were generally low and relatively comparable between the microlearning and traditional instruction groups, ranging from 5.7% to 18.6% and from 8.6% to 18.6%, respectively. Following the intervention, significant improvements were observed across all items in both groups (McNemar test, *p* < 0.001). In the microlearning group, the proportion of correct responses increased to 84.3–95.7%, compared with 68.6–74.3% in the traditional instruction group.

Table 4. Demonstrates that the two groups had comparable knowledge scores at baseline, with mean scores of 1.84 ± 1.81 for the microlearning group and 1.74 ± 1.50 for the traditional instruction group. After the intervention, knowledge scores increased in both groups, with greater improvement in the microlearning group (12.50 ± 2.95) than in the traditional instruction group (9.09 ± 4.21). The mixed-design repeated-measures ANOVA revealed a statistically significant main effect of time, F(1,138) = 714.725, *p* < 0.001, partial η² = 0.838, indicating a significant improvement in knowledge over time. A statistically significant time × group interaction was also observed, F(1,138) = 24.231, *p* < 0.001, partial η² = 0.149, indicating that the magnitude of improvement in knowledge differed significantly between the two groups, with greater improvement in the microlearning group. A significant main effect of group was also detected, F(1,138) = 26.801, *p* < 0.001, partial η² = 0.163.

Table 5. Shows that the total belief score increased significantly in both groups, from 10.90 ± 3.83 to 22.89 ± 2.56 in the microlearning group (t = 21.91, *p* < 0.001), and from 11.10 ± 3.93 to 19.81 ± 3.85 in the traditional instruction group (t = 13.33, *p* < 0.001). The improvement was greater in the microlearning group, with a mean increase of 11.99 points compared with 8.71 points in the traditional instruction group. Overall, these findings indicate that both educational approaches significantly improved students’ beliefs toward children with ASD, with a greater improvement observed following microlearning.

Table 6. The findings demonstrated a significant improvement in students’ belief scores over time in both study groups. The mean beliefs score increased from 10.90 ± 3.83 at pretest to 22.88 ± 2.55 at posttest in the microlearning group, compared with an increase from 11.10 ± 3.93 to 19.81 ± 3.84 in the traditional learning group. The repeated-measures instruction revealed a statistically significant time effect, F(1, 138) = 589.75, *p* < 0.001, partial η² = 0.810, indicating a large overall change in belief scores over time. A significant between-group effect was also observed, F(1, 138) = 11.08, *p* = 0.001, partial η² = 0.074. Importantly, the significant time × group interaction, F(1, 138) = 14.73, *p* < 0.001, partial η² = 0.096, indicated that the magnitude of improvement in beliefs differed significantly between the two groups, with a greater improvement observed among students who received the microlearning intervention. The estimated marginal means and their 95% confidence intervals further supported the observed differences between the groups across the two assessment phases.

**Table 1:** Distribution of participant demographic and educational characteristics (*n* = 140)

Items	Microlearning group (*n* = 70)	Traditional instruction group (*n* = 70)	Test	*P* -value
**Age/ years** 19 ≤ 24 25 ≤ 29 30 ≤ 35 More than 35	52(74.3) 11(15.7) 6 (8.6) 1 (1.4)	49(70.0) 13(18.6) 6(8.6) 2(2.9)	**FET** 0.724	0.939
Mean ± SD	23.88 ± 4.49	24.20 ± 4.65	t test= 0.406	0.685
**Gender** Male Female	34(48.6) 36(51.4)	38(54.3) 32(45.7)	**X2** 0.458	0.499
**Academic Enrollment Status** Regular program Bridging program	42(60.0) 28(40.0)	40(57.1) 30(42.9)	**X2** 0.118	0.731
**Have you previously studied content related to ASD in any course?** Yes No	7(10.0) 63(90.0)	9(12.9) 61(87.1)	**X2** 0.282	0.595
**Do you have a family member or relative diagnosed with ASD?** Yes No	4(5.7) 66(94.3)	6(8.6) 64(91.4)	**X2** 0.431	0.512
**Have you attended any ASD-related training**,** workshop**,** or seminar?** Yes No	8(11.4) 62(88.6)	6(8.6) 64(91.4)	**X2** 0.317	0.573
**Have you ever provided direct care or interaction with a person with ASD?** Yes No	10(14.3) 60(85.7)	7(10.0) 63(90.0)	**X2** 0.603	0.438

χ2: Chi-square test FET Fissure Exact Test t: independent sample t test *: Statistically significant at *p* ≤ 0.05

**Table 2:** Distribution of participant knowledge regarding core characteristics and red flags of ASD (*n* = 140)

Items	Microlearning group (*n* = 70)		McNemar Test *P* -value (1)	Traditional instruction group (*n* = 70)		McNemar Test *P* -value (2)
	Pretest	Post test		Pretest	Post test	
	Correct answer	Correct answer		Correct answer	Correct answer	
Children with ASD may show deficits in social reciprocity, such as difficulty with back-and-forth conversation.	9(12.9)	64(91.4)	< 0.001	11(15.7)	46(65.7)	< 0.001
Walking problems, such as difficulty climbing stairs, are a core feature of ASD	8(11.4)	66(94.3)	< 0.001	7(10.0)	48(68.6)	< 0.001
Deficits in nonverbal communication, such as limited eye contact, are common in ASD	10(14.3)	64(91.4)	< 0.001	8(11.4)	48(68.6)	< 0.001
Difficulties in developing and maintaining relationships are characteristic of ASD	9(12.9)	59(84.3)	< 0.001	10(14.3)	42(60.0)	< 0.001
Repetitive behaviors, such as lining up toys, may be observed in children with ASD	9(12.9)	58(82.9)	< 0.001	8(11.4)	39(55.7)	< 0.001
Rigid adherence to routines, such as insisting on taking the same route to school, is common in ASD	11(15.7)	66(94.3)	< 0.001	9(12.9)	45(64.3)	< 0.001
Children with ASD may display highly restricted or fixated interests, such as focusing on specific objects only.	14(20.0)	64(91.4)	< 0.001	12(17.1)	46(65.7)	< 0.001
Unusual responses to sensory input, such as covering ears in response to certain sounds, are associated with ASD	12(17.1)	62(88.6)	< 0.001	11(15.7)	43(61.4)	< 0.001
Delayed toilet training is a core diagnostic red flag of ASD	6(8.6)	60(85.7)	< 0.001	5(7.1)	40(57.1)	< 0.001

McNemar Test *: Statistically significant at *p* ≤ 0.05 P -value (1) between pretest and posttest of the micro learning group, P -value (2) between pretest and posttest of the traditional learning group

**Table 3:** Distribution of Participant knowledge regarding interventions for ASD (*n* = 140)

Items	Microlearning group (*n* = 70)		McNemar Test *P* -value (1)	Traditional instruction group (*n* = 70)		McNemar Test *P* -value (2)
	Pretest	Post test		Pretest	Post test	
	Correct answer	Correct answer		Correct answer	Correct answer	
Psychosocial interventions play an important role in the management of ASD	13(18.6)	65(92.9)	< 0.001	13(18.6)	50(71.4)	< 0.001
Pharmacological treatment may be used to manage associated symptoms of ASD.	8(11.4)	67(95.7)	< 0.001	10(14.3)	52(74.3)	< 0.001
Dietary interventions alone are an evidence-based treatment for ASD	7(10.0)	59(84.3)	< 0.001	7(10.0)	49(70.0)	< 0.001
Hyperbaric oxygen therapy is an evidence-based treatment for ASD	4(5.7)	63(90.0)	< 0.001	6(8.6)	48(68.6)	< 0.001
Behavioral, developmental, and multidisciplinary educational interventions are effective approaches for managing ASD	9(12.9)	58(82.9)	< 0.001	5(7.1)	40(57.1)	< 0.001

McNemar Test *: Statistically significant at *p* ≤ 0.05. P-value (1) between pretest and posttest of the microlearning group; P-value (2) between pretest and posttest of the traditional instruction group

**Table 4:** Comparison of nursing students’ knowledge toward children with ASD before and after the educational intervention between the study groups

Items	Microlearning group (*n* = 70) Mean ± SD Total (0–14)	Traditional instruction group (*n* = 70) Mean ± SD Total (0–14)	F	df	*p*-value	Partial η²	95% CI for Estimated Marginal Mean
Pretest phase	1.84 ± 1.81	1.74 ± 1.50	—		—	—	1.450–2.235 / 1.350–2.135
Posttest phase	±2.9512.50	± 4.21 9.09	—		—	—	11.640–13.360 / 8.226–9.946
Time effect	—	—	714.725	1, 138	< 0.001	0.838	-
Group effect	—	—	26.801	1, 138	< 0.001	0.163	
Time × Group interaction	—	—	24.231	1, 138	< 0.001	0.149	-

Note. Mixed-design repeated-measures ANOVA was used to examine the effects of time (pretest vs. posttest), group (microlearning vs. traditional instruction), and the time × group interaction

For each phase, the first CI corresponds to the microlearning group and the second to the traditional instruction group

**Table 5:** Comparison of participants’ total beliefs regarding ASD within microlearning and traditional instruction groups (*n* = 140)

Items	Microlearning group (*n* = 70)		Paired t-test	*P* -value	Traditional instruction group (*n* = 70)		Paired t-test	*P* -value
	Pretest	Posttest			Pretest	Posttest		
	Mean score	Mean score			Mean score	Mean score		
ASD is a neurodevelopmental disorder.	1.34±0.535	2.90±0.302	20.00	0.001	1.38±0.572	2.50±0.583	12.8	0.001
Most children with ASD have intellectual disability.	1.28±0.455	2.78±0.535	18.01	0.001	1.34±0.527	2.42±0.649	10.95	0.001
ASD occurs more commonly among children from higher socioeconomic backgrounds.	1.30±0.461	2.78±0.507	18.04	0.001	1.35±0.539	2.35±0.614	10.03	0.001
ASD is often under-recognized and missed in general healthcare practice.	1.45±0.629	2.84±0.366	16.37	0.001	1.44±0.580	2.48±0.607	10.36	0.001
There is insufficient awareness of ASD among healthcare providers.	1.41±0.551	2.92±0.259	20.07	0.001	1.40±0.574	2.57±0.603	12.28	0.001
ASD is diagnosed more frequently in males than in females.	1.38±0.572	2.94±0.233	20.72	0.001	1.42±0.603	2.58±0.577	11.95	0.001
ASD is a lifelong condition.	1.44±0.628	2.91±0.329	17.68	0.001	1.40±0.574	2.54±0.652	10.75	0.001
ASD is a preventable condition.	1.27±0.447	2.78±0.535	18.18	0.001	1.34±0.507	2.34±0.656	10.03	0.001
Total score	10.90 ± 3.83	22.88 ± 2.55	21.91	0.001	11.10 ± 3.93	19.81 ± 3.84	13.33	0.001

**Table 6:** Comparison of nursing students’ beliefs toward children with ASD before and after the educational intervention between the study groups

phase	Microlearning group (*n* = 70) Mean ± SD Total (8–24)	Traditional instruction group (*n* = 70) Mean ± SD Total (8–24)	F	df	*p*-value	Partial η²	95% CI for Estimated Marginal Mean
Pretest phase	10.90 ± 3.83	11.10 ± 3.93	—		—	—	Microlearning: 9.982–11.818; Traditional: 10.182–12.018
Posttest phase	± 2.5522.88	± 3.8419.81	—		—	—	Microlearning22.114–23.657; Traditional: 19.043–20.586
Time effect	—	—	589.75	1, 138	0.001	0.810	-
Group effect	—	—	11.08	1, 138	0.001	0.074	-
Time × Group interaction	—	—	14.73	1, 138	0.001	0.096	-

Note. Mixed-design repeated-measures ANOVA was used to examine the effects of time (pretest vs. posttest), group (microlearning vs. traditional instruction), and the time × group interaction

## Discussion

This study examined the effectiveness of microlearning compared with traditional instruction on nursing students’ knowledge and beliefs regarding children with ASD. The findings demonstrated that both teaching methods significantly improved students’ outcomes; however, microlearning produced greater gains in both knowledge and beliefs.

The superior performance of the microlearning group may be explained by its instructional structure. Microlearning delivers content in short, focused units that reduce cognitive load and enhance.

information processing and retention. This approach allows repeated exposure to key concepts and supports self-paced learning [27], which is particularly important in complex clinical topics such as ASD. These findings are consistent with [28], who reported that microlearning enhances learning efficiency and engagement in health professions education through flexible and targeted content delivery. Similarly [23], found in a systematic review that microlearning significantly improves academic performance and knowledge retention across higher education settings.

In addition, the improvement in ASD related knowledge observed in this study aligns with [29], who demonstrated that short, structured learning interventions enhance comprehension and support long-term retention of complex scientific content. These findings suggest that breaking down ASD content into smaller learning segments may help nursing students better understand diagnostic criteria and intervention principles.

Regarding beliefs toward children with ASD, both groups showed improvement; however, the microlearning group demonstrated a more pronounced positive change. This finding is important because beliefs are key components of professional competence in pediatric nursing practice. The observed improvement may be related to the learner-centered nature of microlearning, which encourages active engagement, reflection, and continuous interaction with educational content. These elements are known to influence both cognitive and affective learning domains.

These results are supported by [30], who found that digital and interactive learning environments improve learners’ attitudes and learning experiences [21]. also reported that microlearning increases learner engagement and positively influences attitudes toward complex health topics, including ASD. Furthermore [24], highlighted that digital learning strategies improve student engagement and contribute to the development of essential professional skills in health education.

Although traditional instruction remains valuable for providing structured knowledge and direct teacher–student interaction, it may promote passive learning when used alone. In contrast, microlearning encourages active participation and continuous reinforcement, which may explain its stronger impact in this study. However [31], reported no significant differences between microlearning and traditional approaches in some educational outcomes, suggesting that effectiveness may depend on instructional design quality, learner characteristics, and content complexity.

Overall, the findings of this study support microlearning as an effective and practical educational strategy in nursing education. It is particularly useful for improving learning outcomes in complex pediatric topics such as ASD, where both knowledge acquisition and attitude development are essential for clinical competence.

## Conclusion

This study concluded that the research hypothesis has been met; nursing students who learn through microlearning exhibit higher scores of knowledge and beliefs than those who learn through traditional instruction regarding children with autism spectrum disorder.

## Recommendations

- Integrate microlearning and structured digital learning modules into pediatric nursing curricula to enhance clinical knowledge and promote positive beliefs toward children with ASD.
- Future randomized controlled trials with larger sample sizes and longer follow-up times are also necessary to verify the long-term effects of microlearning on improving the knowledge and beliefs of nursing students about children with autism spectrum disorder.

## Limitations of the study

The present study has some limitations that should be considered when interpreting the findings.

- Participants were recruited using a non-probability convenience sampling from single faculty of nursing, which may limit the generalizability of the findings to nursing students in other educational settings.
- The quasi-experimental design cannot completely rule out the possibility of residual confounding, despite efforts to ensure comparability between the groups and to use the same educational content and assessment procedures.
- Student and researcher blinding was not feasible because of the nature of the educational interventions; the statistical analyst was blinded to group allocation during the data analysis. The groups were coded without revealing their intervention identities to minimize potential analytical bias.

## Supplementary Information

Below is the link to the electronic supplementary material.

Supplementary Material 1

Supplementary Material 2

Supplementary Material 3

Supplementary Material 4

Supplementary Material 5

Supplementary Material 6

Supplementary Material 7

## Data Availability

Data Availability Statement The datasets generated and/or analyzed during the current study are not publicly available because they contain confidential participant information but are available from the corresponding author on reasonable request.

## Abbreviations

ASD: Autism Spectrum Disorder
TREND: Transparent Reporting of Evaluations with Nonrandomized Designs
DSM-5: Diagnostic and Statistical Manual of Mental Disorders, Fifth Edition
SCD: Social (Pragmatic) Communication Disorder
ADHD: Attention-Deficit/Hyperactivity Disorder
ID: Intellectual Disability
M-CHAT-R/F: Modified Checklist for Autism in Toddlers, Revised with Follow-Up
ASQ: Ages and Stages
