# Supplementary material for: Microlearning versus traditional instruction for improving nursing students’ knowledge and beliefs about autism spectrum disorder: a quasi-experimental study
Source: BMC Nurs. 2026 Oct 31;25:1050. doi: 10.1186/s12912-026-05443-9 (PMC13726028; doi:10.1186/s12912-026-05443-9)

```
CROSSTABS
 /TABLES=age education previous family attend provided BY group
 /FORMAT=AVALUE TABLES
 /STATISTICS=CHISQ CORR
 /CELLS=COUNT ROW COLUMN TOTAL
 /COUNT ROUND CELL
 /METHOD=EXACT TIMER(5).
```

Crosstabs

Notes

Output Created		19-SEP-2026 09:15:12
Comments		
Input	Data	F:\\ \asd\ \major.revision.9.2026\ASD_Deidentified_Dataset.sav
	Active Dataset	DataSet1
	Filter	<none>
	Weight	<none>
	Split File	<none>
	N of Rows in Working Data File	140
Missing Value Handling	Definition of Missing	User-defined missing values are treated as missing.
	Cases Used	Statistics for each table are based on all the cases with valid data in the specified range(s) for all variables in each table.
Syntax		CROSSTABS /TABLES=age education previous family attend provided BY group /FORMAT=AVALUE TABLES /STATISTICS=CHISQ CORR /CELLS=COUNT ROW COLUMN TOTAL /COUNT ROUND CELL /METHOD=EXACT TIMER(5).

Notes

Resources	Processor Time	00:00:00.09
	Elapsed Time	00:00:00.13
	Dimensions Requested	2
	Cells Available	524245
	Time for Exact Statistics	0:00:00.09

Case Processing Summary

	Cases					
	Valid		Missing		Total	
	N	Percent	N	Percent	N	Percent
age * group	140	100.0%	0	0.0%	140	100.0%
Academic Enrollment Status * group	140	100.0%	0	0.0%	140	100.0%
previously studied content related to ASD * group	140	100.0%	0	0.0%	140	100.0%
family member or relative diagnosed with ASD * group	140	100.0%	0	0.0%	140	100.0%
attended any ASD-related training, workshop, or seminar * group	140	100.0%	0	0.0%	140	100.0%
provided direct care or interaction with a person with ASD * group	140	100.0%	0	0.0%	140	100.0%

age * group

Crosstab

			group		
			micro learning	traditional	Total
age	19-24	Count	52	49	101
		% within age	51.5%	48.5%	100.0%
		% within group	74.3%	70.0%	72.1%
		% of Total	37.1%	35.0%	72.1%
	25-30	Count	11	13	24
		% within age	45.8%	54.2%	100.0%
		% within group	15.7%	18.6%	17.1%
		% of Total	7.9%	9.3%	17.1%
	30-35	Count	6	6	12
		% within age	50.0%	50.0%	100.0%
		% within group	8.6%	8.6%	8.6%
		% of Total	4.3%	4.3%	8.6%
	more than 35	Count	1	2	3
		% within age	33.3%	66.7%	100.0%
		% within group	1.4%	2.9%	2.1%
		% of Total	0.7%	1.4%	2.1%
Total		Count	70	70	140
		% within age	50.0%	50.0%	100.0%
		% within group	100.0%	100.0%	100.0%
		% of Total	50.0%	50.0%	100.0%

Chi-Square Tests

	Value	df	Asymptotic Significance (2-sided)	Exact Sig. (2-sided)	Exact Sig. (1-sided)
Pearson Chi-Square	.589[a]	3	.899	.939	
Likelihood Ratio	.596	3	.897	.939	
Fisher's Exact Test	.724			.939	
Linear-by-Linear Association	.327[b]	1	.567	.649	.325
N of Valid Cases	140				

Chi-Square Tests

	Point Probability
Pearson Chi-Square	
Likelihood Ratio	
Fisher's Exact Test	
Linear-by-Linear Association	.077
N of Valid Cases	

a. 2 cells (25.0%) have expected count less than 5. The minimum expected count is 1.50.

b. The standardized statistic is .572.

Symmetric Measures

		Value	Asymptotic Standard Error[a]	Approximate T[b]	Approximate Significance
Interval by Interval	Pearson's R	.049	.084	.571	.569[c]
Ordinal by Ordinal	Spearman Correlation	.048	.084	.563	.574[c]
N of Valid Cases		140			

Symmetric Measures

		Exact Significance
Interval by Interval	Pearson's R	.649
Ordinal by Ordinal	Spearman Correlation	.587
N of Valid Cases		

a. Not assuming the null hypothesis.

b. Using the asymptotic standard error assuming the null hypothesis.

c. Based on normal approximation.

Academic Enrollment Status * group

Crosstab

			group	
			micro learning	traditional
Academic Enrollment Status	regular	Count	42	40
		% within Academic Enrollment Status	51.2%	48.8%
		% within group	60.0%	57.1%
		% of Total	30.0%	28.6%
	bridging	Count	28	30
		% within Academic Enrollment Status	48.3%	51.7%
		% within group	40.0%	42.9%
		% of Total	20.0%	21.4%
Total		Count	70	70
		% within Academic Enrollment Status	50.0%	50.0%
		% within group	100.0%	100.0%
		% of Total	50.0%	50.0%

Crosstab

			Total
Academic Enrollment Status	regular	Count	82
		% within Academic Enrollment Status	100.0%
		% within group	58.6%
		% of Total	58.6%
	bridging	Count	58
		% within Academic Enrollment Status	100.0%
		% within group	41.4%
		% of Total	41.4%
Total		Count	140
		% within Academic Enrollment Status	100.0%
		% within group	100.0%
		% of Total	100.0%

Chi-Square Tests

	Value	df	Asymptotic Significance (2-sided)	Exact Sig. (2-sided)	Exact Sig. (1-sided)
Pearson Chi-Square	.118[a]	1	.731	.864	.432
Continuity Correction[b]	.029	1	.864		
Likelihood Ratio	.118	1	.731	.864	.432
Fisher's Exact Test				.864	.432
Linear-by-Linear Association	.117[c]	1	.732	.864	.432
N of Valid Cases	140				

Chi-Square Tests

	Point Probability
Pearson Chi-Square	
Continuity Correction[b]	
Likelihood Ratio	
Fisher's Exact Test	
Linear-by-Linear Association	.128
N of Valid Cases	

a. 0 cells (.0%) have expected count less than 5. The minimum expected count is 29.00.

b. Computed only for a 2x2 table

c. The standardized statistic is .342.

Symmetric Measures

		Value	Asymptotic Standard Error[a]	Approximate T[b]	Approximate Significance
Interval by Interval	Pearson's R	.029	.084	.341	.734[c]
Ordinal by Ordinal	Spearman Correlation	.029	.084	.341	.734[c]
N of Valid Cases		140			

Symmetric Measures

		Exact Significance
Interval by Interval	Pearson's R	.864
Ordinal by Ordinal	Spearman Correlation	.864
N of Valid Cases		

a. Not assuming the null hypothesis.

b. Using the asymptotic standard error assuming the null hypothesis.

c. Based on normal approximation.

previously studied content related to ASD * group

Crosstab

			group		
			micro learning	traditional	Total
previously studied content related to ASD	no	Count	63	61	124
		% within previously studied content related to ASD	50.8%	49.2%	100.0%
		% within group	90.0%	87.1%	88.6%
		% of Total	45.0%	43.6%	88.6%
	yes	Count	7	9	16
		% within previously studied content related to ASD	43.8%	56.3%	100.0%
		% within group	10.0%	12.9%	11.4%
		% of Total	5.0%	6.4%	11.4%
Total		Count	70	70	140
		% within previously studied content related to ASD	50.0%	50.0%	100.0%
		% within group	100.0%	100.0%	100.0%
		% of Total	50.0%	50.0%	100.0%

Chi-Square Tests

	Value	df	Asymptotic Significance (2-sided)	Exact Sig. (2-sided)	Exact Sig. (1-sided)
Pearson Chi-Square	.282[a]	1	.595	.791	.396
Continuity Correction[b]	.071	1	.791		
Likelihood Ratio	.283	1	.595	.791	.396
Fisher's Exact Test				.791	.396
Linear-by-Linear Association	.280[c]	1	.597	.791	.396
N of Valid Cases	140				

Chi-Square Tests

	Point Probability
Pearson Chi-Square	
Continuity Correction[b]	
Likelihood Ratio	
Fisher's Exact Test	
Linear-by-Linear Association	.182
N of Valid Cases	

a. 0 cells (.0%) have expected count less than 5. The minimum expected count is 8.00.

b. Computed only for a 2x2 table

c. The standardized statistic is .529.

Symmetric Measures

		Value	Asymptotic Standard Error[a]	Approximate T[b]	Approximate Significance
Interval by Interval	Pearson's R	.045	.084	.528	.598[c]
Ordinal by Ordinal	Spearman Correlation	.045	.084	.528	.598[c]
N of Valid Cases		140			

Symmetric Measures

		Exact Significance
Interval by Interval	Pearson's R	.791
Ordinal by Ordinal	Spearman Correlation	.791
N of Valid Cases		

a. Not assuming the null hypothesis.

b. Using the asymptotic standard error assuming the null hypothesis.

c. Based on normal approximation.

family member or relative diagnosed with ASD * group

Crosstab

			group		
			micro learning	traditional	Total
family member or relative diagnosed with ASD	no	Count	66	64	130
		% within family member or relative diagnosed with ASD	50.8%	49.2%	100.0%
		% within group	94.3%	91.4%	92.9%
		% of Total	47.1%	45.7%	92.9%
	yes	Count	4	6	10
		% within family member or relative diagnosed with ASD	40.0%	60.0%	100.0%
		% within group	5.7%	8.6%	7.1%
		% of Total	2.9%	4.3%	7.1%
Total		Count	70	70	140
		% within family member or relative diagnosed with ASD	50.0%	50.0%	100.0%
		% within group	100.0%	100.0%	100.0%
		% of Total	50.0%	50.0%	100.0%

Chi-Square Tests

	Value	df	Asymptotic Significance (2-sided)	Exact Sig. (2-sided)	Exact Sig. (1-sided)	Point Probability
Pearson Chi-Square	.431[a]	1	.512	.745	.372	
Continuity Correction[b]	.108	1	.743			
Likelihood Ratio	.433	1	.510	.745	.372	
Fisher's Exact Test				.745	.372	
Linear-by-Linear Association	.428[c]	1	.513	.745	.372	.210
N of Valid Cases	140					

a. 0 cells (.0%) have expected count less than 5. The minimum expected count is 5.00.

b. Computed only for a 2x2 table

c. The standardized statistic is .654.

Symmetric Measures

		Value	Asymptotic Standard Error[a]	Approximate T[b]	Approximate Significance
Interval by Interval	Pearson's R	.055	.083	.653	.515[c]
Ordinal by Ordinal	Spearman Correlation	.055	.083	.653	.515[c]
N of Valid Cases		140			

Symmetric Measures

		Exact Significance
Interval by Interval	Pearson's R	.745
Ordinal by Ordinal	Spearman Correlation	.745
N of Valid Cases		

a. Not assuming the null hypothesis.

b. Using the asymptotic standard error assuming the null hypothesis.

c. Based on normal approximation.

attended any ASD-related training, workshop, or seminar * group

Crosstab

			group		
			micro learning	traditional	Total
attended any ASD-related training, workshop, or seminar	no	Count	62	64	126
		% within attended any ASD-related training, workshop, or seminar	49.2%	50.8%	100.0%
		% within group	88.6%	91.4%	90.0%
		% of Total	44.3%	45.7%	90.0%
	yes	Count	8	6	14
		% within attended any ASD-related training, workshop, or seminar	57.1%	42.9%	100.0%
		% within group	11.4%	8.6%	10.0%
		% of Total	5.7%	4.3%	10.0%
Total		Count	70	70	140
		% within attended any ASD-related training, workshop, or seminar	50.0%	50.0%	100.0%
		% within group	100.0%	100.0%	100.0%
		% of Total	50.0%	50.0%	100.0%

Chi-Square Tests

	Value	df	Asymptotic Significance (2-sided)	Exact Sig. (2-sided)	Exact Sig. (1-sided)	Point Probability
Pearson Chi-Square	.317[a]	1	.573	.779	.390	
Continuity Correction[b]	.079	1	.778			
Likelihood Ratio	.318	1	.573	.779	.390	
Fisher's Exact Test				.779	.390	
Linear-by-Linear Association	.315[c]	1	.575	.779	.390	.190
N of Valid Cases	140					

a. 0 cells (.0%) have expected count less than 5. The minimum expected count is 7.00.

b. Computed only for a 2x2 table

c. The standardized statistic is -.561.

Symmetric Measures

		Value	Asymptotic Standard Error[a]	Approximate T[b]	Approximate Significance
Interval by Interval	Pearson's R	-.048	.084	-.560	.576[c]
Ordinal by Ordinal	Spearman Correlation	-.048	.084	-.560	.576[c]
N of Valid Cases		140			

Symmetric Measures

		Exact Significance
Interval by Interval	Pearson's R	.779
Ordinal by Ordinal	Spearman Correlation	.779
N of Valid Cases		

a. Not assuming the null hypothesis.

b. Using the asymptotic standard error assuming the null hypothesis.

c. Based on normal approximation.

provided direct care or interaction with a person with ASD * group

Crosstab

			group		
			micro learning	traditional	Total
provided direct care or interaction with a person with ASD	no	Count	60	63	123
		% within provided direct care or interaction with a person with ASD	48.8%	51.2%	100.0%
		% within group	85.7%	90.0%	87.9%
		% of Total	42.9%	45.0%	87.9%
	yes	Count	10	7	17
		% within provided direct care or interaction with a person with ASD	58.8%	41.2%	100.0%
		% within group	14.3%	10.0%	12.1%
		% of Total	7.1%	5.0%	12.1%
Total		Count	70	70	140
		% within provided direct care or interaction with a person with ASD	50.0%	50.0%	100.0%
		% within group	100.0%	100.0%	100.0%
		% of Total	50.0%	50.0%	100.0%

Chi-Square Tests

	Value	df	Asymptotic Significance (2-sided)	Exact Sig. (2-sided)	Exact Sig. (1-sided)
Pearson Chi-Square	.603[a]	1	.438	.606	.303
Continuity Correction[b]	.268	1	.605		
Likelihood Ratio	.605	1	.437	.606	.303
Fisher's Exact Test				.606	.303
Linear-by-Linear Association	.598[c]	1	.439	.606	.303
N of Valid Cases	140				

Chi-Square Tests

	Point Probability
Pearson Chi-Square	
Continuity Correction[b]	
Likelihood Ratio	
Fisher's Exact Test	
Linear-by-Linear Association	.153
N of Valid Cases	

a. 0 cells (.0%) have expected count less than 5. The minimum expected count is 8.50.

b. Computed only for a 2x2 table

c. The standardized statistic is -.773.

Symmetric Measures

		Value	Asymptotic Standard Error[a]	Approximate T[b]	Approximate Significance
Interval by Interval	Pearson's R	-.066	.084	-.772	.441[c]
Ordinal by Ordinal	Spearman Correlation	-.066	.084	-.772	.441[c]
N of Valid Cases		140			

Symmetric Measures

		Exact Significance
Interval by Interval	Pearson's R	.606
Ordinal by Ordinal	Spearman Correlation	.606
N of Valid Cases		

a. Not assuming the null hypothesis.

b. Using the asymptotic standard error assuming the null hypothesis.

c. Based on normal approximation.

```
 USE ALL.
COMPUTE filter_$=(group = 0).
VARIABLE LABELS filter_$ 'group = 0 (FILTER)'.
VALUE LABELS filter_$ 0 'Not Selected' 1 'Selected'.
FORMATS filter_$ (f1.0).
FILTER BY filter_$.
EXECUTE.
DESCRIPTIVES VARIABLES=actual.age
  /STATISTICS=MEAN STDDEV MIN MAX.
```

Descriptives

Notes

Output Created		19-SEP-2026 09:17:26
Comments		
Input	Data	F:\\ \asd\ \major.revision. 9.2026 \ASD_Deidentified_Dataset.sav
	Active Dataset	DataSet1
	Filter	group = 0 (FILTER)
	Weight	<none>
	Split File	<none>
	N of Rows in Working Data File	70
Missing Value Handling	Definition of Missing	User defined missing values are treated as missing.
	Cases Used	All non-missing data are used.
Syntax		DESCRIPTIVES VARIABLES=actual.age /STATISTICS=MEAN STDDEV MIN MAX.
Resources	Processor Time	00:00:00.00
	Elapsed Time	00:00:00.00

Descriptive Statistics

	N	Minimum	Maximum	Mean	Std. Deviation
actual.age	70	19.00	45.00	23.8857	4.49974
Valid N (listwise)	70				

```
FREQUENCIES VARIABLES=q.1.pre q.1.post q.2.pre q.2.post q.3.pre q.3.post q.4.p
re q.4.post q.5.pre
    q.5.post q.6.pre q.6.post q.7.pre q.7.post q.8.pre q.8.post q.9.pre q.9.po
st q.10.pre q.10.post
    q.11.pre q.11.post q.12.pre q.12..post q.13.pre q.13.post q.14.pre q.14.po
st
  /ORDER=ANALYSIS.
```

Frequencies

Notes

Output Created		19-SEP-2026 09:17:52
Comments		
Input	Data	F:\\ \asd\ \major.revision.9.2026\ASD_Deidentified_Dataset.sav
	Active Dataset	DataSet1
	Filter	group = 0 (FILTER)
	Weight	<none>
	Split File	<none>
	N of Rows in Working Data File	70
Missing Value Handling	Definition of Missing	User-defined missing values are treated as missing.
	Cases Used	Statistics are based on all cases with valid data.
Syntax		FREQUENCIES VARIABLES=q.1.pre q.1.post q.2.pre q.2.post q.3.pre q.3.post q.4.pre q.4.post q.5.pre q.5.post q.6.pre q.6.post q.7.pre q.7.post q.8.pre q.8.post q.9.pre q.9.post q.10.pre q.10.post q.11.pre q.11.post q.12.pre q.12..post q.13.pre q.13.post q.14.pre q.14.post /ORDER=ANALYSIS.
Resources	Processor Time	00:00:00.00
	Elapsed Time	00:00:00.00

Statistics

		Children with ASD may show deficits in social reciprocity, such as difficulty with back-and-forth conversation.	q.1.post	Walking problems, such as difficulty climbing stairs, are a core feature of ASD	q.2.post	Deficits in nonverbal communication, such as limited eye contact, are common in ASD	q.3.post
N	Valid	70	70	70	70	70	70
	Missing	0	0	0	0	0	0

Statistics

		Difficulties in developing and maintaining relationships are characteristic of ASD	q.4.post	Repetitive behaviors, such as lining up toys, may be observed in children with ASD	q.5.post	Rigid adherence to routines, such as insisting on taking the same route to school, is common in ASD	q.6.post
N	Valid	70	70	70	70	70	70
	Missing	0	0	0	0	0	0

Statistics

		Children with ASD may display highly restricted or fixated interests, such as focusing on specific objects only.	q.7.post	Unusual responses to sensory input, such as covering ears in response to certain sounds, are associated with ASD	q.8.post	Delayed toilet training is a core diagnostic red flag of ASD	q.9.post
N	Valid	70	70	70	70	70	70
	Missing	0	0	0	0	0	0

Statistics

		Psychosocial interventions play an important role in the management of ASD	q.10.post	Pharmacological treatment may be used to manage associated symptoms of ASD	q.11.post	Dietary interventions alone are an evidence-based treatment for ASD	q.12..post
N	Valid	70	70	70	70	70	70
	Missing	0	0	0	0	0	0

Statistics

		Hyperbaric oxygen therapy is an evidence-based treatment for ASD	q.13.post	Behavioral, developmental, and multidisciplinary educational interventions are effective approaches for managing ASD	q.14.post
N	Valid	70	70	70	70
	Missing	0	0	0	0

Frequency Table

Children with ASD may show deficits in social reciprocity, such as difficulty with back-and-forth conversation.

		Frequency	Percent	Valid Percent	Cumulative Percent
Valid	incorrect	61	87.1	87.1	87.1
	correct	9	12.9	12.9	100.0
	Total	70	100.0	100.0	

q.1.post

		Frequency	Percent	Valid Percent	Cumulative Percent
Valid	incorrect	6	8.6	8.6	8.6
	correct	64	91.4	91.4	100.0
	Total	70	100.0	100.0	

Walking problems, such as difficulty climbing stairs, are a core feature of ASD

		Frequency	Percent	Valid Percent	Cumulative Percent
Valid	incorrect	62	88.6	88.6	88.6
	correct	8	11.4	11.4	100.0
	Total	70	100.0	100.0	

q.2.post

		Frequency	Percent	Valid Percent	Cumulative Percent
Valid	incorrect	4	5.7	5.7	5.7
	correct	66	94.3	94.3	100.0
	Total	70	100.0	100.0	

Deficits in nonverbal communication, such as limited eye contact, are common in ASD

		Frequency	Percent	Valid Percent	Cumulative Percent
Valid	incorrect	60	85.7	85.7	85.7
	correct	10	14.3	14.3	100.0
	Total	70	100.0	100.0	

q.3.post

		Frequency	Percent	Valid Percent	Cumulative Percent
Valid	incorrect	6	8.6	8.6	8.6
	correct	64	91.4	91.4	100.0
	Total	70	100.0	100.0	

Difficulties in developing and maintaining relationships are characteristic of ASD

		Frequency	Percent	Valid Percent	Cumulative Percent
Valid	incorrect	61	87.1	87.1	87.1
	correct	9	12.9	12.9	100.0
	Total	70	100.0	100.0	

q.4.post

		Frequency	Percent	Valid Percent	Cumulative Percent
Valid	incorrect	11	15.7	15.7	15.7
	correct	59	84.3	84.3	100.0
	Total	70	100.0	100.0	

Repetitive behaviors, such as lining up toys, may be observed in children with ASD

		Frequency	Percent	Valid Percent	Cumulative Percent
Valid	incorrect	61	87.1	87.1	87.1
	correct	9	12.9	12.9	100.0
	Total	70	100.0	100.0	

q.5.post

		Frequency	Percent	Valid Percent	Cumulative Percent
Valid	incorrect	12	17.1	17.1	17.1
	correct	58	82.9	82.9	100.0
	Total	70	100.0	100.0	

Rigid adherence to routines, such as insisting on taking the same route to school, is common in ASD

		Frequency	Percent	Valid Percent	Cumulative Percent
Valid	incorrect	59	84.3	84.3	84.3
	correct	11	15.7	15.7	100.0
	Total	70	100.0	100.0	

q.6.post

		Frequency	Percent	Valid Percent	Cumulative Percent
Valid	incorrect	4	5.7	5.7	5.7
	correct	66	94.3	94.3	100.0
	Total	70	100.0	100.0	

Children with ASD may display highly restricted or fixated interests, such as focusing on specific objects only.

		Frequency	Percent	Valid Percent	Cumulative Percent
Valid	incorrect	56	80.0	80.0	80.0
	correct	14	20.0	20.0	100.0
	Total	70	100.0	100.0	

q.7.post

		Frequency	Percent	Valid Percent	Cumulative Percent
Valid	incorrect	6	8.6	8.6	8.6
	correct	64	91.4	91.4	100.0
	Total	70	100.0	100.0	

Unusual responses to sensory input, such as covering ears in response to certain sounds, are associated with ASD

		Frequency	Percent	Valid Percent	Cumulative Percent
Valid	incorrect	58	82.9	82.9	82.9
	correct	12	17.1	17.1	100.0
	Total	70	100.0	100.0	

q.8.post

		Frequency	Percent	Valid Percent	Cumulative Percent
Valid	incorrect	8	11.4	11.4	11.4
	correct	62	88.6	88.6	100.0
	Total	70	100.0	100.0	

Delayed toilet training is a core diagnostic red flag of ASD

		Frequency	Percent	Valid Percent	Cumulative Percent
Valid	incorrect	64	91.4	91.4	91.4
	correct	6	8.6	8.6	100.0
	Total	70	100.0	100.0	

q.9.post

		Frequency	Percent	Valid Percent	Cumulative Percent
Valid	incorrect	10	14.3	14.3	14.3
	correct	60	85.7	85.7	100.0
	Total	70	100.0	100.0	

Psychosocial interventions play an important role in the management of ASD

		Frequency	Percent	Valid Percent	Cumulative Percent
Valid	incorrect	57	81.4	81.4	81.4
	correct	13	18.6	18.6	100.0
	Total	70	100.0	100.0	

q.10.post

		Frequency	Percent	Valid Percent	Cumulative Percent
Valid	incorrect	5	7.1	7.1	7.1
	correct	65	92.9	92.9	100.0
	Total	70	100.0	100.0	

Pharmacological treatment may be used to manage associated symptoms of ASD

		Frequency	Percent	Valid Percent	Cumulative Percent
Valid	incorrect	62	88.6	88.6	88.6
	correct	8	11.4	11.4	100.0
	Total	70	100.0	100.0	

q.11.post

		Frequency	Percent	Valid Percent	Cumulative Percent
Valid	incorrect	3	4.3	4.3	4.3
	correct	67	95.7	95.7	100.0
	Total	70	100.0	100.0	

Dietary interventions alone are an evidence-based treatment for ASD

		Frequency	Percent	Valid Percent	Cumulative Percent
Valid	incorrect	63	90.0	90.0	90.0
	correct	7	10.0	10.0	100.0
	Total	70	100.0	100.0	

q.12..post

		Frequency	Percent	Valid Percent	Cumulative Percent
Valid	incorrect	11	15.7	15.7	15.7
	correct	59	84.3	84.3	100.0
	Total	70	100.0	100.0	

Hyperbaric oxygen therapy is an evidence-based treatment for ASD

		Frequency	Percent	Valid Percent	Cumulative Percent
Valid	incorrect	66	94.3	94.3	94.3
	correct	4	5.7	5.7	100.0
	Total	70	100.0	100.0	

q.13.post

		Frequency	Percent	Valid Percent	Cumulative Percent
Valid	incorrect	7	10.0	10.0	10.0
	correct	63	90.0	90.0	100.0
	Total	70	100.0	100.0	

Behavioral, developmental, and multidisciplinary educational interventions are effective approaches for managing ASD

		Frequency	Percent	Valid Percent	Cumulative Percent
Valid	incorrect	61	87.1	87.1	87.1
	correct	9	12.9	12.9	100.0
	Total	70	100.0	100.0	

q.14.post

		Frequency	Percent	Valid Percent	Cumulative Percent
Valid	incorrect	12	17.1	17.1	17.1
	correct	58	82.9	82.9	100.0
	Total	70	100.0	100.0	

```
CROSSTABS
 /TABLES=q.1.pre BY q.1.post
 /CELLS=COUNT ROW COLUMN
 /STATISTICS=MCNEMAR.
```

Crosstabs

Notes

Output Created		19-SEP-2026 09:18:07
Comments		
Input	Data	F:\\ \asd\ \major.revision.9.2026\ASD_Deidentified_Dataset.sav
	Active Dataset	DataSet1
	Filter	group = 0 (FILTER)
	Weight	<none>
	Split File	<none>
	N of Rows in Working Data File	70
Missing Value Handling	Definition of Missing	User-defined missing values are treated as missing.
	Cases Used	Statistics for each table are based on all the cases with valid data in the specified range(s) for all variables in each table.
Syntax		CROSSTABS /TABLES=q.1.pre BY q.1.post /CELLS=COUNT ROW COLUMN /STATISTICS=MCNEMAR...
Resources	Processor Time	00:00:00.00
	Elapsed Time	00:00:00.00
	Dimensions Requested	2
	Cells Available	524245

Case Processing Summary

	Cases					
	Valid		Missing		Total	
	N	Percent	N	Percent	N	Percent
Children with ASD may show deficits in social reciprocity, such as difficulty with back-and-forth conversation. * q.1.post	70	100.0%	0	0.0%	70	100.0%

Children with ASD may show deficits in social reciprocity, such as difficulty with back-and-forth conversation. * q.1.post Crosstabulation

			q.1.post		Total
			incorrect	correct	
Children with ASD may show deficits in social reciprocity, such as difficulty with back-and-forth conversation.	incorrect	Count	5	56	61
		% within Children with ASD may show deficits in social reciprocity, such as difficulty with back-and-forth conversation.	8.2%	91.8%	100.0%
		% within q.1.post	83.3%	87.5%	87.1%
	correct	Count	1	8	9
		% within Children with ASD may show deficits in social reciprocity, such as difficulty with back-and-forth conversation.	11.1%	88.9%	100.0%
		% within q.1.post	16.7%	12.5%	12.9%
Total		Count	6	64	70
		% within Children with ASD may show deficits in social reciprocity, such as difficulty with back-and-forth conversation.	8.6%	91.4%	100.0%
		% within q.1.post	100.0%	100.0%	100.0%

Chi-Square Tests

	Value	Exact Sig. (2-sided)
McNemar Test		.000[a]
N of Valid Cases	70	

a. Binomial distribution used.

```
CROSSTABS
 /TABLES=q.2.pre BY q.2.post
 /CELLS=COUNT ROW COLUMN
 /STATISTICS=MCNEMAR.
```

Crosstabs

Notes

Output Created		19-SEP-2026 09:18:07
Comments		
Input	Data	F:\\ \asd\ \major.revision. 9.2026 \ASD_Deidentified_Dataset.sav
	Active Dataset	DataSet1
	Filter	group = 0 (FILTER)
	Weight	<none>
	Split File	<none>
	N of Rows in Working Data File	70
Missing Value Handling	Definition of Missing	User-defined missing values are treated as missing.
	Cases Used	Statistics for each table are based on all the cases with valid data in the specified range(s) for all variables in each table.
Syntax		CROSSTABS /TABLES=q.2.pre BY q.2.post /CELLS=COUNT ROW COLUMN /STATISTICS=MCNEMAR...
Resources	Processor Time	00:00:00.00
	Elapsed Time	00:00:00.00
	Dimensions Requested	2
	Cells Available	524245

Case Processing Summary

	Cases					
	Valid		Missing		Total	
	N	Percent	N	Percent	N	Percent
Walking problems, such as difficulty climbing stairs, are a core feature of ASD * q.2.post	70	100.0%	0	0.0%	70	100.0%

Walking problems, such as difficulty climbing stairs, are a core feature of ASD * q.2.post Crosstabulation

			q.2.post		
			incorrect	correct	Total
Walking problems, such as difficulty climbing stairs, are a core feature of ASD	incorrect	Count	4	58	62
		% within Walking problems, such as difficulty climbing stairs, are a core feature of ASD	6.5%	93.5%	100.0%
		% within q.2.post	100.0%	87.9%	88.6%
	correct	Count	0	8	8
		% within Walking problems, such as difficulty climbing stairs, are a core feature of ASD	0.0%	100.0%	100.0%
		% within q.2.post	0.0%	12.1%	11.4%
Total		Count	4	66	70
		% within Walking problems, such as difficulty climbing stairs, are a core feature of ASD	5.7%	94.3%	100.0%
		% within q.2.post	100.0%	100.0%	100.0%

Chi-Square Tests

	Value	Exact Sig. (2-sided)
McNemar Test		.000[a]
N of Valid Cases	70	

a. Binomial distribution used.

```
CROSSTABS
 /TABLES=q.3.pre BY q.3.post
 /CELLS=COUNT ROW COLUMN
 /STATISTICS=MCNEMAR.
```

Crosstabs

Notes

Output Created		19-SEP-2026 09:18:07
Comments		
Input	Data	F:\\ \asd\ \major.revision.9.2026\ASD_Deidentified_Dataset.sav
	Active Dataset	DataSet1
	Filter	group = 0 (FILTER)
	Weight	<none>
	Split File	<none>
	N of Rows in Working Data File	70
Missing Value Handling	Definition of Missing	User-defined missing values are treated as missing.
	Cases Used	Statistics for each table are based on all the cases with valid data in the specified range(s) for all variables in each table.
Syntax		CROSSTABS /TABLES=q.3.pre BY q.3.post /CELLS=COUNT ROW COLUMN /STATISTICS=MCNEMAR...
Resources	Processor Time	00:00:00.00
	Elapsed Time	00:00:00.00
	Dimensions Requested	2
	Cells Available	524245

Case Processing Summary

	Cases					
	Valid		Missing		Total	
	N	Percent	N	Percent	N	Percent
Deficits in nonverbal communication, such as limited eye contact, are common in ASD * q.3.post	70	100.0%	0	0.0%	70	100.0%

Deficits in nonverbal communication, such as limited eye contact, are common in ASD * q.3.post Crosstabulation

			q.3.post		
			incorrect	correct	Total
Deficits in nonverbal communication, such as limited eye contact, are common in ASD	incorrect	Count	5	55	60
		% within Deficits in nonverbal communication, such as limited eye contact, are common in ASD	8.3%	91.7%	100.0%
		% within q.3.post	83.3%	85.9%	85.7%
	correct	Count	1	9	10
		% within Deficits in nonverbal communication, such as limited eye contact, are common in ASD	10.0%	90.0%	100.0%
		% within q.3.post	16.7%	14.1%	14.3%
Total		Count	6	64	70
		% within Deficits in nonverbal communication, such as limited eye contact, are common in ASD	8.6%	91.4%	100.0%
		% within q.3.post	100.0%	100.0%	100.0%

Chi-Square Tests

	Value	Exact Sig. (2-sided)
McNemar Test		.000[a]
N of Valid Cases	70	

a. Binomial distribution used.

```
CROSSTABS
 /TABLES=q.4.pre BY q.4.post
 /CELLS=COUNT ROW COLUMN
 /STATISTICS=MCNEMAR.
```

Crosstabs

Notes

Output Created		19-SEP-2026 09:18:07
Comments		
Input	Data	F:\\ \asd\ \major.revision. 9.2026 \ASD_Deidentified_Dataset.sav
	Active Dataset	DataSet1
	Filter	group = 0 (FILTER)
	Weight	<none>
	Split File	<none>
	N of Rows in Working Data File	70
Missing Value Handling	Definition of Missing	User-defined missing values are treated as missing.
	Cases Used	Statistics for each table are based on all the cases with valid data in the specified range(s) for all variables in each table.
Syntax		CROSSTABS /TABLES=q.4.pre BY q.4.post /CELLS=COUNT ROW COLUMN /STATISTICS=MCNEMAR...
Resources	Processor Time	00:00:00.02
	Elapsed Time	00:00:00.02
	Dimensions Requested	2
	Cells Available	524245

Case Processing Summary

	Cases					
	Valid		Missing		Total	
	N	Percent	N	Percent	N	Percent
Difficulties in developing and maintaining relationships are characteristic of ASD * q.4.post	70	100.0%	0	0.0%	70	100.0%

Difficulties in developing and maintaining relationships are characteristic of ASD * q.4.post Crosstabulation

			q.4.post		
			incorrect	correct	Total
Difficulties in developing and maintaining relationships are characteristic of ASD	incorrect	Count	4	57	61
		% within Difficulties in developing and maintaining relationships are characteristic of ASD	6.6%	93.4%	100.0%
		% within q.4.post	36.4%	96.6%	87.1%
	correct	Count	7	2	9
		% within Difficulties in developing and maintaining relationships are characteristic of ASD	77.8%	22.2%	100.0%
		% within q.4.post	63.6%	3.4%	12.9%
Total		Count	11	59	70
		% within Difficulties in developing and maintaining relationships are characteristic of ASD	15.7%	84.3%	100.0%
		% within q.4.post	100.0%	100.0%	100.0%

Chi-Square Tests

	Value	Exact Sig. (2-sided)
McNemar Test		.000[a]
N of Valid Cases	70	

a. Binomial distribution used.

```
CROSSTABS
 /TABLES=q.5.pre BY q.5.post
 /CELLS=COUNT ROW COLUMN
 /STATISTICS=MCNEMAR.
```

Crosstabs

Notes

Output Created		19-SEP-2026 09:18:07
Comments		
Input	Data	F:\\ \asd\ \major.revision.9.2026\ASD_Deidentified_Dataset.sav
	Active Dataset	DataSet1
	Filter	group = 0 (FILTER)
	Weight	<none>
	Split File	<none>
	N of Rows in Working Data File	70
Missing Value Handling	Definition of Missing	User-defined missing values are treated as missing.
	Cases Used	Statistics for each table are based on all the cases with valid data in the specified range(s) for all variables in each table.
Syntax		CROSSTABS<br>/TABLES=q.5.pre BY q.5.post<br>/CELLS=COUNT ROW COLUMN<br><br>/STATISTICS=MCNEMAR...
Resources	Processor Time	00:00:00.02
	Elapsed Time	00:00:00.02
	Dimensions Requested	2
	Cells Available	524245

Case Processing Summary

	Cases					
	Valid		Missing		Total	
	N	Percent	N	Percent	N	Percent
Repetitive behaviors, such as lining up toys, may be observed in children with ASD * q.5.post	70	100.0%	0	0.0%	70	100.0%

Repetitive behaviors, such as lining up toys, may be observed in children with ASD * q.5.post Crosstabulation

			q.5.post		
			incorrect	correct	Total
Repetitive behaviors, such as lining up toys, may be observed in children with ASD	incorrect	Count	9	52	61
		% within Repetitive behaviors, such as lining up toys, may be observed in children with ASD	14.8%	85.2%	100.0%
		% within q.5.post	75.0%	89.7%	87.1%
	correct	Count	3	6	9
		% within Repetitive behaviors, such as lining up toys, may be observed in children with ASD	33.3%	66.7%	100.0%
		% within q.5.post	25.0%	10.3%	12.9%
Total		Count	12	58	70
		% within Repetitive behaviors, such as lining up toys, may be observed in children with ASD	17.1%	82.9%	100.0%
		% within q.5.post	100.0%	100.0%	100.0%

Chi-Square Tests

	Value	Exact Sig. (2-sided)
McNemar Test		.000[a]
N of Valid Cases	70	

a. Binomial distribution used.

```
CROSSTABS
 /TABLES=q.6.pre BY q.6.post
 /CELLS=COUNT ROW COLUMN
 /STATISTICS=MCNEMAR.
```

Crosstabs

Notes

Output Created		19-SEP-2026 09:18:07
Comments		
Input	Data	F:\\ \asd\ \major.revision.9.2026\ASD_Deidentified_Dataset.sav
	Active Dataset	DataSet1
	Filter	group = 0 (FILTER)
	Weight	<none>
	Split File	<none>
	N of Rows in Working Data File	70
Missing Value Handling	Definition of Missing	User-defined missing values are treated as missing.
	Cases Used	Statistics for each table are based on all the cases with valid data in the specified range(s) for all variables in each table.
Syntax		CROSSTABS /TABLES=q.6.pre BY q.6.post /CELLS=COUNT ROW COLUMN /STATISTICS=MCNEMAR...
Resources	Processor Time	00:00:00.02
	Elapsed Time	00:00:00.02
	Dimensions Requested	2
	Cells Available	524245

Case Processing Summary

	Cases					
	Valid		Missing		Total	
	N	Percent	N	Percent	N	Percent
Rigid adherence to routines, such as insisting on taking the same route to school, is common in ASD * q.6.post	70	100.0%	0	0.0%	70	100.0%

Rigid adherence to routines, such as insisting on taking the same route to school, is common in ASD * q.6.post Crosstabulation

			q.6.post		
			incorrect	correct	Total
Rigid adherence to routines, such as insisting on taking the same route to school, is common in ASD	incorrect	Count	3	56	59
		% within Rigid adherence to routines, such as insisting on taking the same route to school, is common in ASD	5.1%	94.9%	100.0%
		% within q.6.post	75.0%	84.8%	84.3%
	correct	Count	1	10	11
		% within Rigid adherence to routines, such as insisting on taking the same route to school, is common in ASD	9.1%	90.9%	100.0%
		% within q.6.post	25.0%	15.2%	15.7%
Total		Count	4	66	70
		% within Rigid adherence to routines, such as insisting on taking the same route to school, is common in ASD	5.7%	94.3%	100.0%
		% within q.6.post	100.0%	100.0%	100.0%

Chi-Square Tests

	Value	Exact Sig. (2-sided)
McNemar Test		.000[a]
N of Valid Cases	70	

a. Binomial distribution used.

```
CROSSTABS
  /TABLES=q.7.pre BY q.7.post
  /CELLS=COUNT ROW COLUMN
  /STATISTICS=MCNEMAR.
```

Crosstabs

Notes

Output Created		19-SEP-2026 09:18:07
Comments		
Input	Data	F:\\ \asd\ \major.revision. 9.2026 \ASD_Deidentified_Dataset.sav
	Active Dataset	DataSet1
	Filter	group = 0 (FILTER)
	Weight	<none>
	Split File	<none>
	N of Rows in Working Data File	70
Missing Value Handling	Definition of Missing	User-defined missing values are treated as missing.
	Cases Used	Statistics for each table are based on all the cases with valid data in the specified range(s) for all variables in each table.
Syntax		CROSSTABS /TABLES=q.7.pre BY q.7.post /CELLS=COUNT ROW COLUMN /STATISTICS=MCNEMAR...
Resources	Processor Time	00:00:00.00
	Elapsed Time	00:00:00.00
	Dimensions Requested	2
	Cells Available	524245

Case Processing Summary

	Cases					
	Valid		Missing		Total	
	N	Percent	N	Percent	N	Percent
Children with ASD may display highly restricted or fixated interests, such as focusing on specific objects only. * q.7.post	70	100.0%	0	0.0%	70	100.0%

Children with ASD may display highly restricted or fixated interests, such as focusing on specific objects only. * q.7.post Crosstabulation

			q.7.post		
			incorrect	correct	Total
Children with ASD may display highly restricted or fixated interests, such as focusing on specific objects only.	incorrect	Count	3	53	56
		% within Children with ASD may display highly restricted or fixated interests, such as focusing on specific objects only.	5.4%	94.6%	100.0%
		% within q.7.post	50.0%	82.8%	80.0%
	correct	Count	3	11	14
		% within Children with ASD may display highly restricted or fixated interests, such as focusing on specific objects only.	21.4%	78.6%	100.0%
		% within q.7.post	50.0%	17.2%	20.0%
Total		Count	6	64	70
		% within Children with ASD may display highly restricted or fixated interests, such as focusing on specific objects only.	8.6%	91.4%	100.0%
		% within q.7.post	100.0%	100.0%	100.0%

Chi-Square Tests

	Value	Exact Sig. (2-sided)
McNemar Test		.000[a]
N of Valid Cases	70	

a. Binomial distribution used.

```
CROSSTABS
 /TABLES=q.8.pre BY q.8.post
 /CELLS=COUNT ROW COLUMN
 /STATISTICS=MCNEMAR.
```

Crosstabs

Notes

Output Created		19-SEP-2026 09:18:07
Comments		
Input	Data	F:\\ \asd\ \major.revision. 9.2026 \ASD_Deidentified_Dataset.sav
	Active Dataset	DataSet1
	Filter	group = 0 (FILTER)
	Weight	<none>
	Split File	<none>
	N of Rows in Working Data File	70
Missing Value Handling	Definition of Missing	User-defined missing values are treated as missing.
	Cases Used	Statistics for each table are based on all the cases with valid data in the specified range(s) for all variables in each table.
Syntax		CROSSTABS /TABLES=q.8.pre BY q.8.post /CELLS=COUNT ROW COLUMN /STATISTICS=MCNEMAR...
Resources	Processor Time	00:00:00.02
	Elapsed Time	00:00:00.01
	Dimensions Requested	2
	Cells Available	524245

Case Processing Summary

	Cases					
	Valid		Missing		Total	
	N	Percent	N	Percent	N	Percent
Unusual responses to sensory input, such as covering ears in response to certain sounds, are associated with ASD * q.8.post	70	100.0%	0	0.0%	70	100.0%

Unusual responses to sensory input, such as covering ears in response to certain sounds, are associated with ASD * q.8.post Crosstabulation

			q.8.post		
			incorrect	correct	Total
Unusual responses to sensory input, such as covering ears in response to certain sounds, are associated with ASD	incorrect	Count	4	54	58
		% within Unusual responses to sensory input, such as covering ears in response to certain sounds, are associated with ASD	6.9%	93.1%	100.0%
		% within q.8.post	50.0%	87.1%	82.9%
	correct	Count	4	8	12
		% within Unusual responses to sensory input, such as covering ears in response to certain sounds, are associated with ASD	33.3%	66.7%	100.0%
		% within q.8.post	50.0%	12.9%	17.1%
Total		Count	8	62	70
		% within Unusual responses to sensory input, such as covering ears in response to certain sounds, are associated with ASD	11.4%	88.6%	100.0%
		% within q.8.post	100.0%	100.0%	100.0%

Chi-Square Tests

	Value	Exact Sig. (2-sided)
McNemar Test		.000[a]
N of Valid Cases	70	

a. Binomial distribution used.

```
CROSSTABS
 /TABLES=q.9.pre BY q.9.post
 /CELLS=COUNT ROW COLUMN
 /STATISTICS=MCNEMAR.
```

Crosstabs

Notes

Output Created		19-SEP-2026 09:18:07
Comments		
Input	Data	F:\\ \asd\ \major.revision.9.2026\ASD_Deidentified_Dataset.sav
	Active Dataset	DataSet1
	Filter	group = 0 (FILTER)
	Weight	<none>
	Split File	<none>
	N of Rows in Working Data File	70
Missing Value Handling	Definition of Missing	User-defined missing values are treated as missing.
	Cases Used	Statistics for each table are based on all the cases with valid data in the specified range(s) for all variables in each table.

Notes

Syntax		CROSSTABS<br>/TABLES=q.9.pre BY q.9.post<br>/CELLS=COUNT ROW COLUMN<br><br>/STATISTICS=MCNEMAR...
Resources	Processor Time	00:00:00.00
	Elapsed Time	00:00:00.02
	Dimensions Requested	2
	Cells Available	524245

Case Processing Summary

	Cases					
	Valid		Missing		Total	
	N	Percent	N	Percent	N	Percent
Delayed toilet training is a core diagnostic red flag of ASD * q.9.post	70	100.0%	0	0.0%	70	100.0%

Delayed toilet training is a core diagnostic red flag of ASD * q.9.post Crosstabulation

			q.9.post		
			incorrect	correct	Total
Delayed toilet training is a core diagnostic red flag of ASD	incorrect	Count	9	55	64
		% within Delayed toilet training is a core diagnostic red flag of ASD	14.1%	85.9%	100.0%
		% within q.9.post	90.0%	91.7%	91.4%
	correct	Count	1	5	6
		% within Delayed toilet training is a core diagnostic red flag of ASD	16.7%	83.3%	100.0%
		% within q.9.post	10.0%	8.3%	8.6%
Total		Count	10	60	70
		% within Delayed toilet training is a core diagnostic red flag of ASD	14.3%	85.7%	100.0%
		% within q.9.post	100.0%	100.0%	100.0%

Chi-Square Tests

	Value	Exact Sig. (2-sided)
McNemar Test		.000[a]
N of Valid Cases	70	

a. Binomial distribution used.

```
CROSSTABS
 /TABLES=q.10.pre BY q.10.post
 /CELLS=COUNT ROW COLUMN
 /STATISTICS=MCNEMAR.
```

Crosstabs

Notes

Output Created		19-SEP-2026 09:18:07
Comments		
Input	Data	F:\\ \asd\ \major.revision.9.2026\ASD_Deidentified_Dataset.sav
	Active Dataset	DataSet1
	Filter	group = 0 (FILTER)
	Weight	<none>
	Split File	<none>
	N of Rows in Working Data File	70
Missing Value Handling	Definition of Missing	User-defined missing values are treated as missing.
	Cases Used	Statistics for each table are based on all the cases with valid data in the specified range(s) for all variables in each table.

Notes

Syntax		CROSSTABS<br>/TABLES=q.10.pre BY q.10.post<br>/CELLS=COUNT ROW COLUMN<br><br>/STATISTICS=MCNEMAR...
Resources	Processor Time	00:00:00.02
	Elapsed Time	00:00:00.02
	Dimensions Requested	2
	Cells Available	524245

Case Processing Summary

	Cases					
	Valid		Missing		Total	
	N	Percent	N	Percent	N	Percent
Psychosocial interventions play an important role in the management of ASD * q.10.post	70	100.0%	0	0.0%	70	100.0%

Psychosocial interventions play an important role in the management of ASD * q.10.post Crosstabulation

			q.10.post		
			incorrect	correct	Total
Psychosocial interventions play an important role in the management of ASD	incorrect	Count	4	53	57
		% within Psychosocial interventions play an important role in the management of ASD	7.0%	93.0%	100.0%
		% within q.10.post	80.0%	81.5%	81.4%
	correct	Count	1	12	13
		% within Psychosocial interventions play an important role in the management of ASD	7.7%	92.3%	100.0%
		% within q.10.post	20.0%	18.5%	18.6%
Total		Count	5	65	70
		% within Psychosocial interventions play an important role in the management of ASD	7.1%	92.9%	100.0%
		% within q.10.post	100.0%	100.0%	100.0%

Chi-Square Tests

	Value	Exact Sig. (2-sided)
McNemar Test		.000[a]
N of Valid Cases	70	

a. Binomial distribution used.

```
CROSSTABS
 /TABLES=q.11.pre BY q.11.post
 /CELLS=COUNT ROW COLUMN
 /STATISTICS=MCNEMAR.
```

Crosstabs

Notes

Output Created		19-SEP-2026 09:18:07
Comments		
Input	Data	F:\\ \asd\ \major.revision.9.2026\ASD_Deidentified_Dataset.sav
	Active Dataset	DataSet1
	Filter	group = 0 (FILTER)
	Weight	<none>
	Split File	<none>
	N of Rows in Working Data File	70
Missing Value Handling	Definition of Missing	User-defined missing values are treated as missing.
	Cases Used	Statistics for each table are based on all the cases with valid data in the specified range(s) for all variables in each table.
Syntax		CROSSTABS /TABLES=q.11.pre BY q.11.post /CELLS=COUNT ROW COLUMN /STATISTICS=MCNEMAR...
Resources	Processor Time	00:00:00.02
	Elapsed Time	00:00:00.02
	Dimensions Requested	2
	Cells Available	524245

Case Processing Summary

	Cases					
	Valid		Missing		Total	
	N	Percent	N	Percent	N	Percent
Pharmacological treatment may be used to manage associated symptoms of ASD * q.11.post	70	100.0%	0	0.0%	70	100.0%

Pharmacological treatment may be used to manage associated symptoms of ASD * q.11.post Crosstabulation

			q.11.post		
			incorrect	correct	Total
Pharmacological treatment may be used to manage associated symptoms of ASD	incorrect	Count	1	61	62
		% within Pharmacological treatment may be used to manage associated symptoms of ASD	1.6%	98.4%	100.0%
		% within q.11.post	33.3%	91.0%	88.6%
	correct	Count	2	6	8
		% within Pharmacological treatment may be used to manage associated symptoms of ASD	25.0%	75.0%	100.0%
		% within q.11.post	66.7%	9.0%	11.4%
Total		Count	3	67	70
		% within Pharmacological treatment may be used to manage associated symptoms of ASD	4.3%	95.7%	100.0%
		% within q.11.post	100.0%	100.0%	100.0%

Chi-Square Tests

	Value	Exact Sig. (2-sided)
McNemar Test		.000[a]
N of Valid Cases	70	

a. Binomial distribution used.

```
CROSSTABS
 /TABLES=q.12.pre BY q.12.post
 /CELLS=COUNT ROW COLUMN
 /STATISTICS=MCNEMAR.
```

Crosstabs

Notes

Output Created		19-SEP-2026 09:18:07
Comments		
Input	Data	F:\\ \asd\ \major.revision. 9.2026 \ASD_Deidentified_Dataset.sav
	Active Dataset	DataSet1
	Filter	group = 0 (FILTER)
	Weight	<none>
	Split File	<none>
	N of Rows in Working Data File	70
Missing Value Handling	Definition of Missing	User-defined missing values are treated as missing.
	Cases Used	Statistics for each table are based on all the cases with valid data in the specified range(s) for all variables in each table.
Syntax		CROSSTABS /TABLES=q.12.pre BY q.12.post /CELLS=COUNT ROW COLUMN /STATISTICS=MCNEMAR...
Resources	Processor Time	00:00:00.00
	Elapsed Time	00:00:00.00

Warnings

Text: q.12.post Command: CROSSTABS
An undefined variable name, or a scratch or system variable was specified in a variable list which accepts only standard variables. Check spelling and verify the existence of this variable.
Execution of this command stops.

```
CROSSTABS
 /TABLES=q.13.pre BY q.13.post
```

```
/CELLS=COUNT ROW COLUMN
/STATISTICS=MCNEMAR.
```

Crosstabs

Notes

Output Created		19-SEP-2026 09:18:07
Comments		
Input	Data	F:\\ \asd\ \major.revision.<br>9.2026<br>\ASD_Deidentified_Dataset.sav
	Active Dataset	DataSet1
	Filter	group = 0 (FILTER)
	Weight	<none>
	Split File	<none>
	N of Rows in Working Data File	70
Missing Value Handling	Definition of Missing	User-defined missing values are treated as missing.
	Cases Used	Statistics for each table are based on all the cases with valid data in the specified range(s) for all variables in each table.
Syntax		CROSSTABS<br>/TABLES=q.13.pre BY q.13.post<br>/CELLS=COUNT ROW COLUMN<br><br>/STATISTICS=MCNEMAR...
Resources	Processor Time	00:00:00.02
	Elapsed Time	00:00:00.02
	Dimensions Requested	2
	Cells Available	524245

Case Processing Summary

	Cases					
	Valid		Missing		Total	
	N	Percent	N	Percent	N	Percent
Hyperbaric oxygen therapy is an evidence-based treatment for ASD * q.13.post	70	100.0%	0	0.0%	70	100.0%

Hyperbaric oxygen therapy is an evidence-based treatment for ASD * q.13.post Crosstabulation

			q.13.post		
			incorrect	correct	Total
Hyperbaric oxygen therapy is an evidence-based treatment for ASD	incorrect	Count	7	59	66
		% within Hyperbaric oxygen therapy is an evidence-based treatment for ASD	10.6%	89.4%	100.0%
		% within q.13.post	100.0%	93.7%	94.3%
	correct	Count	0	4	4
		% within Hyperbaric oxygen therapy is an evidence-based treatment for ASD	0.0%	100.0%	100.0%
		% within q.13.post	0.0%	6.3%	5.7%
Total		Count	7	63	70
		% within Hyperbaric oxygen therapy is an evidence-based treatment for ASD	10.0%	90.0%	100.0%
		% within q.13.post	100.0%	100.0%	100.0%

Chi-Square Tests

	Value	Exact Sig. (2-sided)
McNemar Test		.000[a]
N of Valid Cases	70	

a. Binomial distribution used.

```
CROSSTABS
 /TABLES=q.14.pre BY q.14.post
 /CELLS=COUNT ROW COLUMN
 /STATISTICS=MCNEMAR.
```

Crosstabs

Notes

Output Created		19-SEP-2026 09:18:07
Comments		
Input	Data	F:\\ \asd\ \major.revision.9.2026\ASD_Deidentified_Dataset.sav
	Active Dataset	DataSet1
	Filter	group = 0 (FILTER)
	Weight	<none>
	Split File	<none>
	N of Rows in Working Data File	70
Missing Value Handling	Definition of Missing	User-defined missing values are treated as missing.
	Cases Used	Statistics for each table are based on all the cases with valid data in the specified range(s) for all variables in each table.
Syntax		CROSSTABS /TABLES=q.14.pre BY q.14.post /CELLS=COUNT ROW COLUMN /STATISTICS=MCNEMAR...
Resources	Processor Time	00:00:00.02
	Elapsed Time	00:00:00.02
	Dimensions Requested	2
	Cells Available	524245

Case Processing Summary

	Cases					
	Valid		Missing		Total	
	N	Percent	N	Percent	N	Percent
Behavioral, developmental, and multidisciplinary educational interventions are effective approaches for managing ASD * q.14.post	70	100.0%	0	0.0%	70	100.0%

Behavioral, developmental, and multidisciplinary educational interventions are effective approaches for managing ASD * q.14.post Crosstabulation

			q.14.post		
			incorrect	correct	Total
Behavioral, developmental, and multidisciplinary educational interventions are effective approaches for managing ASD	incorrect	Count	9	52	61
		% within Behavioral, developmental, and multidisciplinary educational interventions are effective approaches for managing ASD	14.8%	85.2%	100.0%
		% within q.14.post	75.0%	89.7%	87.1%
	correct	Count	3	6	9
		% within Behavioral, developmental, and multidisciplinary educational interventions are effective approaches for managing ASD	33.3%	66.7%	100.0%
		% within q.14.post	25.0%	10.3%	12.9%
Total		Count	12	58	70
		% within Behavioral, developmental, and multidisciplinary educational interventions are effective approaches for managing ASD	17.1%	82.9%	100.0%
		% within q.14.post	100.0%	100.0%	100.0%

Chi-Square Tests

	Value	Exact Sig. (2-sided)
McNemar Test		.000[a]
N of Valid Cases	70	

a. Binomial distribution used.

Crosstabs

Notes

Output Created		19-SEP-2026 09:20:27
Comments		
Input	Data	F:\\ \asd\ \major.revision.9.2026\ASD_Deidentified_Dataset.sav
	Active Dataset	DataSet1
	Filter	group = 0 (FILTER)
	Weight	<none>
	Split File	<none>
	N of Rows in Working Data File	70
Missing Value Handling	Definition of Missing	User-defined missing values are treated as missing.
	Cases Used	Statistics for each table are based on all the cases with valid data in the specified range(s) for all variables in each table.
Syntax		CROSSTABS /TABLES=q.12.pre BY q.12.post /CELLS=COUNT ROW COLUMN /STATISTICS=MCNEMAR...
Resources	Processor Time	00:00:00.00
	Elapsed Time	00:00:00.00
	Dimensions Requested	2
	Cells Available	524245

Case Processing Summary

	Cases					
	Valid		Missing		Total	
	N	Percent	N	Percent	N	Percent
Dietary interventions alone are an evidence-based treatment for ASD * q.12.post	70	100.0%	0	0.0%	70	100.0%

Dietary interventions alone are an evidence-based treatment for ASD * q.12.post Crosstabulation

			q.12.post		
			incorrect	correct	Total
Dietary interventions alone are an evidence-based treatment for ASD	incorrect	Count	9	54	63
		% within Dietary interventions alone are an evidence-based treatment for ASD	14.3%	85.7%	100.0%
		% within q.12.post	81.8%	91.5%	90.0%
	correct	Count	2	5	7
		% within Dietary interventions alone are an evidence-based treatment for ASD	28.6%	71.4%	100.0%
		% within q.12.post	18.2%	8.5%	10.0%
Total		Count	11	59	70
		% within Dietary interventions alone are an evidence-based treatment for ASD	15.7%	84.3%	100.0%
		% within q.12.post	100.0%	100.0%	100.0%

Chi-Square Tests

	Value	Exact Sig. (2-sided)
McNemar Test		.000[a]
N of Valid Cases	70	

a. Binomial distribution used.

```
 USE ALL.
COMPUTE filter_$=(group = 1).
VARIABLE LABELS filter_$ 'group = 1 (FILTER)'.
VALUE LABELS filter_$ 0 'Not Selected' 1 'Selected'.
FORMATS filter_$ (f1.0).
FILTER BY filter_$.
EXECUTE.
DESCRIPTIVES VARIABLES=actual.age
  /STATISTICS=MEAN STDDEV MIN MAX.
```

Descriptives

Notes

Output Created		19-SEP-2026 09:21:47
Comments		
Input	Data	F:\\ \asd\ \major.revision. 9.2026 \ASD_Deidentified_Dataset.sav
	Active Dataset	DataSet1
	Filter	group = 1 (FILTER)
	Weight	<none>
	Split File	<none>
	N of Rows in Working Data File	70
Missing Value Handling	Definition of Missing	User defined missing values are treated as missing.
	Cases Used	All non-missing data are used.
Syntax		DESCRIPTIVES VARIABLES=actual.age /STATISTICS=MEAN STDDEV MIN MAX.
Resources	Processor Time	00:00:00.00
	Elapsed Time	00:00:00.00

Descriptive Statistics

	N	Minimum	Maximum	Mean	Std. Deviation
actual.age	70	19.00	45.00	24.2000	4.65506
Valid N (listwise)	70				

```
FREQUENCIES VARIABLES=q.1.pre q.1.post q.2.pre q.2.post q.3.pre q.3.post q.4.p
re q.4.post q.5.pre
    q.5.post q.6.pre q.6.post q.7.pre q.7.post q.8.pre q.8.post q.9.pre q.9.po
st q.10.pre q.10.post
    q.11.pre q.11.post q.12.pre q.12.post q.13.pre q.13.post q.14.pre q.14.pos
t
  /ORDER=ANALYSIS.
```

Frequencies

Notes

Output Created		19-SEP-2026 09:21:59
Comments		
Input	Data	F:\\ \asd\ \major.revision. 9.2026 \ASD_Deidentified_Dataset.sav
	Active Dataset	DataSet1
	Filter	group = 1 (FILTER)
	Weight	<none>
	Split File	<none>
	N of Rows in Working Data File	70
Missing Value Handling	Definition of Missing	User-defined missing values are treated as missing.
	Cases Used	Statistics are based on all cases with valid data.
Syntax		FREQUENCIES VARIABLES=q.1.pre q.1.post q.2.pre q.2.post q.3.pre q.3.post q.4.pre q.4.post q.5.pre q.5.post q.6.pre q.6.post q.7.pre q.7.post q.8.pre q.8.post q.9.pre q.9.post q.10.pre q.10.post q.11.pre q.11.post q.12.pre q.12.post q.13.pre q.13.post q.14.pre q.14.post /ORDER=ANALYSIS.

Notes

Resources	Processor Time	00:00:00.00
	Elapsed Time	00:00:00.00

Statistics

		Children with ASD may show deficits in social reciprocity, such as difficulty with back-and-forth conversation.	q.1.post	Walking problems, such as difficulty climbing stairs, are a core feature of ASD	q.2.post	Deficits in nonverbal communication, such as limited eye contact, are common in ASD	q.3.post
N	Valid	70	70	70	70	70	70
	Missing	0	0	0	0	0	0

Statistics

		Difficulties in developing and maintaining relationships are characteristic of ASD	q.4.post	Repetitive behaviors, such as lining up toys, may be observed in children with ASD	q.5.post	Rigid adherence to routines, such as insisting on taking the same route to school, is common in ASD	q.6.post
N	Valid	70	70	70	70	70	70
	Missing	0	0	0	0	0	0

Statistics

		Children with ASD may display highly restricted or fixated interests, such as focusing on specific objects only.	q.7.post	Unusual responses to sensory input, such as covering ears in response to certain sounds, are associated with ASD	q.8.post	Delayed toilet training is a core diagnostic red flag of ASD	q.9.post
N	Valid	70	70	70	70	70	70
	Missing	0	0	0	0	0	0

Statistics

		Psychosocial interventions play an important role in the management of ASD	q.10.post	Pharmacological treatment may be used to manage associated symptoms of ASD	q.11.post	Dietary interventions alone are an evidence-based treatment for ASD	q.12.post
N	Valid	70	70	70	70	70	70
	Missing	0	0	0	0	0	0

Statistics

		Hyperbaric oxygen therapy is an evidence-based treatment for ASD	q.13.post	Behavioral, developmental, and multidisciplinary educational interventions are effective approaches for managing ASD	q.14.post
N	Valid	70	70	70	70
	Missing	0	0	0	0

Frequency Table

Children with ASD may show deficits in social reciprocity, such as difficulty with back-and-forth conversation.

		Frequency	Percent	Valid Percent	Cumulative Percent
Valid	incorrect	59	84.3	84.3	84.3
	correct	11	15.7	15.7	100.0
	Total	70	100.0	100.0	

q.1.post

		Frequency	Percent	Valid Percent	Cumulative Percent
Valid	incorrect	24	34.3	34.3	34.3
	correct	46	65.7	65.7	100.0
	Total	70	100.0	100.0	

Walking problems, such as difficulty climbing stairs, are a core feature of ASD

		Frequency	Percent	Valid Percent	Cumulative Percent
Valid	incorrect	63	90.0	90.0	90.0
	correct	7	10.0	10.0	100.0
	Total	70	100.0	100.0	

q.2.post

		Frequency	Percent	Valid Percent	Cumulative Percent
Valid	incorrect	22	31.4	31.4	31.4
	correct	48	68.6	68.6	100.0
	Total	70	100.0	100.0	

Deficits in nonverbal communication, such as limited eye contact, are common in ASD

		Frequency	Percent	Valid Percent	Cumulative Percent
Valid	incorrect	62	88.6	88.6	88.6
	correct	8	11.4	11.4	100.0
	Total	70	100.0	100.0	

q.3.post

		Frequency	Percent	Valid Percent	Cumulative Percent
Valid	incorrect	22	31.4	31.4	31.4
	correct	48	68.6	68.6	100.0
	Total	70	100.0	100.0	

Difficulties in developing and maintaining relationships are characteristic of ASD

		Frequency	Percent	Valid Percent	Cumulative Percent
Valid	incorrect	60	85.7	85.7	85.7
	correct	10	14.3	14.3	100.0
	Total	70	100.0	100.0	

q.4.post

		Frequency	Percent	Valid Percent	Cumulative Percent
Valid	incorrect	28	40.0	40.0	40.0
	correct	42	60.0	60.0	100.0
	Total	70	100.0	100.0	

Repetitive behaviors, such as lining up toys, may be observed in children with ASD

		Frequency	Percent	Valid Percent	Cumulative Percent
Valid	incorrect	62	88.6	88.6	88.6
	correct	8	11.4	11.4	100.0
	Total	70	100.0	100.0	

q.5.post

		Frequency	Percent	Valid Percent	Cumulative Percent
Valid	incorrect	31	44.3	44.3	44.3
	correct	39	55.7	55.7	100.0
	Total	70	100.0	100.0	

Rigid adherence to routines, such as insisting on taking the same route to school, is common in ASD

		Frequency	Percent	Valid Percent	Cumulative Percent
Valid	incorrect	61	87.1	87.1	87.1
	correct	9	12.9	12.9	100.0
	Total	70	100.0	100.0	

q.6.post

		Frequency	Percent	Valid Percent	Cumulative Percent
Valid	incorrect	25	35.7	35.7	35.7
	correct	45	64.3	64.3	100.0
	Total	70	100.0	100.0	

Children with ASD may display highly restricted or fixated interests, such as focusing on specific objects only.

		Frequency	Percent	Valid Percent	Cumulative Percent
Valid	incorrect	58	82.9	82.9	82.9
	correct	12	17.1	17.1	100.0
	Total	70	100.0	100.0	

q.7.post

		Frequency	Percent	Valid Percent	Cumulative Percent
Valid	incorrect	24	34.3	34.3	34.3
	correct	46	65.7	65.7	100.0
	Total	70	100.0	100.0	

Unusual responses to sensory input, such as covering ears in response to certain sounds, are associated with ASD

		Frequency	Percent	Valid Percent	Cumulative Percent
Valid	incorrect	59	84.3	84.3	84.3
	correct	11	15.7	15.7	100.0
	Total	70	100.0	100.0	

q.8.post

		Frequency	Percent	Valid Percent	Cumulative Percent
Valid	incorrect	27	38.6	38.6	38.6
	correct	43	61.4	61.4	100.0
	Total	70	100.0	100.0	

Delayed toilet training is a core diagnostic red flag of ASD

		Frequency	Percent	Valid Percent	Cumulative Percent
Valid	incorrect	65	92.9	92.9	92.9
	correct	5	7.1	7.1	100.0
	Total	70	100.0	100.0	

q.9.post

		Frequency	Percent	Valid Percent	Cumulative Percent
Valid	incorrect	30	42.9	42.9	42.9
	correct	40	57.1	57.1	100.0
	Total	70	100.0	100.0	

Psychosocial interventions play an important role in the management of ASD

		Frequency	Percent	Valid Percent	Cumulative Percent
Valid	incorrect	57	81.4	81.4	81.4
	correct	13	18.6	18.6	100.0
	Total	70	100.0	100.0	

q.10.post

		Frequency	Percent	Valid Percent	Cumulative Percent
Valid	incorrect	20	28.6	28.6	28.6
	correct	50	71.4	71.4	100.0
	Total	70	100.0	100.0	

Pharmacological treatment may be used to manage associated symptoms of ASD

		Frequency	Percent	Valid Percent	Cumulative Percent
Valid	incorrect	60	85.7	85.7	85.7
	correct	10	14.3	14.3	100.0
	Total	70	100.0	100.0	

q.11.post

		Frequency	Percent	Valid Percent	Cumulative Percent
Valid	incorrect	18	25.7	25.7	25.7
	correct	52	74.3	74.3	100.0
	Total	70	100.0	100.0	

Dietary interventions alone are an evidence-based treatment for ASD

		Frequency	Percent	Valid Percent	Cumulative Percent
Valid	incorrect	63	90.0	90.0	90.0
	correct	7	10.0	10.0	100.0
	Total	70	100.0	100.0	

q.12.post

		Frequency	Percent	Valid Percent	Cumulative Percent
Valid	incorrect	21	30.0	30.0	30.0
	correct	49	70.0	70.0	100.0
	Total	70	100.0	100.0	

Hyperbaric oxygen therapy is an evidence-based treatment for ASD

		Frequency	Percent	Valid Percent	Cumulative Percent
Valid	incorrect	64	91.4	91.4	91.4
	correct	6	8.6	8.6	100.0
	Total	70	100.0	100.0	

q.13.post

		Frequency	Percent	Valid Percent	Cumulative Percent
Valid	incorrect	22	31.4	31.4	31.4
	correct	48	68.6	68.6	100.0
	Total	70	100.0	100.0	

Behavioral, developmental, and multidisciplinary educational interventions are effective approaches for managing ASD

		Frequency	Percent	Valid Percent	Cumulative Percent
Valid	incorrect	65	92.9	92.9	92.9
	correct	5	7.1	7.1	100.0
	Total	70	100.0	100.0	

q.14.post

		Frequency	Percent	Valid Percent	Cumulative Percent
Valid	incorrect	30	42.9	42.9	42.9
	correct	40	57.1	57.1	100.0
	Total	70	100.0	100.0	

```
CROSSTABS
 /TABLES=q.1.pre BY q.1.post
 /CELLS=COUNT ROW COLUMN
 /STATISTICS=MCNEMAR.
```

Crosstabs

Notes

Output Created		19-SEP-2026 09:22:27
Comments		
Input	Data	F:\\ \asd\ \major.revision.9.2026\ASD_Deidentified_Dataset.sav
	Active Dataset	DataSet1
	Filter	group = 1 (FILTER)
	Weight	<none>
	Split File	<none>
	N of Rows in Working Data File	70
Missing Value Handling	Definition of Missing	User-defined missing values are treated as missing.
	Cases Used	Statistics for each table are based on all the cases with valid data in the specified range(s) for all variables in each table.
Syntax		CROSSTABS /TABLES=q.1.pre BY q.1.post /CELLS=COUNT ROW COLUMN /STATISTICS=MCNEMAR...
Resources	Processor Time	00:00:00.02
	Elapsed Time	00:00:00.02
	Dimensions Requested	2
	Cells Available	524245

Case Processing Summary

	Cases					
	Valid		Missing		Total	
	N	Percent	N	Percent	N	Percent
Children with ASD may show deficits in social reciprocity, such as difficulty with back-and-forth conversation. * q.1.post	70	100.0%	0	0.0%	70	100.0%

Children with ASD may show deficits in social reciprocity, such as difficulty with back-and-forth conversation. * q.1.post Crosstabulation

			q.1.post		
			incorrect	correct	Total
Children with ASD may show deficits in social reciprocity, such as difficulty with back-and-forth conversation.	incorrect	Count	23	36	59
		% within Children with ASD may show deficits in social reciprocity, such as difficulty with back-and-forth conversation.	39.0%	61.0%	100.0%
		% within q.1.post	95.8%	78.3%	84.3%
	correct	Count	1	10	11
		% within Children with ASD may show deficits in social reciprocity, such as difficulty with back-and-forth conversation.	9.1%	90.9%	100.0%
		% within q.1.post	4.2%	21.7%	15.7%
Total		Count	24	46	70
		% within Children with ASD may show deficits in social reciprocity, such as difficulty with back-and-forth conversation.	34.3%	65.7%	100.0%
		% within q.1.post	100.0%	100.0%	100.0%

Chi-Square Tests

	Value	Exact Sig. (2-sided)
McNemar Test		.000[a]
N of Valid Cases	70	

a. Binomial distribution used.

```
CROSSTABS
 /TABLES=q.2.pre BY q.2.post
 /CELLS=COUNT ROW COLUMN
 /STATISTICS=MCNEMAR.
```

Crosstabs

Notes

Output Created		19-SEP-2026 09:22:27
Comments		
Input	Data	F:\\ \asd\ \major.revision. 9.2026 \ASD_Deidentified_Dataset.sav
	Active Dataset	DataSet1
	Filter	group = 1 (FILTER)
	Weight	<none>
	Split File	<none>
	N of Rows in Working Data File	70
Missing Value Handling	Definition of Missing	User-defined missing values are treated as missing.
	Cases Used	Statistics for each table are based on all the cases with valid data in the specified range(s) for all variables in each table.
Syntax		CROSSTABS /TABLES=q.2.pre BY q.2.post /CELLS=COUNT ROW COLUMN /STATISTICS=MCNEMAR...
Resources	Processor Time	00:00:00.02
	Elapsed Time	00:00:00.02
	Dimensions Requested	2
	Cells Available	524245

Case Processing Summary

	Cases					
	Valid		Missing		Total	
	N	Percent	N	Percent	N	Percent
Walking problems, such as difficulty climbing stairs, are a core feature of ASD * q.2.post	70	100.0%	0	0.0%	70	100.0%

Walking problems, such as difficulty climbing stairs, are a core feature of ASD * q.2.post Crosstabulation

			q.2.post		
			incorrect	correct	Total
Walking problems, such as difficulty climbing stairs, are a core feature of ASD	incorrect	Count	22	41	63
		% within Walking problems, such as difficulty climbing stairs, are a core feature of ASD	34.9%	65.1%	100.0%
		% within q.2.post	100.0%	85.4%	90.0%
	correct	Count	0	7	7
		% within Walking problems, such as difficulty climbing stairs, are a core feature of ASD	0.0%	100.0%	100.0%
		% within q.2.post	0.0%	14.6%	10.0%
Total		Count	22	48	70
		% within Walking problems, such as difficulty climbing stairs, are a core feature of ASD	31.4%	68.6%	100.0%
		% within q.2.post	100.0%	100.0%	100.0%

Chi-Square Tests

	Value	Exact Sig. (2-sided)
McNemar Test		.000[a]
N of Valid Cases	70	

a. Binomial distribution used.

```
CROSSTABS
 /TABLES=q.3.pre BY q.3.post
 /CELLS=COUNT ROW COLUMN
 /STATISTICS=MCNEMAR.
```

Crosstabs

Notes

Output Created		19-SEP-2026 09:22:27
Comments		
Input	Data	F:\\ \asd\ \major.revision. 9.2026 \ASD_Deidentified_Dataset.sav
	Active Dataset	DataSet1
	Filter	group = 1 (FILTER)
	Weight	<none>
	Split File	<none>
	N of Rows in Working Data File	70
Missing Value Handling	Definition of Missing	User-defined missing values are treated as missing.
	Cases Used	Statistics for each table are based on all the cases with valid data in the specified range(s) for all variables in each table.
Syntax		CROSSTABS /TABLES=q.3.pre BY q.3.post /CELLS=COUNT ROW COLUMN /STATISTICS=MCNEMAR...
Resources	Processor Time	00:00:00.02
	Elapsed Time	00:00:00.01
	Dimensions Requested	2
	Cells Available	524245

Case Processing Summary

	Cases					
	Valid		Missing		Total	
	N	Percent	N	Percent	N	Percent
Deficits in nonverbal communication, such as limited eye contact, are common in ASD * q.3.post	70	100.0%	0	0.0%	70	100.0%

Deficits in nonverbal communication, such as limited eye contact, are common in ASD * q.3.post Crosstabulation

			q.3.post		
			incorrect	correct	Total
Deficits in nonverbal communication, such as limited eye contact, are common in ASD	incorrect	Count	20	42	62
		% within Deficits in nonverbal communication, such as limited eye contact, are common in ASD	32.3%	67.7%	100.0%
		% within q.3.post	90.9%	87.5%	88.6%
	correct	Count	2	6	8
		% within Deficits in nonverbal communication, such as limited eye contact, are common in ASD	25.0%	75.0%	100.0%
		% within q.3.post	9.1%	12.5%	11.4%
Total		Count	22	48	70
		% within Deficits in nonverbal communication, such as limited eye contact, are common in ASD	31.4%	68.6%	100.0%
		% within q.3.post	100.0%	100.0%	100.0%

Chi-Square Tests

	Value	Exact Sig. (2-sided)
McNemar Test		.000[a]
N of Valid Cases	70	

a. Binomial distribution used.

```
CROSSTABS
 /TABLES=q.4.pre BY q.4.post
 /CELLS=COUNT ROW COLUMN
 /STATISTICS=MCNEMAR.
```

Crosstabs

Notes

Output Created		19-SEP-2026 09:22:27
Comments		
Input	Data	F:\\ \asd\ \major.revision.9.2026\ASD_Deidentified_Dataset.sav
	Active Dataset	DataSet1
	Filter	group = 1 (FILTER)
	Weight	<none>
	Split File	<none>
	N of Rows in Working Data File	70
Missing Value Handling	Definition of Missing	User-defined missing values are treated as missing.
	Cases Used	Statistics for each table are based on all the cases with valid data in the specified range(s) for all variables in each table.
Syntax		CROSSTABS /TABLES=q.4.pre BY q.4.post /CELLS=COUNT ROW COLUMN /STATISTICS=MCNEMAR...
Resources	Processor Time	00:00:00.02
	Elapsed Time	00:00:00.02
	Dimensions Requested	2
	Cells Available	524245

Case Processing Summary

	Cases					
	Valid		Missing		Total	
	N	Percent	N	Percent	N	Percent
Difficulties in developing and maintaining relationships are characteristic of ASD * q.4.post	70	100.0%	0	0.0%	70	100.0%

Difficulties in developing and maintaining relationships are characteristic of ASD * q.4.post Crosstabulation

			q.4.post		
			incorrect	correct	Total
Difficulties in developing and maintaining relationships are characteristic of ASD	incorrect	Count	28	32	60
		% within Difficulties in developing and maintaining relationships are characteristic of ASD	46.7%	53.3%	100.0%
		% within q.4.post	100.0%	76.2%	85.7%
	correct	Count	0	10	10
		% within Difficulties in developing and maintaining relationships are characteristic of ASD	0.0%	100.0%	100.0%
		% within q.4.post	0.0%	23.8%	14.3%
Total		Count	28	42	70
		% within Difficulties in developing and maintaining relationships are characteristic of ASD	40.0%	60.0%	100.0%
		% within q.4.post	100.0%	100.0%	100.0%

Chi-Square Tests

	Value	Exact Sig. (2-sided)
McNemar Test		.000[a]
N of Valid Cases	70	

a. Binomial distribution used.

```
CROSSTABS
 /TABLES=q.5.pre BY q.5.post
 /CELLS=COUNT ROW COLUMN
 /STATISTICS=MCNEMAR.
```

Crosstabs

Notes

Output Created		19-SEP-2026 09:22:27
Comments		
Input	Data	F:\\ \asd\ \major.revision.9.2026\ASD_Deidentified_Dataset.sav
	Active Dataset	DataSet1
	Filter	group = 1 (FILTER)
	Weight	<none>
	Split File	<none>
	N of Rows in Working Data File	70
Missing Value Handling	Definition of Missing	User-defined missing values are treated as missing.
	Cases Used	Statistics for each table are based on all the cases with valid data in the specified range(s) for all variables in each table.
Syntax		CROSSTABS /TABLES=q.5.pre BY q.5.post /CELLS=COUNT ROW COLUMN /STATISTICS=MCNEMAR...
Resources	Processor Time	00:00:00.00
	Elapsed Time	00:00:00.00
	Dimensions Requested	2
	Cells Available	524245

Case Processing Summary

	Cases					
	Valid		Missing		Total	
	N	Percent	N	Percent	N	Percent
Repetitive behaviors, such as lining up toys, may be observed in children with ASD * q.5.post	70	100.0%	0	0.0%	70	100.0%

Repetitive behaviors, such as lining up toys, may be observed in children with ASD * q.5.post Crosstabulation

			q.5.post		
			incorrect	correct	Total
Repetitive behaviors, such as lining up toys, may be observed in children with ASD	incorrect	Count	31	31	62
		% within Repetitive behaviors, such as lining up toys, may be observed in children with ASD	50.0%	50.0%	100.0%
		% within q.5.post	100.0%	79.5%	88.6%
	correct	Count	0	8	8
		% within Repetitive behaviors, such as lining up toys, may be observed in children with ASD	0.0%	100.0%	100.0%
		% within q.5.post	0.0%	20.5%	11.4%
Total		Count	31	39	70
		% within Repetitive behaviors, such as lining up toys, may be observed in children with ASD	44.3%	55.7%	100.0%
		% within q.5.post	100.0%	100.0%	100.0%

Chi-Square Tests

	Value	Exact Sig. (2-sided)
McNemar Test		.000[a]
N of Valid Cases	70	

a. Binomial distribution used.

```
CROSSTABS
 /TABLES=q.6.pre BY q.6.post
 /CELLS=COUNT ROW COLUMN
 /STATISTICS=MCNEMAR.
```

Crosstabs

Notes

Output Created		19-SEP-2026 09:22:27
Comments		
Input	Data	F:\\ \asd\ \major.revision.9.2026\ASD_Deidentified_Dataset.sav
	Active Dataset	DataSet1
	Filter	group = 1 (FILTER)
	Weight	<none>
	Split File	<none>
	N of Rows in Working Data File	70
Missing Value Handling	Definition of Missing	User-defined missing values are treated as missing.
	Cases Used	Statistics for each table are based on all the cases with valid data in the specified range(s) for all variables in each table.
Syntax		CROSSTABS /TABLES=q.6.pre BY q.6.post /CELLS=COUNT ROW COLUMN /STATISTICS=MCNEMAR...
Resources	Processor Time	00:00:00.02
	Elapsed Time	00:00:00.02
	Dimensions Requested	2
	Cells Available	524245

Case Processing Summary

	Cases					
	Valid		Missing		Total	
	N	Percent	N	Percent	N	Percent
Rigid adherence to routines, such as insisting on taking the same route to school, is common in ASD * q.6.post	70	100.0%	0	0.0%	70	100.0%

Rigid adherence to routines, such as insisting on taking the same route to school, is common in ASD * q.6.post Crosstabulation

			q.6.post		
			incorrect	correct	Total
Rigid adherence to routines, such as insisting on taking the same route to school, is common in ASD	incorrect	Count	25	36	61
		% within Rigid adherence to routines, such as insisting on taking the same route to school, is common in ASD	41.0%	59.0%	100.0%
		% within q.6.post	100.0%	80.0%	87.1%
	correct	Count	0	9	9
		% within Rigid adherence to routines, such as insisting on taking the same route to school, is common in ASD	0.0%	100.0%	100.0%
		% within q.6.post	0.0%	20.0%	12.9%
Total		Count	25	45	70
		% within Rigid adherence to routines, such as insisting on taking the same route to school, is common in ASD	35.7%	64.3%	100.0%
		% within q.6.post	100.0%	100.0%	100.0%

Chi-Square Tests

	Value	Exact Sig. (2-sided)
McNemar Test		.000[a]
N of Valid Cases	70	

a. Binomial distribution used.

```
CROSSTABS
 /TABLES=q.7.pre BY q.7.post
 /CELLS=COUNT ROW COLUMN
 /STATISTICS=MCNEMAR.
```

Crosstabs

Notes

Output Created		19-SEP-2026 09:22:27
Comments		
Input	Data	F:\\ \asd\ \major.revision.9.2026\ASD_Deidentified_Dataset.sav
	Active Dataset	DataSet1
	Filter	group = 1 (FILTER)
	Weight	<none>
	Split File	<none>
	N of Rows in Working Data File	70
Missing Value Handling	Definition of Missing	User-defined missing values are treated as missing.
	Cases Used	Statistics for each table are based on all the cases with valid data in the specified range(s) for all variables in each table.
Syntax		CROSSTABS<br>/TABLES=q.7.pre BY q.7.post<br>/CELLS=COUNT ROW COLUMN<br><br>/STATISTICS=MCNEMAR...
Resources	Processor Time	00:00:00.02
	Elapsed Time	00:00:00.02
	Dimensions Requested	2
	Cells Available	524245

Case Processing Summary

	Cases					
	Valid		Missing		Total	
	N	Percent	N	Percent	N	Percent
Children with ASD may display highly restricted or fixated interests, such as focusing on specific objects only. * q.7.post	70	100.0%	0	0.0%	70	100.0%

Children with ASD may display highly restricted or fixated interests, such as focusing on specific objects only. * q.7.post Crosstabulation

			q.7.post		
			incorrect	correct	Total
Children with ASD may display highly restricted or fixated interests, such as focusing on specific objects only.	incorrect	Count	24	34	58
		% within Children with ASD may display highly restricted or fixated interests, such as focusing on specific objects only.	41.4%	58.6%	100.0%
		% within q.7.post	100.0%	73.9%	82.9%
	correct	Count	0	12	12
		% within Children with ASD may display highly restricted or fixated interests, such as focusing on specific objects only.	0.0%	100.0%	100.0%
		% within q.7.post	0.0%	26.1%	17.1%
Total		Count	24	46	70
		% within Children with ASD may display highly restricted or fixated interests, such as focusing on specific objects only.	34.3%	65.7%	100.0%
		% within q.7.post	100.0%	100.0%	100.0%

Chi-Square Tests

	Value	Exact Sig. (2-sided)
McNemar Test		.000[a]
N of Valid Cases	70	

a. Binomial distribution used.

```
CROSSTABS
 /TABLES=q.8.pre BY q.8.post
 /CELLS=COUNT ROW COLUMN
 /STATISTICS=MCNEMAR.
```

Crosstabs

Notes

Output Created		19-SEP-2026 09:22:27
Comments		
Input	Data	F:\\ \asd\ \major.revision. 9.2026 \ASD_Deidentified_Dataset.sav
	Active Dataset	DataSet1
	Filter	group = 1 (FILTER)
	Weight	<none>
	Split File	<none>
	N of Rows in Working Data File	70
Missing Value Handling	Definition of Missing	User-defined missing values are treated as missing.
	Cases Used	Statistics for each table are based on all the cases with valid data in the specified range(s) for all variables in each table.
Syntax		CROSSTABS /TABLES=q.8.pre BY q.8.post /CELLS=COUNT ROW COLUMN /STATISTICS=MCNEMAR...
Resources	Processor Time	00:00:00.00
	Elapsed Time	00:00:00.00
	Dimensions Requested	2
	Cells Available	524245

Case Processing Summary

	Cases					
	Valid		Missing		Total	
	N	Percent	N	Percent	N	Percent
Unusual responses to sensory input, such as covering ears in response to certain sounds, are associated with ASD * q.8.post	70	100.0%	0	0.0%	70	100.0%

Unusual responses to sensory input, such as covering ears in response to certain sounds, are associated with ASD * q.8.post Crosstabulation

			q.8.post		
			incorrect	correct	Total
Unusual responses to sensory input, such as covering ears in response to certain sounds, are associated with ASD	incorrect	Count	26	33	59
		% within Unusual responses to sensory input, such as covering ears in response to certain sounds, are associated with ASD	44.1%	55.9%	100.0%
		% within q.8.post	96.3%	76.7%	84.3%
	correct	Count	1	10	11
		% within Unusual responses to sensory input, such as covering ears in response to certain sounds, are associated with ASD	9.1%	90.9%	100.0%
		% within q.8.post	3.7%	23.3%	15.7%
Total		Count	27	43	70
		% within Unusual responses to sensory input, such as covering ears in response to certain sounds, are associated with ASD	38.6%	61.4%	100.0%
		% within q.8.post	100.0%	100.0%	100.0%

Chi-Square Tests

	Value	Exact Sig. (2-sided)
McNemar Test		.000[a]
N of Valid Cases	70	

a. Binomial distribution used.

```
CROSSTABS
 /TABLES=q.9.pre BY q.9.post
 /CELLS=COUNT ROW COLUMN
 /STATISTICS=MCNEMAR.
```

Crosstabs

Notes

Output Created		19-SEP-2026 09:22:27
Comments		
Input	Data	F:\\ \asd\ \major.revision.9.2026\ASD_Deidentified_Dataset.sav
	Active Dataset	DataSet1
	Filter	group = 1 (FILTER)
	Weight	<none>
	Split File	<none>
	N of Rows in Working Data File	70
Missing Value Handling	Definition of Missing	User-defined missing values are treated as missing.
	Cases Used	Statistics for each table are based on all the cases with valid data in the specified range(s) for all variables in each table.

Notes

Syntax		CROSSTABS<br>/TABLES=q.9.pre BY q.9.post<br>/CELLS=COUNT ROW COLUMN<br><br>/STATISTICS=MCNEMAR...
Resources	Processor Time	00:00:00.00
	Elapsed Time	00:00:00.00
	Dimensions Requested	2
	Cells Available	524245

Case Processing Summary

	Cases					
	Valid		Missing		Total	
	N	Percent	N	Percent	N	Percent
Delayed toilet training is a core diagnostic red flag of ASD * q.9.post	70	100.0%	0	0.0%	70	100.0%

Delayed toilet training is a core diagnostic red flag of ASD * q.9.post Crosstabulation

			q.9.post		
			incorrect	correct	Total
Delayed toilet training is a core diagnostic red flag of ASD	incorrect	Count	30	35	65
		% within Delayed toilet training is a core diagnostic red flag of ASD	46.2%	53.8%	100.0%
		% within q.9.post	100.0%	87.5%	92.9%
	correct	Count	0	5	5
		% within Delayed toilet training is a core diagnostic red flag of ASD	0.0%	100.0%	100.0%
		% within q.9.post	0.0%	12.5%	7.1%
Total		Count	30	40	70
		% within Delayed toilet training is a core diagnostic red flag of ASD	42.9%	57.1%	100.0%
		% within q.9.post	100.0%	100.0%	100.0%

Chi-Square Tests

	Value	Exact Sig. (2-sided)
McNemar Test		.000[a]
N of Valid Cases	70	

a. Binomial distribution used.

```
CROSSTABS
  /TABLES=q.10.pre BY q.10.post
  /CELLS=COUNT ROW COLUMN
  /STATISTICS=MCNEMAR.
```

Crosstabs

Notes

Output Created		19-SEP-2026 09:22:27
Comments		
Input	Data	F:\\ \asd\ \major.revision. 9.2026 \ASD_Deidentified_Dataset.sav
	Active Dataset	DataSet1
	Filter	group = 1 (FILTER)
	Weight	<none>
	Split File	<none>
	N of Rows in Working Data File	70
Missing Value Handling	Definition of Missing	User-defined missing values are treated as missing.
	Cases Used	Statistics for each table are based on all the cases with valid data in the specified range(s) for all variables in each table.

Notes

Syntax		CROSSTABS<br>/TABLES=q.10.pre BY q.10.post<br>/CELLS=COUNT ROW COLUMN<br><br>/STATISTICS=MCNEMAR...
Resources	Processor Time	00:00:00.03
	Elapsed Time	00:00:00.02
	Dimensions Requested	2
	Cells Available	524245

Case Processing Summary

	Cases					
	Valid		Missing		Total	
	N	Percent	N	Percent	N	Percent
Psychosocial interventions play an important role in the management of ASD * q.10.post	70	100.0%	0	0.0%	70	100.0%

Psychosocial interventions play an important role in the management of ASD * q.10.post Crosstabulation

			q.10.post		Total
			incorrect	correct	
Psychosocial interventions play an important role in the management of ASD	incorrect	Count	20	37	57
		% within Psychosocial interventions play an important role in the management of ASD	35.1%	64.9%	100.0%
		% within q.10.post	100.0%	74.0%	81.4%
	correct	Count	0	13	13
		% within Psychosocial interventions play an important role in the management of ASD	0.0%	100.0%	100.0%
		% within q.10.post	0.0%	26.0%	18.6%
Total		Count	20	50	70
		% within Psychosocial interventions play an important role in the management of ASD	28.6%	71.4%	100.0%
		% within q.10.post	100.0%	100.0%	100.0%

Chi-Square Tests

	Value	Exact Sig. (2-sided)
McNemar Test		.000[a]
N of Valid Cases	70	

a. Binomial distribution used.

```
CROSSTABS
 /TABLES=q.11.pre BY q.11.post
 /CELLS=COUNT ROW COLUMN
 /STATISTICS=MCNEMAR.
```

Crosstabs

Notes

Output Created		19-SEP-2026 09:22:27
Comments		
Input	Data	F:\\ \asd\ \major.revision.9.2026\ASD_Deidentified_Dataset.sav
	Active Dataset	DataSet1
	Filter	group = 1 (FILTER)
	Weight	<none>
	Split File	<none>
	N of Rows in Working Data File	70
Missing Value Handling	Definition of Missing	User-defined missing values are treated as missing.
	Cases Used	Statistics for each table are based on all the cases with valid data in the specified range(s) for all variables in each table.
Syntax		CROSSTABS /TABLES=q.11.pre BY q.11.post /CELLS=COUNT ROW COLUMN /STATISTICS=MCNEMAR...
Resources	Processor Time	00:00:00.00
	Elapsed Time	00:00:00.00
	Dimensions Requested	2
	Cells Available	524245

Case Processing Summary

	Cases					
	Valid		Missing		Total	
	N	Percent	N	Percent	N	Percent
Pharmacological treatment may be used to manage associated symptoms of ASD * q.11.post	70	100.0%	0	0.0%	70	100.0%

Pharmacological treatment may be used to manage associated symptoms of ASD * q.11.post Crosstabulation

			q.11.post		
			incorrect	correct	Total
Pharmacological treatment may be used to manage associated symptoms of ASD	incorrect	Count	18	42	60
		% within Pharmacological treatment may be used to manage associated symptoms of ASD	30.0%	70.0%	100.0%
		% within q.11.post	100.0%	80.8%	85.7%
	correct	Count	0	10	10
		% within Pharmacological treatment may be used to manage associated symptoms of ASD	0.0%	100.0%	100.0%
		% within q.11.post	0.0%	19.2%	14.3%
Total		Count	18	52	70
		% within Pharmacological treatment may be used to manage associated symptoms of ASD	25.7%	74.3%	100.0%
		% within q.11.post	100.0%	100.0%	100.0%

Chi-Square Tests

	Value	Exact Sig. (2-sided)
McNemar Test		.000[a]
N of Valid Cases	70	

a. Binomial distribution used.

```
CROSSTABS
 /TABLES=q.12.pre BY q.12.post
 /CELLS=COUNT ROW COLUMN
 /STATISTICS=MCNEMAR.
```

Crosstabs

Notes

Output Created		19-SEP-2026 09:22:27
Comments		
Input	Data	F:\\ \asd\ \major.revision. 9.2026 \ASD_Deidentified_Dataset.sav
	Active Dataset	DataSet1
	Filter	group = 1 (FILTER)
	Weight	<none>
	Split File	<none>
	N of Rows in Working Data File	70
Missing Value Handling	Definition of Missing	User-defined missing values are treated as missing.
	Cases Used	Statistics for each table are based on all the cases with valid data in the specified range(s) for all variables in each table.
Syntax		CROSSTABS /TABLES=q.12.pre BY q.12.post /CELLS=COUNT ROW COLUMN /STATISTICS=MCNEMAR...
Resources	Processor Time	00:00:00.02
	Elapsed Time	00:00:00.02
	Dimensions Requested	2
	Cells Available	524245

Case Processing Summary

	Cases					
	Valid		Missing		Total	
	N	Percent	N	Percent	N	Percent
Dietary interventions alone are an evidence-based treatment for ASD * q.12.post	70	100.0%	0	0.0%	70	100.0%

Dietary interventions alone are an evidence-based treatment for ASD * q.12.post Crosstabulation

			q.12.post		
			incorrect	correct	Total
Dietary interventions alone are an evidence-based treatment for ASD	incorrect	Count	18	45	63
		% within Dietary interventions alone are an evidence-based treatment for ASD	28.6%	71.4%	100.0%
		% within q.12.post	85.7%	91.8%	90.0%
	correct	Count	3	4	7
		% within Dietary interventions alone are an evidence-based treatment for ASD	42.9%	57.1%	100.0%
		% within q.12.post	14.3%	8.2%	10.0%
Total		Count	21	49	70
		% within Dietary interventions alone are an evidence-based treatment for ASD	30.0%	70.0%	100.0%
		% within q.12.post	100.0%	100.0%	100.0%

Chi-Square Tests

	Value	Exact Sig. (2-sided)
McNemar Test		.000[a]
N of Valid Cases	70	

a. Binomial distribution used.

```
CROSSTABS
 /TABLES=q.13.pre BY q.13.post
 /CELLS=COUNT ROW COLUMN
 /STATISTICS=MCNEMAR.
```

Crosstabs

Notes

Output Created		19-SEP-2026 09:22:27
Comments		
Input	Data	F:\\ \asd\ \major.revision. 9.2026 \ASD_Deidentified_Dataset.sav
	Active Dataset	DataSet1
	Filter	group = 1 (FILTER)
	Weight	<none>
	Split File	<none>
	N of Rows in Working Data File	70
Missing Value Handling	Definition of Missing	User-defined missing values are treated as missing.
	Cases Used	Statistics for each table are based on all the cases with valid data in the specified range(s) for all variables in each table.
Syntax		CROSSTABS /TABLES=q.13.pre BY q.13.post /CELLS=COUNT ROW COLUMN /STATISTICS=MCNEMAR...
Resources	Processor Time	00:00:00.02
	Elapsed Time	00:00:00.01
	Dimensions Requested	2
	Cells Available	524245

Case Processing Summary

	Cases					
	Valid		Missing		Total	
	N	Percent	N	Percent	N	Percent
Hyperbaric oxygen therapy is an evidence-based treatment for ASD * q.13.post	70	100.0%	0	0.0%	70	100.0%

Hyperbaric oxygen therapy is an evidence-based treatment for ASD * q.13.post Crosstabulation

			q.13.post		
			incorrect	correct	Total
Hyperbaric oxygen therapy is an evidence-based treatment for ASD	incorrect	Count	22	42	64
		% within Hyperbaric oxygen therapy is an evidence-based treatment for ASD	34.4%	65.6%	100.0%
		% within q.13.post	100.0%	87.5%	91.4%
	correct	Count	0	6	6
		% within Hyperbaric oxygen therapy is an evidence-based treatment for ASD	0.0%	100.0%	100.0%
		% within q.13.post	0.0%	12.5%	8.6%
Total		Count	22	48	70
		% within Hyperbaric oxygen therapy is an evidence-based treatment for ASD	31.4%	68.6%	100.0%
		% within q.13.post	100.0%	100.0%	100.0%

Chi-Square Tests

	Value	Exact Sig. (2-sided)
McNemar Test		.000[a]
N of Valid Cases	70	

a. Binomial distribution used.

```
CROSSTABS
  /TABLES=q.14.pre BY q.14.post
  /CELLS=COUNT ROW COLUMN
  /STATISTICS=MCNEMAR.
```

Crosstabs

Notes

Output Created		19-SEP-2026 09:22:27
Comments		
Input	Data	F:\\ \asd\ \major.revision.9.2026\ASD_Deidentified_Dataset.sav
	Active Dataset	DataSet1
	Filter	group = 1 (FILTER)
	Weight	<none>
	Split File	<none>
	N of Rows in Working Data File	70
Missing Value Handling	Definition of Missing	User-defined missing values are treated as missing.
	Cases Used	Statistics for each table are based on all the cases with valid data in the specified range(s) for all variables in each table.
Syntax		CROSSTABS /TABLES=q.14.pre BY q.14.post /CELLS=COUNT ROW COLUMN /STATISTICS=MCNEMAR...
Resources	Processor Time	00:00:00.00
	Elapsed Time	00:00:00.00
	Dimensions Requested	2
	Cells Available	524245

Case Processing Summary

	Cases					
	Valid		Missing		Total	
	N	Percent	N	Percent	N	Percent
Behavioral, developmental, and multidisciplinary educational interventions are effective approaches for managing ASD * q.14.post	70	100.0%	0	0.0%	70	100.0%

Behavioral, developmental, and multidisciplinary educational interventions are effective approaches for managing ASD * q.14.post Crosstabulation

			q.14.post		Total
			incorrect	correct	
Behavioral, developmental, and multidisciplinary educational interventions are effective approaches for managing ASD	incorrect	Count	30	35	65
		% within Behavioral, developmental, and multidisciplinary educational interventions are effective approaches for managing ASD	46.2%	53.8%	100.0%
		% within q.14.post	100.0%	87.5%	92.9%
	correct	Count	0	5	5
		% within Behavioral, developmental, and multidisciplinary educational interventions are effective approaches for managing ASD	0.0%	100.0%	100.0%
		% within q.14.post	0.0%	12.5%	7.1%
Total		Count	30	40	70
		% within Behavioral, developmental, and multidisciplinary educational interventions are effective approaches for managing ASD	42.9%	57.1%	100.0%
		% within q.14.post	100.0%	100.0%	100.0%

Chi-Square Tests

	Value	Exact Sig. (2-sided)
McNemar Test		.000[a]
N of Valid Cases	70	

a. Binomial distribution used.

Notes

Output Created		19-SEP-2026 09:25:33
Comments		
Input	Data	F:\\ \asd\ \major.revision.9.2026\ASD_Deidentified_Dataset.sav
	Active Dataset	DataSet1
	Filter	group = 0 (FILTER)
	Weight	<none>
	Split File	<none>
Missing Value Handling	Definition of Missing	User defined missing values are treated as missing.
	Cases Used	Statistics for each analysis are based on the cases with no missing or out-of-range data for any variable in the analysis.
Syntax		T-TEST PAIRS=beliefs.6.pre WITH beliefs.6.post (PAIRED) /CRITERIA=CI(.95) /MISSING=ANALYSIS.
Resources	Processor Time	00:00:00.00
	Elapsed Time	00:00:00.00

Notes

Output Created		19-SEP-2026 09:25:33
Comments		
Input	Data	F:\\ \asd\ \major.revision. 9.2026 \ASD_Deidentified_Dataset.sav
	Active Dataset	DataSet1
	Filter	group = 0 (FILTER)
	Weight	<none>
	Split File	<none>
Missing Value Handling	Definition of Missing	User defined missing values are treated as missing.
	Cases Used	Statistics for each analysis are based on the cases with no missing or out-of-range data for any variable in the analysis.
Syntax		T-TEST PAIRS=beliefs.7.pre WITH beliefs.7.post (PAIRED) /CRITERIA=CI(.95) /MISSING=ANALYSIS.
Resources	Processor Time	00:00:00.00
	Elapsed Time	00:00:00.00

Notes

Output Created		19-SEP-2026 09:25:33
Comments		
Input	Data	F:\\ \asd\ \major.revision.9.2026\ASD_Deidentified_Dataset.sav
	Active Dataset	DataSet1
	Filter	group = 0 (FILTER)
	Weight	<none>
	Split File	<none>
Missing Value Handling	Definition of Missing	User defined missing values are treated as missing.
	Cases Used	Statistics for each analysis are based on the cases with no missing or out-of-range data for any variable in the analysis.
Syntax		T-TEST PAIRS=beliefs.8.pre WITH beliefs.8.post (PAIRED) /CRITERIA=CI(.95) /MISSING=ANALYSIS.
Resources	Processor Time	00:00:00.02
	Elapsed Time	00:00:00.00

Notes

Output Created		19-SEP-2026 09:27:11
Comments		
Input	Data	F:\\ \asd\ \major.revision. 9.2026 \ASD_Deidentified_Dataset.sav
	Active Dataset	DataSet1
	Filter	group = 0 (FILTER)
	Weight	<none>
	Split File	<none>
	N of Rows in Working Data File	70
Missing Value Handling	Definition of Missing	User defined missing values are treated as missing.
	Cases Used	Statistics for each analysis are based on the cases with no missing or out-of-range data for any variable in the analysis.
Syntax		T-TEST PAIRS=Belife.1 WITH belife.1.post (PAIRED) /CRITERIA=CI(.95) /MISSING=ANALYSIS.
Resources	Processor Time	00:00:00.00
	Elapsed Time	00:00:00.14

Paired Samples Statistics

		Mean	N	Std. Deviation	Std. Error Mean
Pair 1	Belife.1	1.3429	70	.53530	.06398
	belife.1.post	2.9000	70	.30217	.03612

Paired Samples Correlations

		N	Correlation	Sig.
Pair 1	Belife.1 & belife.1.post	70	-.143	.236

Paired Samples Test

		Paired Differences			
					95% Confidence ...
		Mean	Std. Deviation	Std. Error Mean	Lower
Pair 1	Belife.1 - belife.1.post	-1.55714	.65132	.07785	-1.71245

Paired Samples Test

		Paired ...			
		95% Confidence Interval of the ...			
		Upper	t	df	Sig. (2-tailed)
Pair 1	Belife.1 - belife.1.post	-1.40184	-20.002	69	.000

```
 T-TEST PAIRS=Belife.2 WITH belife.2.post (PAIRED)
/CRITERIA=CI(.95)
/MISSING=ANALYSIS.
```

T-Test

Notes

Output Created		19-SEP-2026 09:27:11
Comments		
Input	Data	F:\\ \asd\ \major.revision. 9.2026 \ASD_Deidentified_Dataset.sav
	Active Dataset	DataSet1
	Filter	group = 0 (FILTER)
	Weight	<none>
	Split File	<none>
	N of Rows in Working Data File	70
Missing Value Handling	Definition of Missing	User defined missing values are treated as missing.
	Cases Used	Statistics for each analysis are based on the cases with no missing or out-of-range data for any variable in the analysis.
Syntax		T-TEST PAIRS=Belife.2 WITH belife.2.post (PAIRED) /CRITERIA=CI(.95) /MISSING=ANALYSIS.
Resources	Processor Time	00:00:00.02
	Elapsed Time	00:00:00.01

Paired Samples Statistics

		Mean	N	Std. Deviation	Std. Error Mean
Pair 1	Belife.2	1.2857	70	.45502	.05438
	belife.2.post	2.7857	70	.53549	.06400

Paired Samples Correlations

		N	Correlation	Sig.
Pair 1	Belife.2 & belife.2.post	70	.017	.889

Paired Samples Test

		Paired Differences			
					95% Confidence ...
		Mean	Std. Deviation	Std. Error Mean	Lower
Pair 1	Belife.2 - belife.2.post	-1.50000	.69678	.08328	-1.66614

Paired Samples Test

		Paired ...			
		95% Confidence Interval of the ...			
		Upper	t	df	Sig. (2-tailed)
Pair 1	Belife.2 - belife.2.post	-1.33386	-18.011	69	.000

```
 T-TEST PAIRS=Belife.3 WITH belife.3.post (PAIRED)
/CRITERIA=CI(.95)
/MISSING=ANALYSIS.
```

T-Test

Notes

Output Created		19-SEP-2026 09:27:11
Comments		
Input	Data	F:\\ \asd\ \major.revision.9.2026\ASD_Deidentified_Dataset.sav
	Active Dataset	DataSet1
	Filter	group = 0 (FILTER)
	Weight	<none>
	Split File	<none>
	N of Rows in Working Data File	70
Missing Value Handling	Definition of Missing	User defined missing values are treated as missing.
	Cases Used	Statistics for each analysis are based on the cases with no missing or out-of-range data for any variable in the analysis.
Syntax		T-TEST PAIRS=Belife.3 WITH belife.3.post (PAIRED) /CRITERIA=CI(.95) /MISSING=ANALYSIS.
Resources	Processor Time	00:00:00.00
	Elapsed Time	00:00:00.00

Paired Samples Statistics

		Mean	N	Std. Deviation	Std. Error Mean
Pair 1	Belife.3	1.3000	70	.46157	.05517
	belife.3.post	2.7857	70	.50770	.06068

Paired Samples Correlations

		N	Correlation	Sig.
Pair 1	Belife.3 & belife.3.post	70	.031	.799

Paired Samples Test

		Paired Differences			
					95% Confidence ...
		Mean	Std. Deviation	Std. Error Mean	Lower
Pair 1	Belife.3 - belife.3.post	-1.48571	.67551	.08074	-1.64678

Paired Samples Test

		Paired ...			
		95% Confidence Interval of the ...			
		Upper	t	df	Sig. (2-tailed)
Pair 1	Belife.3 - belife.3.post	-1.32464	-18.401	69	.000

```
 T-TEST PAIRS=Belife.4 WITH belife.4.post (PAIRED)
/CRITERIA=CI(.95)
/MISSING=ANALYSIS.
```

T-Test

Notes

Output Created		19-SEP-2026 09:27:11
Comments		
Input	Data	F:\\ \asd\ \major.revision.9.2026\ASD_Deidentified_Dataset.sav
	Active Dataset	DataSet1
	Filter	group = 0 (FILTER)
	Weight	<none>
	Split File	<none>
	N of Rows in Working Data File	70
Missing Value Handling	Definition of Missing	User defined missing values are treated as missing.
	Cases Used	Statistics for each analysis are based on the cases with no missing or out-of-range data for any variable in the analysis.
Syntax		T-TEST PAIRS=Belife.4 WITH belife.4.post (PAIRED)<br>/CRITERIA=CI(.95)<br>/MISSING=ANALYSIS.
Resources	Processor Time	00:00:00.02
	Elapsed Time	00:00:00.01

Paired Samples Statistics

		Mean	N	Std. Deviation	Std. Error Mean
Pair 1	Belife.4	1.4571	70	.62983	.07528
	belife.4.post	2.8429	70	.36656	.04381

Paired Samples Correlations

		N	Correlation	Sig.
Pair 1	Belife.4 & belife.4.post	70	.065	.595

Paired Samples Test

		Paired Differences			
					95% Confidence ...
		Mean	Std. Deviation	Std. Error Mean	Lower
Pair 1	Belife.4 - belife.4.post	-1.38571	.70798	.08462	-1.55453

Paired Samples Test

		Paired ...			
		95% Confidence Interval of the ...			
		Upper	t	df	Sig. (2-tailed)
Pair 1	Belife.4 - belife.4.post	-1.21690	-16.376	69	.000

```
 T-TEST PAIRS=Belife.5 WITH belife.5.post (PAIRED)
/CRITERIA=CI(.95)
/MISSING=ANALYSIS.
```

T-Test

Notes

Output Created		19-SEP-2026 09:27:11
Comments		
Input	Data	F:\\ \asd\ \major.revision.9.2026\ASD_Deidentified_Dataset.sav
	Active Dataset	DataSet1
	Filter	group = 0 (FILTER)
	Weight	<none>
	Split File	<none>
	N of Rows in Working Data File	70
Missing Value Handling	Definition of Missing	User defined missing values are treated as missing.
	Cases Used	Statistics for each analysis are based on the cases with no missing or out-of-range data for any variable in the analysis.
Syntax		T-TEST PAIRS=Belife.5 WITH belife.5.post (PAIRED) /CRITERIA=CI(.95) /MISSING=ANALYSIS.
Resources	Processor Time	00:00:00.02
	Elapsed Time	00:00:00.01

Paired Samples Statistics

		Mean	N	Std. Deviation	Std. Error Mean
Pair 1	Belife.5	1.4143	70	.55149	.06592
	belife.5.post	2.9286	70	.25940	.03100

Paired Samples Correlations

		N	Correlation	Sig.
Pair 1	Belife.5 & belife.5.post	70	-.094	.439

Paired Samples Test

		Paired Differences			
					95% Confidence ...
		Mean	Std. Deviation	Std. Error Mean	Lower
Pair 1	Belife.5 - belife.5.post	-1.51429	.63114	.07544	-1.66478

Paired Samples Test

		Paired ...			
		95% Confidence Interval of the ...			
		Upper	t	df	Sig. (2-tailed)
Pair 1	Belife.5 - belife.5.post	-1.36379	-20.074	69	.000

```
 T-TEST PAIRS=Belife.6 WITH belife.6.post (PAIRED)
/CRITERIA=CI(.95)
/MISSING=ANALYSIS.
```

T-Test

Notes

Output Created		19-SEP-2026 09:27:11
Comments		
Input	Data	F:\\ \asd\ \major.revision. 9.2026 \ASD_Deidentified_Dataset.sav
	Active Dataset	DataSet1
	Filter	group = 0 (FILTER)
	Weight	<none>
	Split File	<none>
	N of Rows in Working Data File	70
Missing Value Handling	Definition of Missing	User defined missing values are treated as missing.
	Cases Used	Statistics for each analysis are based on the cases with no missing or out-of-range data for any variable in the analysis.
Syntax		T-TEST PAIRS=Belife.6 WITH belife.6.post (PAIRED) /CRITERIA=CI(.95) /MISSING=ANALYSIS.
Resources	Processor Time	00:00:00.00
	Elapsed Time	00:00:00.01

Paired Samples Statistics

		Mean	N	Std. Deviation	Std. Error Mean
Pair 1	Belife.6	1.3857	70	.57213	.06838
	belife.6.post	2.9429	70	.23379	.02794

Paired Samples Correlations

		N	Correlation	Sig.
Pair 1	Belife.6 & belife.6.post	70	-.050	.684

Paired Samples Test

		Paired Differences			
					95% Confidence ...
		Mean	Std. Deviation	Std. Error Mean	Lower
Pair 1	Belife.6 - belife.6.post	-1.55714	.62868	.07514	-1.70705

Paired Samples Test

		Paired ...			
		95% Confidence Interval of the ...			
		Upper	t	df	Sig. (2-tailed)
Pair 1	Belife.6 - belife.6.post	-1.40724	-20.723	69	.000

```
 T-TEST PAIRS=Belife.7 WITH belife.7.post (PAIRED)
/CRITERIA=CI(.95)
/MISSING=ANALYSIS.
```

T-Test

Notes

Output Created		19-SEP-2026 09:27:11
Comments		
Input	Data	F:\\ \asd\ \major.revision.9.2026 \ASD_Deidentified_Dataset.sav
	Active Dataset	DataSet1
	Filter	group = 0 (FILTER)
	Weight	<none>
	Split File	<none>
	N of Rows in Working Data File	70
Missing Value Handling	Definition of Missing	User defined missing values are treated as missing.
	Cases Used	Statistics for each analysis are based on the cases with no missing or out-of-range data for any variable in the analysis.
Syntax		T-TEST PAIRS=Belife.7 WITH belife.7.post (PAIRED) /CRITERIA=CI(.95) /MISSING=ANALYSIS.
Resources	Processor Time	00:00:00.02
	Elapsed Time	00:00:00.01

Paired Samples Statistics

		Mean	N	Std. Deviation	Std. Error Mean
Pair 1	Belife.7	1.4429	70	.62868	.07514
	belife.7.post	2.9143	70	.32938	.03937

Paired Samples Correlations

		N	Correlation	Sig.
Pair 1	Belife.7 & belife.7.post	70	.046	.705

Paired Samples Test

		Paired Differences			
					95% Confidence ...
		Mean	Std. Deviation	Std. Error Mean	Lower
Pair 1	Belife.7 - belife.7.post	-1.47143	.69619	.08321	-1.63743

Paired Samples Test

		Paired ...			
		95% Confidence Interval of the ...			
		Upper	t	df	Sig. (2-tailed)
Pair 1	Belife.7 - belife.7.post	-1.30543	-17.683	69	.000

```
 T-TEST PAIRS=Belife.8 WITH belife.8.post (PAIRED)
/CRITERIA=CI(.95)
/MISSING=ANALYSIS.
```

T-Test

Notes

Output Created		19-SEP-2026 09:27:11
Comments		
Input	Data	F:\\ \asd\ \major.revision. 9.2026 \ASD_Deidentified_Dataset.sav
	Active Dataset	DataSet1
	Filter	group = 0 (FILTER)
	Weight	<none>
	Split File	<none>
	N of Rows in Working Data File	70
Missing Value Handling	Definition of Missing	User defined missing values are treated as missing.
	Cases Used	Statistics for each analysis are based on the cases with no missing or out-of-range data for any variable in the analysis.
Syntax		T-TEST PAIRS=Belife.8 WITH belife.8.post (PAIRED) /CRITERIA=CI(.95) /MISSING=ANALYSIS.
Resources	Processor Time	00:00:00.00
	Elapsed Time	00:00:00.01

Paired Samples Statistics

		Mean	N	Std. Deviation	Std. Error Mean
Pair 1	Belife.8	1.2714	70	.44791	.05354
	belife.8.post	2.7857	70	.53549	.06400

Paired Samples Correlations

		N	Correlation	Sig.
Pair 1	Belife.8 & belife.8.post	70	.004	.972

Paired Samples Test

		Paired Differences			
					95% Confidence ...
		Mean	Std. Deviation	Std. Error Mean	Lower
Pair 1	Belife.8 - belife.8.post	-1.51429	.69663	.08326	-1.68039

Paired Samples Test

		Paired ...			
		95% Confidence Interval of the ...			
		Upper	t	df	Sig. (2-tailed)
Pair 1	Belife.8 - belife.8.post	-1.34818	-18.187	69	.000

```
 USE ALL.
COMPUTE filter_$=(group = 1).
VARIABLE LABELS filter_$ 'group = 1 (FILTER)'.
VALUE LABELS filter_$ 0 'Not Selected' 1 'Selected'.
FORMATS filter_$ (f1.0).
FILTER BY filter_$.
EXECUTE.
T-TEST PAIRS=Belife.1 WITH belife.1.post (PAIRED)
/CRITERIA=CI(.95)
/MISSING=ANALYSIS.
```

T-Test

Notes

Output Created		19-SEP-2026 09:38:18
Comments		
Input	Data	F:\\ \asd\ \major.revision. 9.2026 \ASD_Deidentified_Dataset.sav
	Active Dataset	DataSet1
	Filter	group = 1 (FILTER)
	Weight	<none>
	Split File	<none>
	N of Rows in Working Data File	70
Missing Value Handling	Definition of Missing	User defined missing values are treated as missing.
	Cases Used	Statistics for each analysis are based on the cases with no missing or out-of-range data for any variable in the analysis.
Syntax		T-TEST PAIRS=Belife.1 WITH belife.1.post (PAIRED) /CRITERIA=CI(.95) /MISSING=ANALYSIS.
Resources	Processor Time	00:00:00.02
	Elapsed Time	00:00:00.02

Paired Samples Statistics

		Mean	N	Std. Deviation	Std. Error Mean
Pair 1	Belife.1	1.3857	70	.57213	.06838
	belife.1.post	2.5000	70	.58359	.06975

Paired Samples Correlations

		N	Correlation	Sig.
Pair 1	Belife.1 & belife.1.post	70	.109	.371

Paired Samples Test

		Paired Differences			
					95% Confidence ...
		Mean	Std. Deviation	Std. Error Mean	Lower
Pair 1	Belife.1 - belife.1.post	-1.11429	.77165	.09223	-1.29828

Paired Samples Test

		Paired ...			
		95% Confidence Interval of the ...			
		Upper	t	df	Sig. (2-tailed)
Pair 1	Belife.1 - belife.1.post	-.93029	-12.082	69	.000

```
 T-TEST PAIRS=Belife.2 WITH belife.2.post (PAIRED)
/CRITERIA=CI(.95)
/MISSING=ANALYSIS.
```

T-Test

Notes

Output Created		19-SEP-2026 09:38:18
Comments		
Input	Data	F:\\ \asd\ \major.revision.9.2026\ASD_Deidentified_Dataset.sav
	Active Dataset	DataSet1
	Filter	group = 1 (FILTER)
	Weight	<none>
	Split File	<none>
	N of Rows in Working Data File	70
Missing Value Handling	Definition of Missing	User defined missing values are treated as missing.
	Cases Used	Statistics for each analysis are based on the cases with no missing or out-of-range data for any variable in the analysis.
Syntax		T-TEST PAIRS=Belife.2 WITH belife.2.post (PAIRED) /CRITERIA=CI(.95) /MISSING=ANALYSIS.
Resources	Processor Time	00:00:00.02
	Elapsed Time	00:00:00.02

Paired Samples Statistics

		Mean	N	Std. Deviation	Std. Error Mean
Pair 1	Belife.2	1.3429	70	.50750	.06066
	belife.2.post	2.4286	70	.64989	.07768

Paired Samples Correlations

		N	Correlation	Sig.
Pair 1	Belife.2 & belife.2.post	70	-.013	.918

Paired Samples Test

		Paired Differences			
					95% Confidence ...
		Mean	Std. Deviation	Std. Error Mean	Lower
Pair 1	Belife.2 - belife.2.post	-1.08571	.82958	.09915	-1.28352

Paired Samples Test

		Paired ...			
		95% Confidence Interval of the ...			
		Upper	t	df	Sig. (2-tailed)
Pair 1	Belife.2 - belife.2.post	-.88791	-10.950	69	.000

```
 T-TEST PAIRS=Belife.3 WITH belife.3.post (PAIRED)
/CRITERIA=CI(.95)
/MISSING=ANALYSIS.
```

T-Test

Notes

Output Created		19-SEP-2026 09:38:18
Comments		
Input	Data	F:\\ \asd\ \major.revision. 9.2026 \ASD_Deidentified_Dataset.sav
	Active Dataset	DataSet1
	Filter	group = 1 (FILTER)
	Weight	<none>
	Split File	<none>
	N of Rows in Working Data File	70
Missing Value Handling	Definition of Missing	User defined missing values are treated as missing.
	Cases Used	Statistics for each analysis are based on the cases with no missing or out-of-range data for any variable in the analysis.
Syntax		T-TEST PAIRS=Belife.3 WITH belife.3.post (PAIRED) /CRITERIA=CI(.95) /MISSING=ANALYSIS.
Resources	Processor Time	00:00:00.02
	Elapsed Time	00:00:00.02

Paired Samples Statistics

		Mean	N	Std. Deviation	Std. Error Mean
Pair 1	Belife.3	1.3571	70	.53934	.06446
	belife.3.post	2.3571	70	.61469	.07347

Paired Samples Correlations

		N	Correlation	Sig.
Pair 1	Belife.3 & belife.3.post	70	-.041	.739

Paired Samples Test

		Paired Differences			
					95% Confidence ...
		Mean	Std. Deviation	Std. Error Mean	Lower
Pair 1	Belife.3 - belife.3.post	-1.00000	.83406	.09969	-1.19887

Paired Samples Test

		Paired ...			
		95% Confidence Interval of the ...			
		Upper	t	df	Sig. (2-tailed)
Pair 1	Belife.3 - belife.3.post	-.80113	-10.031	69	.000

```
 T-TEST PAIRS=Belife.4 WITH belife.4.post (PAIRED)
/CRITERIA=CI(.95)
/MISSING=ANALYSIS.
```

T-Test

Notes

Output Created		19-SEP-2026 09:38:18
Comments		
Input	Data	F:\\ \asd\ \major.revision.9.2026\ASD_Deidentified_Dataset.sav
	Active Dataset	DataSet1
	Filter	group = 1 (FILTER)
	Weight	<none>
	Split File	<none>
	N of Rows in Working Data File	70
Missing Value Handling	Definition of Missing	User defined missing values are treated as missing.
	Cases Used	Statistics for each analysis are based on the cases with no missing or out-of-range data for any variable in the analysis.
Syntax		T-TEST PAIRS=Belife.4 WITH belife.4.post (PAIRED) /CRITERIA=CI(.95) /MISSING=ANALYSIS.
Resources	Processor Time	00:00:00.00
	Elapsed Time	00:00:00.00

Paired Samples Statistics

		Mean	N	Std. Deviation	Std. Error Mean
Pair 1	Belife.4	1.4429	70	.58075	.06941
	belife.4.post	2.4857	70	.60775	.07264

Paired Samples Correlations

		N	Correlation	Sig.
Pair 1	Belife.4 & belife.4.post	70	-.002	.985

Paired Samples Test

		Paired Differences			
					95% Confidence ...
		Mean	Std. Deviation	Std. Error Mean	Lower
Pair 1	Belife.4 - belife.4.post	-1.04286	.84159	.10059	-1.24353

Paired Samples Test

		Paired ...			
		95% Confidence Interval of the ...			
		Upper	t	df	Sig. (2-tailed)
Pair 1	Belife.4 - belife.4.post	-.84219	-10.367	69	.000

```
 T-TEST PAIRS=Belife.5 WITH belife.5.post (PAIRED)
/CRITERIA=CI(.95)
/MISSING=ANALYSIS.
```

T-Test

Notes

Output Created		19-SEP-2026 09:38:18
Comments		
Input	Data	F:\\ \asd\ \major.revision. 9.2026 \ASD_Deidentified_Dataset.sav
	Active Dataset	DataSet1
	Filter	group = 1 (FILTER)
	Weight	<none>
	Split File	<none>
	N of Rows in Working Data File	70
Missing Value Handling	Definition of Missing	User defined missing values are treated as missing.
	Cases Used	Statistics for each analysis are based on the cases with no missing or out-of-range data for any variable in the analysis.
Syntax		T-TEST PAIRS=Belife.5 WITH belife.5.post (PAIRED) /CRITERIA=CI(.95) /MISSING=ANALYSIS.
Resources	Processor Time	00:00:00.00
	Elapsed Time	00:00:00.00

Paired Samples Statistics

		Mean	N	Std. Deviation	Std. Error Mean
Pair 1	Belife.5	1.4000	70	.57483	.06871
	belife.5.post	2.5714	70	.60365	.07215

Paired Samples Correlations

		N	Correlation	Sig.
Pair 1	Belife.5 & belife.5.post	70	.084	.492

Paired Samples Test

		Paired Differences			
					95% Confidence ...
		Mean	Std. Deviation	Std. Error Mean	Lower
Pair 1	Belife.5 - belife.5.post	-1.17143	.79803	.09538	-1.36171

Paired Samples Test

		Paired ...			
		95% Confidence Interval of the ...			
		Upper	t	df	Sig. (2-tailed)
Pair 1	Belife.5 - belife.5.post	-.98114	-12.281	69	.000

```
 T-TEST PAIRS=Belife.6 WITH belife.6.post (PAIRED)
/CRITERIA=CI(.95)
/MISSING=ANALYSIS.
```

T-Test

Notes

Output Created		19-SEP-2026 09:38:18
Comments		
Input	Data	F:\\ \asd\ \major.revision.9.2026\ASD_Deidentified_Dataset.sav
	Active Dataset	DataSet1
	Filter	group = 1 (FILTER)
	Weight	<none>
	Split File	<none>
	N of Rows in Working Data File	70
Missing Value Handling	Definition of Missing	User defined missing values are treated as missing.
	Cases Used	Statistics for each analysis are based on the cases with no missing or out-of-range data for any variable in the analysis.
Syntax		T-TEST PAIRS=Belife.6 WITH belife.6.post (PAIRED) /CRITERIA=CI(.95) /MISSING=ANALYSIS.
Resources	Processor Time	00:00:00.02
	Elapsed Time	00:00:00.02

Paired Samples Statistics

		Mean	N	Std. Deviation	Std. Error Mean
Pair 1	Belife.6	1.4286	70	.60365	.07215
	belife.6.post	2.5857	70	.57717	.06899

Paired Samples Correlations

		N	Correlation	Sig.
Pair 1	Belife.6 & belife.6.post	70	.059	.625

Paired Samples Test

		Paired Differences			
					95% Confidence ...
		Mean	Std. Deviation	Std. Error Mean	Lower
Pair 1	Belife.6 - belife.6.post	-1.15714	.81000	.09681	-1.35028

Paired Samples Test

		Paired ...			
		95% Confidence Interval of the ...			
		Upper	t	df	Sig. (2-tailed)
Pair 1	Belife.6 - belife.6.post	-.96400	-11.952	69	.000

```
 T-TEST PAIRS=Belife.7 WITH belife.7.post (PAIRED)
/CRITERIA=CI(.95)
/MISSING=ANALYSIS.
```

T-Test

Notes

Output Created		19-SEP-2026 09:38:18
Comments		
Input	Data	F:\\ \asd\ \major.revision. 9.2026 \ASD_Deidentified_Dataset.sav
	Active Dataset	DataSet1
	Filter	group = 1 (FILTER)
	Weight	<none>
	Split File	<none>
	N of Rows in Working Data File	70
Missing Value Handling	Definition of Missing	User defined missing values are treated as missing.
	Cases Used	Statistics for each analysis are based on the cases with no missing or out-of-range data for any variable in the analysis.
Syntax		T-TEST PAIRS=Belife.7 WITH belife.7.post (PAIRED) /CRITERIA=CI(.95) /MISSING=ANALYSIS.
Resources	Processor Time	00:00:00.00
	Elapsed Time	00:00:00.00

Paired Samples Statistics

		Mean	N	Std. Deviation	Std. Error Mean
Pair 1	Belife.7	1.4000	70	.57483	.06871
	belife.7.post	2.5429	70	.65244	.07798

Paired Samples Correlations

		N	Correlation	Sig.
Pair 1	Belife.7 & belife.7.post	70	-.046	.703

Paired Samples Test

		Paired Differences			
					95% Confidence ...
		Mean	Std. Deviation	Std. Error Mean	Lower
Pair 1	Belife.7 - belife.7.post	-1.14286	.88932	.10629	-1.35491

Paired Samples Test

		Paired ...			
		95% Confidence Interval of the ...			
		Upper	t	df	Sig. (2-tailed)
Pair 1	Belife.7 - belife.7.post	-.93081	-10.752	69	.000

```
 T-TEST PAIRS=Belife.8 WITH belife.8.post (PAIRED)
/CRITERIA=CI(.95)
/MISSING=ANALYSIS.
```

T-Test

Notes

Output Created		19-SEP-2026 09:38:18
Comments		
Input	Data	F:\\ \asd\ \major.revision. 9.2026 \ASD_Deidentified_Dataset.sav
	Active Dataset	DataSet1
	Filter	group = 1 (FILTER)
	Weight	<none>
	Split File	<none>
	N of Rows in Working Data File	70
Missing Value Handling	Definition of Missing	User defined missing values are treated as missing.
	Cases Used	Statistics for each analysis are based on the cases with no missing or out-of-range data for any variable in the analysis.
Syntax		T-TEST PAIRS=Belife.8 WITH belife.8.post (PAIRED) /CRITERIA=CI(.95) /MISSING=ANALYSIS.
Resources	Processor Time	00:00:00.00
	Elapsed Time	00:00:00.00

Paired Samples Statistics

		Mean	N	Std. Deviation	Std. Error Mean
Pair 1	Belife.8	1.3429	70	.50750	.06066
	belife.8.post	2.3429	70	.65686	.07851

Paired Samples Correlations

		N	Correlation	Sig.
Pair 1	Belife.8 & belife.8.post	70	-.010	.935

Paired Samples Test

		Paired Differences			
					95% Confidence ...
		Mean	Std. Deviation	Std. Error Mean	Lower
Pair 1	Belife.8 - belife.8.post	-1.00000	.83406	.09969	-1.19887

Paired Samples Test

		Paired ...			
		95% Confidence Interval of the ...			
		Upper	t	df	Sig. (2-tailed)
Pair 1	Belife.8 - belife.8.post	-.80113	-10.031	69	.000

```
GLM total.knowledge.micro.and.tradition.pre
   total.knowledge.micro.and.tradition.post BY group
   /WSFACTOR=Time 2 Polynomial
   /METHOD=SSTYPE(3)
   /PLOT=PROFILE(Time*group)
   /EMMEANS=TABLES(Time*group) COMPARE(Time) ADJ(BONFERRONI)
   /PRINT=DESCRIPTIVE ETASQ
   /CRITERIA=ALPHA(.05)
   /WSDESIGN=Time
   /DESIGN=group.
```

General Linear Model

Notes

Output Created		19-SEP-2026 09:51:28
Comments		
Input	Data	F:\\ \asd\ \major.revision. 9.2026 \ASD_Deidentified_Dataset.sav
	Active Dataset	DataSet1
	Filter	<none>
	Weight	<none>
	Split File	<none>
	N of Rows in Working Data File	140
Missing Value Handling	Definition of Missing	User-defined missing values are treated as missing.
	Cases Used	Statistics are based on all cases with valid data for all variables in the model.
Syntax		GLM total.knowledge.micro.and.tradition.pre total.knowledge.micro.and.tradition.post BY group /WSFACTOR=Time 2 Polynomial /METHOD=SSTYPE(3) /PLOT=PROFILE(Time*group) /EMMEANS=TABLES(Time*group) COMPARE(Time) ADJ(BONFERRONI) /PRINT=DESCRIPTIVE ETASQ /CRITERIA=ALPHA(.05) /WSDESIGN=Time...
Resources	Processor Time	00:00:02.66
	Elapsed Time	00:00:02.02

Within-Subjects Factors

Measure: MEASURE_1

Time	Dependent Variable
1	total.knowledge.micro.and.tradition.pre
2	total.knowledge.micro.and.tradition.post

Between-Subjects Factors

		Value Label	N
group	.00	micro learning	70
	1.00	traditional	70

Descriptive Statistics

	group	Mean	Std. Deviation	N
total.knowledge.micro.and.tradition.pre	micro learning	1.8429	1.80688	70
	traditional	1.7429	1.50059	70
	Total	1.7929	1.65558	140
total.knowledge.micro.and.tradition.post	micro learning	12.5000	2.95252	70
	traditional	9.0857	4.21434	70
	Total	10.7929	4.00987	140

Multivariate Tests[a]

Effect		Value	F	Hypothesis df	Error df	Sig.
Time	Pillai's Trace	.838	714.725[b]	1.000	138.000	.000
	Wilks' Lambda	.162	714.725[b]	1.000	138.000	.000
	Hotelling's Trace	5.179	714.725[b]	1.000	138.000	.000
	Roy's Largest Root	5.179	714.725[b]	1.000	138.000	.000
Time * group	Pillai's Trace	.149	24.231[b]	1.000	138.000	.000
	Wilks' Lambda	.851	24.231[b]	1.000	138.000	.000
	Hotelling's Trace	.176	24.231[b]	1.000	138.000	.000
	Roy's Largest Root	.176	24.231[b]	1.000	138.000	.000

Multivariate Tests[a]

Effect		Partial Eta Squared
Time	Pillai's Trace	.838
	Wilks' Lambda	.838
	Hotelling's Trace	.838
	Roy's Largest Root	.838
Time * group	Pillai's Trace	.149
	Wilks' Lambda	.149
	Hotelling's Trace	.149
	Roy's Largest Root	.149

a. Design: Intercept + group
Within Subjects Design: Time

b. Exact statistic

Mauchly's Test of Sphericity[a]

Measure: MEASURE_1

Within Subjects Effect	Mauchly's W	Approx. Chi-Square	df	Sig.	Epsilon[b] Greenhouse-Geisser
Time	1.000	.000	0	.	1.000

Mauchly's Test of Sphericity[a]

Measure: MEASURE_1

Within Subjects Effect	Epsilon[b] Huynh-Feldt	Lower-bound
Time	1.000	1.000

Tests the null hypothesis that the error covariance matrix of the orthonormalized transformed dependent variables is proportional to an identity matrix.

a. Design: Intercept + group
Within Subjects Design: Time

b. May be used to adjust the degrees of freedom for the averaged tests of significance. Corrected tests are displayed in the Tests of Within-Subjects Effects table.

Tests of Within-Subjects Effects

Measure: MEASURE_1

Source		Type III Sum of Squares	df	Mean Square	F	Sig.
Time	Sphericity Assumed	5670.000	1	5670.000	714.725	.000
	Greenhouse-Geisser	5670.000	1.000	5670.000	714.725	.000
	Huynh-Feldt	5670.000	1.000	5670.000	714.725	.000
	Lower-bound	5670.000	1.000	5670.000	714.725	.000
Time * group	Sphericity Assumed	192.229	1	192.229	24.231	.000
	Greenhouse-Geisser	192.229	1.000	192.229	24.231	.000
	Huynh-Feldt	192.229	1.000	192.229	24.231	.000
	Lower-bound	192.229	1.000	192.229	24.231	.000
Error(Time)	Sphericity Assumed	1094.771	138	7.933		
	Greenhouse-Geisser	1094.771	138.000	7.933		
	Huynh-Feldt	1094.771	138.000	7.933		
	Lower-bound	1094.771	138.000	7.933		

Tests of Within-Subjects Effects

Measure: MEASURE_1

Source		Partial Eta Squared
Time	Sphericity Assumed	.838
	Greenhouse-Geisser	.838
	Huynh-Feldt	.838
	Lower-bound	.838
Time * group	Sphericity Assumed	.149
	Greenhouse-Geisser	.149
	Huynh-Feldt	.149
	Lower-bound	.149
Error(Time)	Sphericity Assumed	
	Greenhouse-Geisser	
	Huynh-Feldt	
	Lower-bound	

Tests of Within-Subjects Contrasts

Measure: MEASURE_1

Source	Time	Type III Sum of Squares	df	Mean Square	F	Sig.
Time	Linear	5670.000	1	5670.000	714.725	.000
Time * group	Linear	192.229	1	192.229	24.231	.000
Error(Time)	Linear	1094.771	138	7.933		

Tests of Within-Subjects Contrasts

Measure: MEASURE_1

Source	Time	Partial Eta Squared
Time	Linear	.838
Time * group	Linear	.149
Error(Time)	Linear	

Tests of Between-Subjects Effects

Measure: MEASURE_1
Transformed Variable: Average

Source	Type III Sum of Squares	df	Mean Square	F	Sig.	Partial Eta Squared
Intercept	11088.014	1	11088.014	1374.971	.000	.909
group	216.129	1	216.129	26.801	.000	.163
Error	1112.857	138	8.064			

Estimated Marginal Means

Time * group

Estimates

Measure: MEASURE_1

Time	group	Mean	Std. Error	95% Confidence Interval	
				Lower Bound	Upper Bound
1	micro learning	1.843	.199	1.450	2.235
	traditional	1.743	.199	1.350	2.135
2	micro learning	12.500	.435	11.640	13.360
	traditional	9.086	.435	8.226	9.946

Pairwise Comparisons

Measure: MEASURE_1

group	(I) Time	(J) Time	Mean Difference (I-J)	Std. Error	Sig.[b]	95% Confidence ...[b]
						Lower Bound
micro learning	1	2	-10.657*	.476	.000	-11.599
	2	1	10.657*	.476	.000	9.716
traditional	1	2	-7.343*	.476	.000	-8.284
	2	1	7.343*	.476	.000	6.401

Pairwise Comparisons

Measure: MEASURE_1

group	(I) Time	(J) Time	95% Confidence Interval for ...[b] Upper Bound
micro learning	1	2	-9.716
	2	1	11.599
traditional	1	2	-6.401
	2	1	8.284

Based on estimated marginal means

*. The mean difference is significant at the .05 level.

b. Adjustment for multiple comparisons: Bonferroni.

Multivariate Tests

group		Value	F	Hypothesis df	Error df	Sig.
micro learning	Pillai's trace	.784	501.078[a]	1.000	138.000	.000
	Wilks' lambda	.216	501.078[a]	1.000	138.000	.000
	Hotelling's trace	3.631	501.078[a]	1.000	138.000	.000
	Roy's largest root	3.631	501.078[a]	1.000	138.000	.000
traditional	Pillai's trace	.633	237.878[a]	1.000	138.000	.000
	Wilks' lambda	.367	237.878[a]	1.000	138.000	.000
	Hotelling's trace	1.724	237.878[a]	1.000	138.000	.000
	Roy's largest root	1.724	237.878[a]	1.000	138.000	.000

Multivariate Tests

group		Partial Eta Squared
micro learning	Pillai's trace	.784
	Wilks' lambda	.784
	Hotelling's trace	.784
	Roy's largest root	.784
traditional	Pillai's trace	.633
	Wilks' lambda	.633
	Hotelling's trace	.633
	Roy's largest root	.633

Each F tests the multivariate simple effects of Time within each level combination of the other effects shown. These tests are based on the linearly independent pairwise comparisons among the estimated marginal means.

a. Exact statistic

Profile Plots

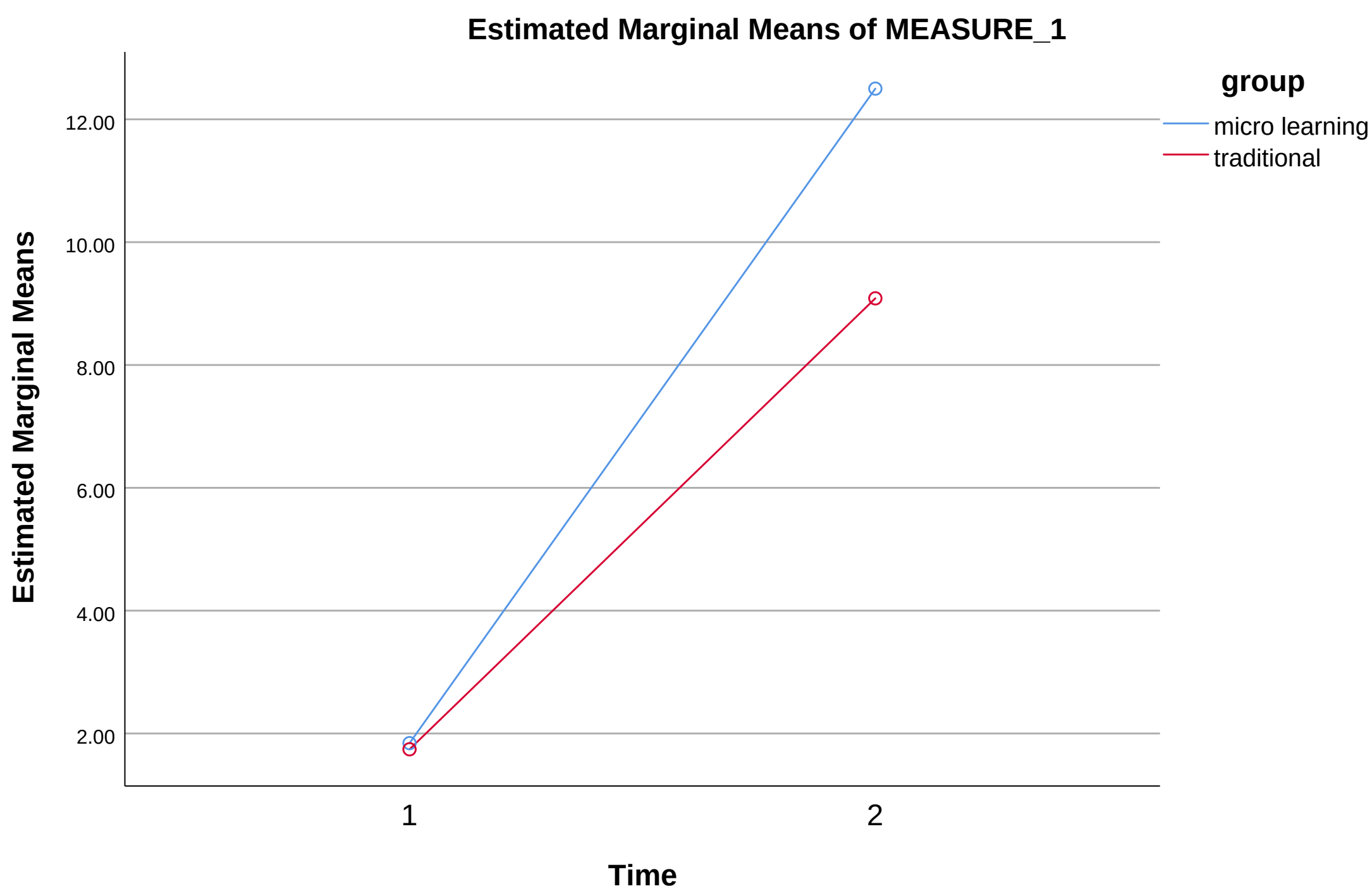


```
GLM total.belife.micro.and.tradition.pre
   total.belife.micro.and.tradition.post BY group
   /WSFACTOR=Time 2 Polynomial
   /METHOD=SSTYPE(3)
   /PLOT=PROFILE(Time*group)
   /EMMEANS=TABLES(Time*group) COMPARE(Time) ADJ(BONFERRONI)
   /PRINT=DESCRIPTIVE ETASQ
   /CRITERIA=ALPHA(.05)
   /WSDESIGN=Time
   /DESIGN=group.
```

General Linear Model

Notes

Output Created		19-SEP-2026 09:52:39
Comments		
Input	Data	F:\\ \asd\ \major.revision. 9.2026 \ASD_Deidentified_Dataset.sav
	Active Dataset	DataSet1
	Filter	<none>
	Weight	<none>
	Split File	<none>
	N of Rows in Working Data File	140
Missing Value Handling	Definition of Missing	User-defined missing values are treated as missing.
	Cases Used	Statistics are based on all cases with valid data for all variables in the model.
Syntax		GLM total.belife.micro.and.tradition.pre total.belife.micro.and.tradition.post BY group<br>/WSFACTOR=Time 2 Polynomial<br>/METHOD=SSTYPE(3)<br>/PLOT=PROFILE (Time*group)<br>/EMMEANS=TABLES (Time*group) COMPARE (Time) ADJ (BONFERRONI)<br>/PRINT=DESCRIPTIVE ETASQ<br>/CRITERIA=ALPHA(.05)<br>/WSDESIGN=Time...
Resources	Processor Time	00:00:00.59
	Elapsed Time	00:00:00.20

Within-Subjects Factors

Measure: MEASURE_1

Time	Dependent Variable
1	total.belife.micro.and.tradition.pre
2	total.belife.micro.and.tradition.post

Between-Subjects Factors

		Value Label	N
group	.00	micro learning	70
	1.00	traditional	70

Descriptive Statistics

	group	Mean	Std. Deviation	N
total.belife.micro.and.tradition.pre	micro learning	10.9000	3.83406	70
	traditional	11.1000	3.93111	70
	Total	11.0000	3.87020	140
total.belife.micro.and.tradition.post	micro learning	22.8857	2.55685	70
	traditional	19.8143	3.84592	70
	Total	21.3500	3.60041	140

Multivariate Tests[a]

Effect		Value	F	Hypothesis df	Error df	Sig.
Time	Pillai's Trace	.810	589.754[b]	1.000	138.000	.000
	Wilks' Lambda	.190	589.754[b]	1.000	138.000	.000
	Hotelling's Trace	4.274	589.754[b]	1.000	138.000	.000
	Roy's Largest Root	4.274	589.754[b]	1.000	138.000	.000
Time * group	Pillai's Trace	.096	14.730[b]	1.000	138.000	.000
	Wilks' Lambda	.904	14.730[b]	1.000	138.000	.000
	Hotelling's Trace	.107	14.730[b]	1.000	138.000	.000
	Roy's Largest Root	.107	14.730[b]	1.000	138.000	.000

Multivariate Tests[a]

Effect		Partial Eta Squared
Time	Pillai's Trace	.810
	Wilks' Lambda	.810
	Hotelling's Trace	.810
	Roy's Largest Root	.810
Time * group	Pillai's Trace	.096
	Wilks' Lambda	.096
	Hotelling's Trace	.096
	Roy's Largest Root	.096

a. Design: Intercept + group
Within Subjects Design: Time

b. Exact statistic

Mauchly's Test of Sphericity[a]

Measure: MEASURE_1

Within Subjects Effect	Mauchly's W	Approx. Chi-Square	df	Sig.	Epsilon[b] Greenhouse-Geisser
Time	1.000	.000	0	.	1.000

Mauchly's Test of Sphericity[a]

Measure: MEASURE_1

Within Subjects Effect	Epsilon[b] Huynh-Feldt	Epsilon[b] Lower-bound
Time	1.000	1.000

Tests the null hypothesis that the error covariance matrix of the orthonormalized transformed dependent variables is proportional to an identity matrix.

a. Design: Intercept + group
Within Subjects Design: Time

b. May be used to adjust the degrees of freedom for the averaged tests of significance. Corrected tests are displayed in the Tests of Within-Subjects Effects table.

Tests of Within-Subjects Effects

Measure: MEASURE_1

Source		Type III Sum of Squares	df	Mean Square	F	Sig.
Time	Sphericity Assumed	7498.575	1	7498.575	589.754	.000
	Greenhouse-Geisser	7498.575	1.000	7498.575	589.754	.000
	Huynh-Feldt	7498.575	1.000	7498.575	589.754	.000
	Lower-bound	7498.575	1.000	7498.575	589.754	.000
Time * group	Sphericity Assumed	187.289	1	187.289	14.730	.000
	Greenhouse-Geisser	187.289	1.000	187.289	14.730	.000
	Huynh-Feldt	187.289	1.000	187.289	14.730	.000
	Lower-bound	187.289	1.000	187.289	14.730	.000
Error(Time)	Sphericity Assumed	1754.636	138	12.715		
	Greenhouse-Geisser	1754.636	138.000	12.715		
	Huynh-Feldt	1754.636	138.000	12.715		
	Lower-bound	1754.636	138.000	12.715		

Tests of Within-Subjects Effects

Measure: MEASURE_1

Source		Partial Eta Squared
Time	Sphericity Assumed	.810
	Greenhouse-Geisser	.810
	Huynh-Feldt	.810
	Lower-bound	.810
Time * group	Sphericity Assumed	.096
	Greenhouse-Geisser	.096
	Huynh-Feldt	.096
	Lower-bound	.096
Error(Time)	Sphericity Assumed	
	Greenhouse-Geisser	
	Huynh-Feldt	
	Lower-bound	

Tests of Within-Subjects Contrasts

Measure: MEASURE_1

Source	Time	Type III Sum of Squares	df	Mean Square	F	Sig.
Time	Linear	7498.575	1	7498.575	589.754	.000
Time * group	Linear	187.289	1	187.289	14.730	.000
Error(Time)	Linear	1754.636	138	12.715		

Tests of Within-Subjects Contrasts

Measure: MEASURE_1

Source	Time	Partial Eta Squared
Time	Linear	.810
Time * group	Linear	.096
Error(Time)	Linear	

Tests of Between-Subjects Effects

Measure: MEASURE_1

Transformed Variable: Average

Source	Type III Sum of Squares	df	Mean Square	F	Sig.	Partial Eta Squared
Intercept	73256.575	1	73256.575	5623.724	.000	.976
group	144.289	1	144.289	11.077	.001	.074
Error	1797.636	138	13.026			

Estimated Marginal Means

Time * group

Estimates

Measure: MEASURE_1

				95% Confidence Interval	
Time	group	Mean	Std. Error	Lower Bound	Upper Bound
1	micro learning	10.900	.464	9.982	11.818
	traditional	11.100	.464	10.182	12.018
2	micro learning	22.886	.390	22.114	23.657
	traditional	19.814	.390	19.043	20.586

Pairwise Comparisons

Measure: MEASURE_1

						95% Confidence ...[b]
group	(I) Time	(J) Time	Mean Difference (I-J)	Std. Error	Sig.[b]	Lower Bound
micro learning	1	2	-11.986*	.603	.000	-13.177
	2	1	11.986*	.603	.000	10.794
traditional	1	2	-8.714*	.603	.000	-9.906
	2	1	8.714*	.603	.000	7.523

Pairwise Comparisons

Measure: MEASURE_1

group	(I) Time	(J) Time	95% Confidence Interval for b... Upper Bound
micro learning	1	2	-10.794
	2	1	13.177
traditional	1	2	-7.523
	2	1	9.906

Based on estimated marginal means

*. The mean difference is significant at the .05 level.

b. Adjustment for multiple comparisons: Bonferroni.

Multivariate Tests

group		Value	F	Hypothesis df	Error df	Sig.
micro learning	Pillai's trace	.741	395.447[a]	1.000	138.000	.000
	Wilks' lambda	.259	395.447[a]	1.000	138.000	.000
	Hotelling's trace	2.866	395.447[a]	1.000	138.000	.000
	Roy's largest root	2.866	395.447[a]	1.000	138.000	.000
traditional	Pillai's trace	.602	209.037[a]	1.000	138.000	.000
	Wilks' lambda	.398	209.037[a]	1.000	138.000	.000
	Hotelling's trace	1.515	209.037[a]	1.000	138.000	.000
	Roy's largest root	1.515	209.037[a]	1.000	138.000	.000

Multivariate Tests

group		Partial Eta Squared
micro learning	Pillai's trace	.741
	Wilks' lambda	.741
	Hotelling's trace	.741
	Roy's largest root	.741
traditional	Pillai's trace	.602
	Wilks' lambda	.602
	Hotelling's trace	.602
	Roy's largest root	.602

Each F tests the multivariate simple effects of Time within each level combination of the other effects shown. These tests are based on the linearly independent pairwise comparisons among the estimated marginal means.

a. Exact statistic

Profile Plots

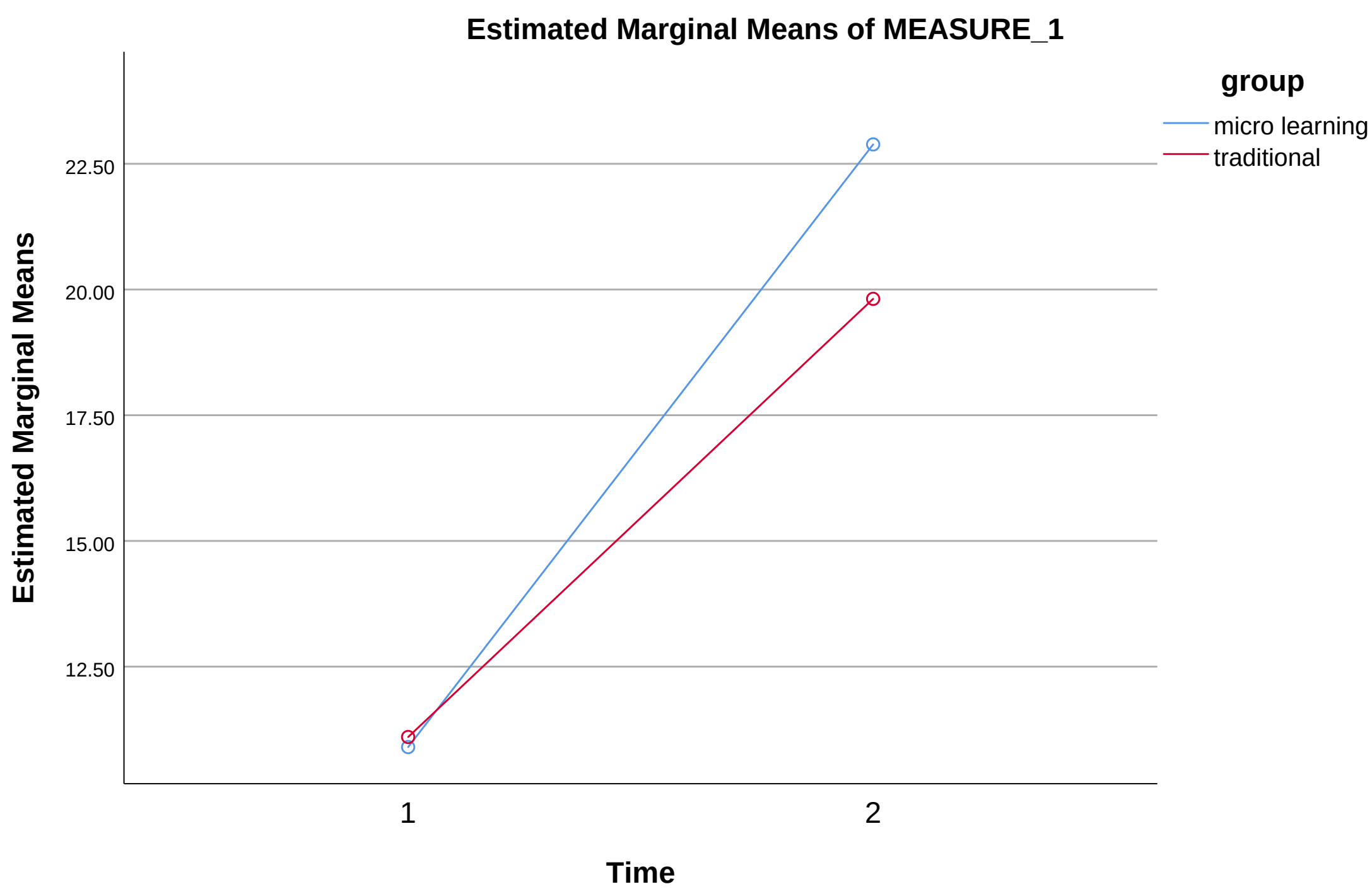

Supplement: Supplementary file 1 [file 12912_2026_5443_MOESM2_ESM.pdf]
