# Supplementary material for: Microlearning versus traditional instruction for improving nursing students’ knowledge and beliefs about autism spectrum disorder: a quasi-experimental study
Source: BMC Nurs. 2026 Oct 31;25:1050. doi: 10.1186/s12912-026-05443-9 (PMC13726028; doi:10.1186/s12912-026-05443-9)
Supplement: Supplementary file 2 [file 12912_2026_5443_MOESM7_ESM.pdf]

```
* Encoding: UTF-8.
* ==============================================================.
* ASD Study - Statistical Analysis Syntax.
* De-identified dataset: ASD_Deidentified_Dataset.sav.
* Group coding: 0 = Microlearning, 1 = Traditional.
* ==============================================================.


* ==============================================================.
* TABLE 1.
* Participant demographic and educational characteristics.
* ==============================================================.

* Categorical characteristics by study group.
CROSSTABS
  /TABLES=group BY age
  /STATISTICS=CHISQ
  /CELLS=COUNT ROW EXPECTED
  /COUNT ROUND CELL
  /EXACT=FISHER.

CROSSTABS
  /TABLES=group BY gender
  /STATISTICS=CHISQ
  /CELLS=COUNT ROW EXPECTED
  /COUNT ROUND CELL
  /EXACT=FISHER.

CROSSTABS
  /TABLES=group BY education
  /STATISTICS=CHISQ
  /CELLS=COUNT ROW EXPECTED
  /COUNT ROUND CELL
  /EXACT=FISHER.

CROSSTABS
  /TABLES=group BY previous
  /STATISTICS=CHISQ
  /CELLS=COUNT ROW EXPECTED
  /COUNT ROUND CELL
  /EXACT=FISHER.

CROSSTABS
  /TABLES=group BY family
  /STATISTICS=CHISQ
  /CELLS=COUNT ROW EXPECTED
  /COUNT ROUND CELL
```

```
  /EXACT=FISHER.

CROSSTABS
  /TABLES=group BY attend
  /STATISTICS=CHISQ
  /CELLS=COUNT ROW EXPECTED
  /COUNT ROUND CELL
  /EXACT=FISHER.

CROSSTABS
  /TABLES=group BY provided
  /STATISTICS=CHISQ
  /CELLS=COUNT ROW EXPECTED
  /COUNT ROUND CELL
  /EXACT=FISHER.


* Continuous age comparison between groups.
T-TEST GROUPS=group(0 1)
/VARIABLES=actual.age
/CRITERIA=CI(.95).


* ============================================================.
* TABLES 2 AND 3.
* McNemar tests for individual knowledge items.
* Pretest versus posttest within each study group.
* ============================================================.

* Microlearning group.
TEMPORARY.
SELECT IF group=0.

CROSSTABS
  /TABLES=q.1.pre BY q.1.post
  /TABLES=q.2.pre BY q.2.post
  /TABLES=q.3.pre BY q.3.post
  /TABLES=q.4.pre BY q.4.post
  /TABLES=q.5.pre BY q.5.post
  /TABLES=q.6.pre BY q.6.post
  /TABLES=q.7.pre BY q.7.post
  /TABLES=q.8.pre BY q.8.post
  /TABLES=q.9.pre BY q.9.post
  /TABLES=q.10.pre BY q.10.post
  /TABLES=q.11.pre BY q.11.post
  /TABLES=q.12.pre BY q.12..post
  /TABLES=q.13.pre BY q.13.post
```

```
  /TABLES=q.14.pre BY q.14.post
  /STATISTICS=MCNEMAR
  /CELLS=COUNT ROW.


* Traditional learning group.
TEMPORARY.
SELECT IF group=1.

CROSSTABS
  /TABLES=q.1.pre BY q.1.post
  /TABLES=q.2.pre BY q.2.post
  /TABLES=q.3.pre BY q.3.post
  /TABLES=q.4.pre BY q.4.post
  /TABLES=q.5.pre BY q.5.post
  /TABLES=q.6.pre BY q.6.post
  /TABLES=q.7.pre BY q.7.post
  /TABLES=q.8.pre BY q.8.post
  /TABLES=q.9.pre BY q.9.post
  /TABLES=q.10.pre BY q.10.post
  /TABLES=q.11.pre BY q.11.post
  /TABLES=q.12.pre BY q.12..post
  /TABLES=q.13.pre BY q.13.post
  /TABLES=q.14.pre BY q.14.post
  /STATISTICS=MCNEMAR
  /CELLS=COUNT ROW.


* ===============================================================.
* TABLE 4.
* Mixed-design repeated-measures ANOVA for total knowledge.
* Within-subject factor: Time (Pretest, Posttest).
* Between-subject factor: Group (Microlearning, Traditional).
* ===============================================================.

GLM total.knowledge.micro.and.tradition.pre
    total.knowledge.micro.and.tradition.post BY group
  /WSFACTOR=time 2 Polynomial
  /METHOD=SSTYPE(3)
  /PRINT=DESCRIPTIVE ETASQ
  /EMMEANS=TABLES(time*group)
  /CRITERIA=ALPHA(.05)
  /WSDESIGN=time
  /DESIGN=group.


* ===============================================================.
```

```
* TABLE 5.
* Paired-samples t-tests for individual belief items and
* total belief score within each study group.
* ==============================================================.

* Microlearning group.
TEMPORARY.
SELECT IF group=0.

T-TEST PAIRS=
  Belife.1 WITH belife.1.post
  Belife.2 WITH belife.2.post
  Belife.3 WITH belife.3.post
  Belife.4 WITH belife.4.post
  Belife.5 WITH belife.5.post
  Belife.6 WITH belife.6.post
  Belife.7 WITH belife.7.post
  Belife.8 WITH belife.8.post
  total.belife.micro.and.tradition.pre
  WITH total.belife.micro.and.tradition.post
  (PAIRED)
/CRITERIA=CI(.95).


* Traditional learning group.
TEMPORARY.
SELECT IF group=1.

T-TEST PAIRS=
  Belife.1 WITH belife.1.post
  Belife.2 WITH belife.2.post
  Belife.3 WITH belife.3.post
  Belife.4 WITH belife.4.post
  Belife.5 WITH belife.5.post
  Belife.6 WITH belife.6.post
  Belife.7 WITH belife.7.post
  Belife.8 WITH belife.8.post
  total.belife.micro.and.tradition.pre
  WITH total.belife.micro.and.tradition.post
  (PAIRED)
/CRITERIA=CI(.95).


* ==============================================================.
* TABLE 6.
* Mixed-design repeated-measures ANOVA for total beliefs.
* Within-subject factor: Time (Pretest, Posttest).
```

```
* Between-subject factor: Group (Microlearning, Traditional).
* ==============================================================.

GLM total.belife.micro.and.tradition.pre
    total.belife.micro.and.tradition.post BY group
  /WSFACTOR=time 2 Polynomial
  /METHOD=SSTYPE(3)
  /PRINT=DESCRIPTIVE ETASQ
  /EMMEANS=TABLES(time*group)
  /CRITERIA=ALPHA(.05)
  /WSDESIGN=time
  /DESIGN=group.


* ==============================================================.
* END OF STATISTICAL ANALYSIS SYNTAX.
* ==============================================================.
```
