# Supplementary material for: Microlearning versus traditional instruction for improving nursing students’ knowledge and beliefs about autism spectrum disorder: a quasi-experimental study
Source: BMC Nurs. 2026 Oct 31;25:1050. doi: 10.1186/s12912-026-05443-9 (PMC13726028; doi:10.1186/s12912-026-05443-9)
Supplement: Supplementary file 3 [file 12912_2026_5443_MOESM1_ESM.docx]

An online questionnaire [link](https://docs.google.com/forms/d/e/1FAIpQLSdvpqDvzXu1tj4KBRuJtLuvgVCRHIZJTaRr8DEw84SCvIc4vQ/viewform?usp=dialog) was sent to the participating nursing students according to their group to collect data on their characteristics, knowledge, and beliefs using the following study tools.

**Tool (I) An Online Structured Interview Questionnaire**

This tool was developed by the researchers after reviewing relevant literature, previous studies, and research ***by Gore et al. 2024 and Tang 2023.*** It was written in the English language and concerned the characteristics of the studied nursing students.

1- **Age/ years	……………………….year**
a. 19 ≤ 24
b. 25 ≤ 29
c. 30 ≤ 35
d. More than 35
2- **Gender**
a. Male
b. Female
3- **Academic Enrollment Status**
a. Regular program
b. Bridging program
4- **Have you previously studied content related to autism spectrum disorder (ASD) in any course?**
a. Yes
b. No
5- **Do you have a family member or relative diagnosed with ASD?**
a. Yes
b. No
6- **Have you attended any ASD-related training, workshop, or seminar?**
a. Yes
b. No
7- **Have you ever provided direct care or interaction with a person with ASD?**
a. Yes
b. No

**Tool (II): An Online Nursing Student ASD Knowledge Questionnaire.**

This tool was adopted from ***Abualhoul et al. 2024*** to assess pediatric nursing students’ knowledge about autism spectrum disorder, which includes14 items related to core characteristics, red flags, and interventions for autism spectrum disorder.

**Scoring System**

A scoring system was established in which each correct response was given a score of (1), and incorrect /don’t know was given a score of (0). Higher scores indicated more improvement in students' knowledge about children with ASD.

1. **Core Characteristics and Red Flags**

Each student was asked to read each item carefully and select one response from three options: **“True,” “False,” or “I don't know.**

| **Item No.** | **Statement** | **Key answer** |
| --- | --- | --- |
| K1 | Children with Autism Spectrum Disorder may show deficits in social reciprocity, such as difficulty with back-and-forth conversation. | Correct |
| K2 | Walking problems, such as difficulty climbing stairs, are a core feature of autism spectrum disorder. | Incorrect |
| K3 | Deficits in nonverbal communication, such as limited eye contact, are common in autism spectrum disorder. | Correct |
| K4 | Difficulties in developing and maintaining relationships are characteristic of autism spectrum disorder. | Correct |
| K5 | Repetitive behaviors, such as lining up toys, may be observed in children with Autism Spectrum Disorder. | Correct |
| K6 | Rigid adherence to routines, such as insisting on taking the same route to school, is common in autism spectrum disorder. | Correct |
| K7 | Children with Autism Spectrum Disorder may display highly restricted or fixated interests, such as focusing on specific objects only. | Correct |
| K8 | Unusual responses to sensory input, such as covering ears in response to certain sounds, are associated with autism spectrum disorder. | Correct |
| K9 | Delayed toilet training is a core diagnostic red flag of autism spectrum disorder. | Incorrect |

**B. Knowledge of ASD Interventions**

Each student was asked to read each item carefully and select one response from three options: **“True,” “False,” or “I don't know.**

| **Item No.** | **Statement** | **Item Polarity** |
| --- | --- | --- |
| K10 | Psychosocial interventions play an important role in the management of autism spectrum disorder. | Correct |
| K11 | Pharmacological treatment may be used to manage associated symptoms of autism spectrum disorder. | Correct |
| K12 | Dietary interventions alone are an evidence-based treatment for autism spectrum disorder. | Incorrect |
| K13 | Hyperbaric oxygen therapy is an evidence-based treatment for autism spectrum disorder. | Incorrect |
| K14 | Behavioral, developmental, and multidisciplinary educational interventions are effective approaches for managing autism spectrum disorder. | Correct |

**Tool (III): An online nursing student beliefs questionnaire.**

This tool was adapted from ***Abualhoul et al. (2024)*** to assess pediatric nursing students' beliefs about autism spectrum disorder, and some modifications were made, including the deletion of two items to avoid redundancy, as they were already covered in the knowledge questionnaire; it consists of eight items related to autism spectrum disorder.

**Scoring system**

The total beliefs score was rated using a 3-point Likert scale, ranging from 1 (Disagree), 2 (Neutral), to 3 (Agree). For negatively worded statements, including: Most children with ASD have intellectual disability, ASD occurs more commonly among children from higher socioeconomic backgrounds, and ASD is a preventable condition, the scores were reverse-coded before analysis. Higher scores indicated more positive beliefs toward children with ASD.

| **Item No.** | **Statement** | **Item Polarity** |
| --- | --- | --- |
| B1 | Autism Spectrum Disorder is a neurodevelopmental disorder. | Positive |
| B2 | Most children with autism spectrum disorder have intellectual disability. | Negative  Reversed coded |
| B3 | Autism Spectrum Disorder occurs more commonly among children from higher socioeconomic backgrounds. | Negative  Reversed coded |
| B4 | Autism Spectrum Disorder is often under-recognized and missed in general healthcare practice. | Positive |
| B5 | There is insufficient awareness of autism spectrum disorder among healthcare providers. | Positive |
| B6 | Autism Spectrum Disorder is diagnosed more frequently in males than in females. | Positive |
| B7 | Autism Spectrum Disorder is a lifelong condition. | Positive |
| B8 | Autism Spectrum Disorder is a preventable condition. | Negative  Reversed coded |

**Reference**

1. Abualhoul, M., Abd Aziz, A., & Yahaya, R. (2024). Validation of knowledge, awareness, experience, and belief on early detection of autism spectrum disorder among healthcare providers in Jordan. *International Research Journal of Modernization in Engineering, Technology and Science, 6*(1). <https://doi.org/10.56726/IRJMETS48022>
2. Gore, K., Gilbert, M., Hawke, M., & Barbaro, J. (2024). Investigating autism knowledge, self-efficacy, and confidence following maternal and child health nurse training for the early identification of autism. *Frontiers in Neurology*, *14*, 1201292. <https://doi.org/10.3389/fneur.2023.1201292>
3. Tang, K. H. D. (2023). Student-centered approach in teaching and learning: What does it really mean?. *Acta Pedagogia Asiana*, *2*(2), 72-83.<https://doi.org/10.53623/apga.v2i2.218>
