# Supplementary material for: Microlearning versus traditional instruction for improving nursing students’ knowledge and beliefs about autism spectrum disorder: a quasi-experimental study
Source: BMC Nurs. 2026 Oct 31;25:1050. doi: 10.1186/s12912-026-05443-9 (PMC13726028; doi:10.1186/s12912-026-05443-9)
Supplement: Supplementary file 4 [file 12912_2026_5443_MOESM5_ESM.docx]

| **Session** | **Educational Content** | **Microlearning Group (n = 70)** | **Traditional Instruction Group (n = 70)** | **Evaluation After the Session** |
| --- | --- | --- | --- | --- |
| 1 | Definition & Spectrum Continuum | Asynchronous instructional video uploaded to the online Moodle learning platform students accessed the content at their own pace. | Synchronous 30-minute PowerPoint-based lecture delivered separately to three subgroups (25, 25, and 20 students) according to their attendance schedules. | **Microlearning**: Interactive formative questions/quiz with immediate automated feedback.  **Traditional**: Oral questioning and classroom discussion to check students’ understanding. |
| 2 | DSM-5 Social Deficits (Part 1) | Asynchronous instructional video addressing DSM-5 social deficits (Part 1) | Synchronous 30-minute PowerPoint-based lecture covering the same content, repeated for the three subgroups. | **Microlearning**: Post-video formative quiz with immediate feedback and answer rationales.  **Traditional:** Oral questions and discussion during/at the end of the lecture. |
| 3 | DSM-5 Social Deficits (Part 2) | Asynchronous instructional video addressing DSM-5 social deficits (Part 2). | Synchronous 30-minute PowerPoint-based lecture covering the corresponding content. | **Microlearning**: Interactive formative questions/quiz with automated feedback.  **Traditional:** Oral questioning and discussion. |
| 4 | Restricted/Repetitive Behaviors | Asynchronous instructional video addressing restricted and repetitive behaviors. | Synchronous 30-minute PowerPoint-based lecture covering the same content. | **Microlearning**: Post-video formative quiz with immediate feedback.  **Traditional:** Oral questioning and discussion. |
| 5 | Sensory Differences & Routines | Asynchronous instructional video addressing sensory differences and routines. | Synchronous: 30-minute PowerPoint-based lecture covering the same content. | **Microlearning:** Interactive formative questions/quiz with automated feedback. **Traditional:** Oral questioning and discussion. |
| 6 | Severity Levels (1–3) & Diagnostic Criteria | Asynchronous instructional video covering ASD severity levels and diagnostic criteria. | Synchronous 30-minute PowerPoint-based lecture covering the same content. | **Microlearning:** Post-video formative quiz with immediate feedback and answer rationales.  **Traditional:** Oral questioning and discussion. |
| 7 | Differential Diagnosis (SCD, ADHD, ID) | Asynchronous instructional video covering differential diagnosis. | Synchronous 30-minute PowerPoint-based lecture covering SCD, ADHD, and ID. | **Microlearning:** Interactive formative quiz with automated feedback.  **Traditional:** Oral questioning and discussion. |
| 8 | Screening Tools (M-CHAT-R/F & ASQ-3) | Asynchronous instructional video introducing screening tools. | Synchronous 30-minute PowerPoint-based lecture introducing the same screening tools. | **Microlearning:** Post-video formative questions/quiz with immediate feedback.  **Traditional:** Oral questioning and discussion. |
| 9 | Evidence-Based vs. Alternative Interventions | Asynchronous instructional video addressing evidence-based and alternative interventions. | Synchronous 30-minute PowerPoint-based lecture covering the same content. | **Microlearning:** Interactive formative quiz with immediate feedback and answer rationales.  **Traditional:** Oral questioning and discussion. |

**Supplementary Table S1. Session-by-Session Description of the Educational Intervention**
